# Meeting abstracts from the 9th International Conference on cGMP: Generators, Effectors and Therapeutic Implications

**DOI:** 10.1186/s12967-019-1994-0

**Published:** 2019-08-15

## S 1-01 NO-sGC signaling and therapeutics in sickle cell and hemolytic diseases

### Mark Gladwin^1,2^

#### ^1^University of Pittsburgh, Pittsburgh Heart, Lung, Blood and Vascular Medicine Institut, Pittsburgh Pennsylvania, US; ^2^University of Pittsburgh, Division of Pulmonary, Allergy and Critical Care Medicine, Department of Medicine, Pittsburgh Pennsylvania, US

##### **Correspondence:** Mark Gladwin - gladwinmt@upmc.edu

*Journal of Translational Medicine* 2019, **17(2):**S 1-01

**Introduction:** Nitric oxide (NO) is a critical regulator of vascular homeostasis, increasing basal and flow-mediated vasodilation. Red blood cells can regulate NO bioavailability through an intrinsic nitrite (NO_2−_) reductase mechanism that generates NO during physiological and pathological hypoxia. In contrast, red blood cell hemolysis releases hemoglobin into plasma, where can react with and scavenge NO. In this context, hemoglobin functions as an oxido-reductase: A nitrite-reductase and NO synthase when it deoxygenates and an NO oxidase when it is oxygenated, particularly in the setting of hemolysis. The mechanistic linkage between hemolytic anemia and vasculopathy has been the subject of extensive study in pre-clinical animal models, in vascular studies in patients, and in large human cohort studies. Intravascular hemolysis releases cell-free hemoglobin into the plasma, which can scavenge NO and generate reactive oxygen species, impairing redox balance and leading to proliferative systemic and pulmonary vasculopathy. Pre-clinical studies also suggest that sGC may be oxidized in sickle cell disease, and responsive to sGC activator therapy. It has also been recently appreciated that products released from the red cell during hemolysis, including heme released from hemoglobin, can be considered danger associated molecular pattern molecules or erythrocyte “DAMPs” (eDAMPs). Large screening studies of patients with sickle cell disease (SCD) for the presence of pulmonary hypertension (PH) have been performed using non-invasive Doppler-echocardiography, screening biomarkers such as N-terminal brain natriuretic peptide and right heart catheterization. These studies have reported a high prevalence of PH in this population, a significant association of increasing pulmonary pressures with more severe hemolytic anemia, cutaneous leg ulcerations, systemic systolic hypertension and renal dysfunction, and a high prospective associated risk of death. These studies support a more general pathological role for intravascular hemolysis and cell-free hemoglobin in various human diseases and in transfusion medicine.

**References**
1. Ataga KI, Moore CG, Jones S, Olajide O, Strayhorn D, Hinderliter A, Orringer EP. Pulmonary hypertension in patients with sickle cell disease: a longitudinal study. Br J Haematol. 2006;134:109–15.
2. De Castro LM, Jonassaint JC, Graham FL, Ashley-Koch A and Telen MJ. Pulmonary hypertension associated with sickle cell disease: clinical and laboratory endpoints and disease outcomes. Am J Hematol. 2008;83:19–25.
3. Gladwin MT, Sachdev V, Jison ML, Shizukuda Y, Plehn JF, Minter K, Brown B, Coles WA, Nichols JS, Ernst I, Hunter LA, Blackwelder WC, Schechter AN, Rodgers GP, Castro O and Ognibene FP. Pulmonary hypertension as a risk factor for death in patients with sickle cell disease. N Engl J Med. 2004;350:886–95.
4. Machado RF, Anthi A, Steinberg MH, Bonds D, Sachdev V, Kato GJ, Taveira-DaSilva AM, Ballas SK, Blackwelder W, Xu X, Hunter L, Barton B, Waclawiw M, Castro O and Gladwin MT. N-terminal pro-brain natriuretic peptide levels and risk of death in sickle cell disease. JAMA. 2006;296:310–8.
5. Mehari A, Alam S, Tian X, Cuttica MJ, Barnett CF, Miles G, Xu D, Seamon C, Adams-Graves P, Castro OL, Minniti CP, Sachdev V, Taylor JGt, Kato GJ, Machado RF. Hemodynamic predictors of mortality in adults with sickle cell disease. Am J Respir Crit Care Med. 2013;187:840–7.
6. Mehari A, Gladwin MT, Tian X, Machado RF, Kato GJ. Mortality in adults with sickle cell disease and pulmonary hypertension. JAMA. 2012;307:1254–6.
7. Fonseca GH, Souza R, Salemi VM, Jardim CV, Gualandro SF. Pulmonary hypertension diagnosed by right heart catheterisation in sickle cell disease. Eur Respir J. 2012;39:112–8.
8. Parent F, Bachir D, Inamo J, Lionnet F, Driss F, Loko G, Habibi A, Bennani S, Savale L, Adnot S, Maitre B, Yaici A, Hajji L, O’Callaghan DS, Clerson P, Girot R, Galacteros F, Simonneau G. A hemodynamic study of pulmonary hypertension in sickle cell disease. N Engl J Med. 2011;365:44–53.
9. Caughey MC, Poole C, Ataga KI, Hinderliter AL. Estimated pulmonary artery systolic pressure and sickle cell disease: a meta-analysis and systematic review. Br J Haematol. 2015;170:416–24.
10. Gladwin MT. Cardiovascular complications and risk of death in sickle-cell disease. Lancet. 2016;387:2565–74.
11. Reiter CD, Wang X, Tanus-Santos JE, Hogg N, Cannon RO, III, Schechter AN, Gladwin MT. Cell-free hemoglobin limits nitric oxide bioavailability in sickle-cell disease. Nat Med. 2002;8:1383–1389.
12. Rother RP, Bell L, Hillmen P, Gladwin MT. The clinical sequelae of intravascular hemolysis and extracellular plasma hemoglobin: a novel mechanism of human disease. JAMA. 2005;293:1653–62.
13. Gladwin MT, Ofori-Acquah SF. Erythroid DAMPs drive inflammation in SCD. Blood. 2014;123:3689–90.
14. Donadee C, Raat NJ, Kanias T, Tejero J, Lee JS, Kelley EE, Zhao X, Liu C, Reynolds H, Azarov I, Frizzell S, Meyer EM, Donnenberg AD, Qu L, Triulzi D, Kim-Shapiro DB, Gladwin MT. Nitric oxide scavenging by red blood cell microparticles and cell-free hemoglobin as a mechanism for the red cell storage lesion. Circulation. 2011;124:465–76.

## S 1-02 Evaluating soluble guanylate cyclase stimulation for serious central nervous system diseases

### Christopher Winrow, Juli Jones, Rajesh Iyengar, Susana Correia, Sarah Jacobson, Guang Liu, Peter Germano, Jose Trevejo, Mark Currie, John Hadcock, Todd Milne

#### Cyclerion Therapeutics, Cambridge Massachusetts, US

##### **Correspondence:** Todd Milne - tmilne@cyclerion.com

*Journal of Translational Medicine* 2019, **17(2):**S 1-02

**Introduction:** The nitric oxide (NO)-soluble guanylate cyclase (sGC)-cGMP signaling pathway plays a fundamental role in modulating diverse physiological processes within the central nervous system (CNS) including blood flow, inflammation, neuroprotection, neuronal signaling, and metabolism. sGC stimulators are small-molecule agonists of sGC that synergize with and enhance endogenous NO signaling. As such, sGC stimulators may provide therapeutic benefits in diseases with impaired NO signaling. Many neurodegenerative diseases have associated neurovascular impairment and inflammation that could be modulated with a CNS-penetrant sGC stimulator.

**Methods:** Despite the promise of this mechanism in the treatment of CNS diseases, there have been limited pharmacological tools available to modulate this pathway in the CNS. To evaluate the effects of a CNS-penetrant sGC stimulator (IW-6463), we conducted a series of nonclinical studies.

**Results:** IW-6463 suppressed markers of neuroinflammation in vivo and in vitro, enhanced fMRI-BOLD signals in brain regions associated with cognition, and increased qEEG gamma band signals in rodents. In chronic rodent studies with IW-6463, we observed improvements in dendritic spine density and morphology and restoration of cognitive performance. These nonclinical data support the hypothesis that CNS-penetrant sGC stimulators could potentially afford therapeutic benefit for neurodegenerative diseases.

**Conclusions:** Based on the association of impaired NO/sGC signalling with neurodegenerative diseases and the thorough nonclinical evaluation of IW-6463, we recently initiated a Phase 1 clinical study to evaluate the safety, tolerability, pharmacodynamics, and pharmacokinetics of IW-6463 in single- and multiple-ascending dose studies in healthy human volunteers.

## S 1-03 Efficacy and safety of riociguat in patients with early diffuse cutaneous systemic sclerosis: results from the RISE-SSc study, a randomized, double-blind, placebo-controlled phase IIb study

### Melanie Hemmrich^1^, Oliver Distler^2^, Yannick Allanore^3^, Christopher P Denton^4^, Masataka Kuwana^5^, Marco Matucci-Cerinic^6^, Janet E Pope^7^, Janethe de Oliveira Pena^8^, Kaisa Laapas^9^, Zhen Yao^10^, Dinesh Khanna^11^

#### ^1^Bayer AG, Wuppertal, North Rhine-Westphalia, Germany; ^2^Department of Rheumatology, University Hospital Zurich, Zurich, Switzerland; ^3^Rheumatology A Department, Cochin Hospital, Paris Descartes University, Sorbonne Paris Cité, Paris, France; ^4^UCL Division of Medicine, Royal Free Campus, London, United Kingdom; ^5^Department of Allergy and Rheumatology, Nippon Medical School Graduate School of Medicine, Tokyo, Japan; ^6^Department of Experimental and Clinical Medicine, University of Florence, Florence, Italy; ^7^Department of Medicine, Division of Rheumatology, University of Western Ontario, St. Joseph’s Health Care, London, Ontario, Canada; ^8^Bayer US LLC, Whippany, New Jersey, United States of America; ^9^StatFinn Oy, Espoo, Finland; ^10^Bayer Healthcare Co. Ltd, Beijing, China; ^11^Division of Rheumatology, Department of Internal Medicine, University of Michigan Scleroderma Program, University of Michigan, Ann Arbor, Michigan, United States of America

##### **Correspondence:** Melanie Hemmrich

*Journal of Translational Medicine* 2019, **17(2):**S 1-03

**Introduction:** There are no disease-modifying therapies for SSc or its more severe diffuse cutaneous form. The sGC stimulator rio showed vasodilatory, anti-fibrotic, and anti-remodeling properties in animal models and efficacy in patients (pts) with pulmonary arterial hypertension associated with connective tissue disease. It was therefore hypothesized that rio might provide benefit in pts with dcSSc. We present results from the RISE-SSc study (NCT02283762) of rio in pts with early dcSSc.

**Methods:** Inclusion criteria were: dcSSc (by 2013 ACR/EULAR criteria; LeRoy criteria), disease duration ≤ 18 months, modified Rodnan skin score (mRSS) ≥ 10 and ≤ 22 units (range 0–51, higher score is worse), forced vital capacity (FVC) ≥ 45% of predicted (%pred), and diffusing capacity of the lung for CO ≥ 40% pred at screening. Pts were randomized to pbo or rio (0.5–2.5 mg 3 times daily). The primary endpoint was change in mRSS from baseline (BL) to Week 52 (Wk52). Secondary endpoints included ACR Combined Response Index SSc (ACR CRISS), Health Assessment Questionnaire-Disability Index, and change in FVC%pred. A prespecified exploratory analysis of mRSS progression rate (increase of > 5 units and ≥ 25% from BL) and a post hoc analysis of lung function decline (FVC %pred decline ≥ 10% absolute) were performed. Further exploratory efficacy endpoints included changes in Raynaud’s phenomenon (RP) and digital ulcer (DU) burden.

**Results:** Overall, 121 pts (rio n = 60, pbo n = 61) were randomized (mean ± SD age 51 ± 12 years; 76% female). BL mRSS was comparable between rio and pbo (mean ± SD 16.9 ± 3.4 and 16.7 ± 4.1). At Wk52, mRSS was 14.6 ± 6.6 (rio) vs 15.7 ± 10.5 (pbo) (difference of least squares means [95% CI] − 2.3 [− 5.0, 0.3], p = 0.08). mRSS progression rate favored rio (− 18% [− 33.6, − 2.4], p = 0.02 [Mantel–Haenszel method]). ACR CRISS, HAQ DI, and FVC%pred showed no significant difference between the arms. At Wk52, lung function declined in 11% of rio pts vs 20% of pbo pts. Numeric trends of improvement of RP attacks/symptoms and prevention of new DU were seen with rio vs with pbo. Fewer serious adverse events occurred with rio vs pbo (15% vs 25% of pts, respectively). No new safety signals were observed.

**Conclusions:** The primary efficacy endpoint of mean change in mRSS did not reach statistical significance. Exploratory data showed signals for reduction of skin disease progression, and less decline of lung function, with rio in this early dcSSc population. There was a numeric trend toward reduced RP symptoms with rio compared with pbo, and vascular outcomes suggest that rio may reduce development of new DU recurrence in early dcSSc.

**Acknowledgement:** Funding for this study was provided by Bayer and Merck Sharp & Dohme Corp., a subsidiary of Merck & Co., Inc., Kenilworth, NJ, USA

## S 2-01 Targeting the *Plasmodium falciparum* cyclic GMP-dependent protein kinase to combat malaria

### David Baker

#### London School of Hygiene & Tropical Medicine, Faculty of Infectious and Tropical Diseases, London, UK

##### **Correspondence:** David Baker - david.baker@lshtm.ac.uk

*Journal of Translational Medicine* 2019, **17(2):**S 2-01

**Introduction:** Large-scale investment in malaria control programmes prior to the turn of the millennium led to very impressive reductions in the global numbers of cases of malaria and associated mortality. However, since 2015 the numbers have reached a plateau with more than 400,000 deaths each year, mainly amongst young children and pregnant women in developing countries. One of the reasons for this halt in progress is widespread drug resistance which makes it urgent to find new drugs that target alternative biochemical pathways in the malaria parasite *Plasmodium falciparum*. One promising target under investigation is the cGMP-dependent protein kinase (PKG) which is essential in all the key stages of the complex parasite life cycle [1]. It was observed firstly in related apicomplexan parasites (*Eimeria* and *Toxoplasma*) by a team at Merck that the parasite PKG has a key difference to mammalian PKG in the ATP binding site- it has a relatively small gatekeeper residue (a threonine) that was hypothesised to allow selective binding of certain inhibitor scaffolds [2].

**Methods:** A UK Medical Research Council-funded medicinal chemistry programme led by the London School of Hygiene & Tropical Medicine and LifeArc, focused on optimisation of an imidazopyridine scaffold (developed by Merck to treat *Eimeria* infections in chickens) against the malaria parasite *Plasmodium falciparum*.

**Results:** The most potent compound (ML10) from our programme [3] inhibited the kinase activity of recombinant *P. falciparum* PKG with an IC_50_ of 160 pM and importantly blocked blood stage malaria parasite development (which causes all the symptoms of malaria) in vitro with an EC_50_ of 2.1 nM. Furthermore ML10 has potent activity against sexual development (EC_50_ 41.3 nM) which is prerequisite for malaria transmission to mosquitoes. In vivo proof of concept for PKG as an antimalarial target was achieved with ML10 using an immunodeficient *P. falciparum* mouse model through partnership with GSK scientists in Tres Cantos. In collaboration with a team at the Structural Genomics Consortium in Toronto, a co-crystal structure of a malaria parasite PKG with ML10 was obtained revealing the intimate contacts that underlie the observed exquisite potency and selectivity and also confirming the necessity of the interaction with the small hydrophobic pocket conferred by the small gatekeeper residue that is unique to the apicomplexan parasite PKGs.

**Conclusions:** Further work will be needed to optimise this promising series.

**Acknowledgement:** We are grateful to the UK Medical Research Council, The Wellcome Trust, The Structural Genomics Consortium and the Tres Cantos Open Lab Foundation for funding this work.

**References**

1. Baker DA, Drought LG, Flueck C, Nofal SD, Patel A, Penzo M, Walker EM. Cyclic nucleotide signalling in malaria parasites. Open Biol. 2017;7(12). pii: 170213.
2. Donald RG, Zhong T, Wiersma H, Nare B, Yao D, Lee A, Allocco J, Liberator PA. Anticoccidial kinase inhibitors: identification of protein kinase targets secondary to cGMP-dependent protein kinase. Mol Biochem Parasitol. 2006;149(1):86–98.
3. Baker DA, Stewart LB, Large JM, Bowyer PW, Ansell KH, Jiménez-Díaz MB, El Bakkouri M, Birchall K, Dechering KJ, Bouloc NS, Coombs PJ, Whalley D, Harding DJ, Smiljanic-Hurley E, Wheldon MC, Walker EM, Dessens JT, Lafuente MJ, Sanz LM, Gamo FJ, Ferrer SB, Hui R, Bousema T, Angulo-Barturén I, Merritt AT, Croft SL, Gutteridge WE, Kettleborough CA, Osborne SA. A potent series targeting the malarial cGMP-dependent protein kinase clears infection and blocks transmission. Nat Commun. 2017;8(1):430.

## S 2-02 A unique activation mechanism for *Plasmodium falciparum* PKG

### Friedrich W. Herberg

#### Kassel University, Biochemistry, Kassel Hesse, Germany

##### **Correspondence:** Friedrich W. Herberg - herberg@uni-kassel.de

*Journal of Translational Medicine* 2019, **17(2):**S 2-02

**Introduction:** cGMP-dependent protein kinase from *Plasmodium falciparum* (*Pf*PKG) plays a crucial role in the sexual as well as the asexual proliferation of this malaria causing parasite. However, function and regulation of *Pf*PKG are largely unknown. Previous studies showed that the domain organization of PfPKG significantly differs from human PKG (*h*PKG) and indicated a critical role of the cyclic nucleotide binding domain D (CNB-D) (Kim et al. PLoS Pathog 2015).

**Methods:** *Pf*PKG was recombinantly expressed lacking the putative pseudosubstrate autoinhibitory sequence (IS) and containing only the CNB-D and the catalytic domain. Kinase activity was determined using a coupled spectrophotometric assay. Activation constants (Kact) were determined using a micro-fluidic mobility-shift assay. cGMP-bding was quantified using Surface plasmon resonance (SPR) and fluorescence polarization (FP).

**Results:** We identified a novel mechanism, where the CNB-D controls activation and regulation of the parasite specific protein kinase (Franz et al. ACS Infect Dis. 2018). *Pf*PKG, lacking the IS and containing only the CNB-D is catalytically inactive in the absence of cGMP and be can efficiently activated with cGMP. These observations indicate that kinase regulation is not dependent on an IS, as reported for human PKG. On the basis of structural evidence of the isolated CNB-D (Kim et al. PLoS Pathog 2015) and full length *Pf*PKG (PDB 5DYK), we describe a regulatory mechanism, whereby cGMP binding to the CNB-D induces a conformational change involving the αC-helix of the CNB-D. The inactive state is defined by a unique interaction between Asp597 of the catalytic domain and Arg528 of the αC-helix. The same arginine (Arg528), however, stabilizes cGMP binding by interacting with Tyr480 of the phosphate binding cassette (PBC) in the active state of *Pf*PKG. Consequently, deletion constructs of *h*PKG were generated, lacking the IS and CNB-A. Both, type I and II *h*PKG proteins were constitutively active in the presence or absence of cAMP indicating a different regulatory mechanism than *Pf*PKG, where the IS seems to be required for regulation.

**Conclusions:** Our results unveil fundamental differences in the activation mechanism between *Pf*PKG and *h*PKG, building the basis for the development of strategies for targeted drug design in order to fight malaria.

**Acknowledgement:** F.W.H. is funded by the Deutsche Forschungsgemeinschaft (DFG HE 1818/10).

**References**

- E Franz, MJ Knape, FW Herberg. cGMP binding domain D mediates a unique activation mechanism in Plasmodium falciparum PKG. ACS Infect Dis. 2018;4(3):415–23.
- Kim JJ, Flueck C, Franz E, Sanabria-Figueroa E, Thompson E, Lorenz R, Bertinetti D, Baker DA, Herberg FW, Kim C. Crystal structures of the carboxyl cGMP binding domain of the *Plasmodium falciparum* cGMP-dependent protein kinase reveal a novel capping triad crucial for merozoite egress. PLoS Pathog. 2015;11(2), e1004639.

## S 2-03 “Prison Break”: *Toxoplasma gondii* egress from infected cells is a programmed event governed by a lipid mediator via a guanylate cyclase receptor platform

### Dominique Soldati-Favre, Hugo Bisio, Matteo Lunghi, Mathieu Brochet

#### Universtiy of Geneva, Department Microbiology and Molecular Medicine, Geneva Genève, Switzerland

##### **Correspondence:** Dominique Soldati-Favre - dominique.soldati-favre@unige.ch

*Journal of Translational Medicine* 2019, **17(2):**S 2-03

**Introduction:** The Apicomplexa phylum includes a large group of obligate intracellular protozoan parasites responsible for important diseases in humans and animals. *Toxoplasma gondii* is a widespread parasite with considerable versatility and capable of infecting virtually any warm-blooded animals, including humans. Host cell entry and egress are key steps in the lytic cycle of this obligate intracellular parasite that ensure its survival and dissemination. Egress is temporally orchestrated, underpinned by the exocytosis of secretory organelles called micronemes. At any point during intracellular replication, deleterious environmental changes like loss of host cell integrity can trigger egress (1) via the activation of the cGMP-dependent protein kinase G (PKG) (2).

Intriguingly, we have known for years that even in the absence of alerting, extrinsic signals triggering egress, the number of divisions of the parasite in a single host cell is limited. Egress occurs after 5 to 6 cycles of asexual multiplication. This programmed egress could be due to a mechanical breakage of the host cell plasma membrane containing enlarged vacuole resulting in leak out of potassium and induction of egress. Alternatively, an intrinsic signal produced by the parasite could control and govern the maximum number of cycles before egress.

**Methods:** We combined the genetic tractability of *Toxoplasma gondii* with biological and biochemical approaches to unravel the nature of the intrinsic signal and the associated signaling cascade that govern egress.

**Results:** We could show that diacylglycerol kinase 2 (DGK2) is secreted into the parasitophorous vacuole where it produces phosphatidic acid (PA). PA acts as an intrinsic signal eliciting natural egress upstream of an atypical guanylate cyclase (GC) uniquely conserved in alveolates and ciliates and composed of a P4-ATPase and two GC catalytic domains (3–5). The GC produces cytosolic cGMP that serves as initiator of the entire signalling cascade leading to egress. Assembly of GC at the plasma membrane depends on two associated cofactors, cell division control 50.1 (CDC50.1) and unique GC organizer (UGO) (5).

**Conclusions:** This study reveals the existence of a signaling platform responding to an intrinsic lipid mediator and extrinsic alarming signals to control programmed and induced egress (Fig. 1).

**Fig. 1:**
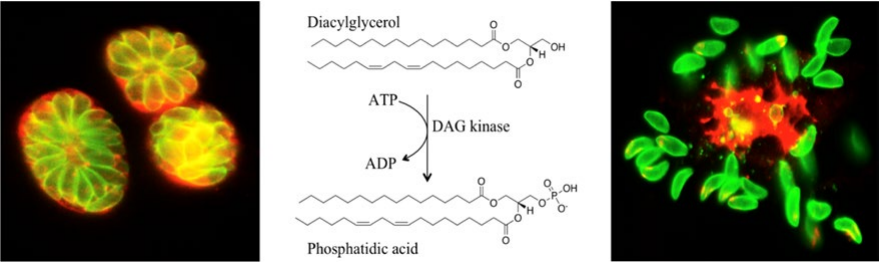
Toxoplasma gondii and Egress. Phosphatidic acid produced by the parasite acts as key lipid mediator in the control of egress from infected cells

**Acknowledgement:** Swiss National Science Fondation

**References**

1. Moudy R, Manning TJ, Beckers CJ. The loss of cytoplasmic potassium upon host cell breakdown triggers egress of *Toxoplasma gondii.* J Biol Chem. 2001;276:41492–501. 10.1074/jbc.m106154200.
2. Brown KM, Long S, Sibley LD. Plasma membrane association by n-acylation governs PKG function in *Toxoplasma gondii*. MBio. 2017;8. 10.1128/mbio.00375-17.
3. Linder JU, Hoffmann T, Kurz U, Schultz JE. A guanylyl cyclase from Paramecium with 22 transmembrane spans. Expression of the catalytic domains and formation of chimeras with the catalytic domains of mammalian adenylyl cyclases. J Biol Chem. 2000;275:11235–40.
4. Brown KM, Sibley LD. Essential cGMP signaling in toxoplasma is initiated by a hybrid P-type ATPase-guanylate cyclase. Cell Host Microbe. 2018;24(6):804–816.e6. 10.1016/j.chom.2018.10.015.
5. Bisio H, Lunghi M, Brochet M, Soldati-Favre D. Phosphatidic acid governs natural egress in T*oxoplasma gondii* via a guanylate cyclase receptor platform. Nat Microbiol. 2019;4(3):420–8. 10.1038/s41564-018-0339-8.

## S 2-05 Oxidation of cysteine 117 stimulates constitutive activation of the type Iα cGMP-dependent protein kinase

### Jessica L. Sheehe^2^, Adrian D. Bonev^2^, Anna M. Schmoker^3^, Bryan A. Ballif ^3^, Mark T. Nelson^2^, Thomas M. Moon^1^, Wolfgang R. Dostmann^2^

#### ^1^University of Arizona, Chemistry and Biochemistry, Tucson Arizona, US; ^2^University of Vermont, Pharmacology, Burlington Vermont, US; ^3^University of Vermont, Biology, Burlington Vermont, US

##### **Correspondence:** Jessica L. Sheehe - jessicamoon@email.arizona.edu

*Journal of Translational Medicine* 2019, **17(2):**S 2-05

**Introduction:** The type I cGMP-dependent protein kinase (PKG I) is an essential regulator of vascular tone. It has been demonstrated that the type Iα isoform can be constitutively activated by oxidizing conditions. However, the amino acid residues implicated in this phenomenon are not fully elucidated. To investigate the molecular basis for this mechanism, we studied the effects of oxidation using recombinant WT, truncated, and mutant constructs of PKG I.

**Methods:** PKG I constructs were expressed in *Sf*9 cells using the Bac-to-Bac system. Mutants of PKG I were generated by site-directed mutagenesis. Constructs were purified by Ni-IMAC, and the N-terminal hexahistide tag was cleaved with TEV protease. H_2_O_2_ dilutions were freshly prepared before experiments, and concentrations were determined by adsorption spectroscopy. Interprotomer disulfide formation was visualized by 4–12% SDS-PAGE. Activities of reduced and oxidized PKG I constructs were determined by an in vitro phosphotransferase assay using a synthetic peptide substrate. PKG-dependent activation of K_Ca_1.1 was measured by inside-out patch clamp using murine vascular smooth muscle cells isolated from anterior and posterior pial and superior cerebellar arteries. Cysteine oxidation states were determined by mass spectrometry.

**Results:** Using an in vitro assay, we observed that oxidation with hydrogen peroxide (H_2_O_2_) resulted in constitutive, cGMP-independent activation of PKG Iα. PKG Iα C42S and a truncation construct that does not contain Cys-42 (Δ53 PKG I) were both constitutively activated by H_2_O_2_. In contrast, oxidation of PKG Iα C117S maintained its cGMP-dependent activation characteristics, although oxidized PKG Iα C195S did not. To corroborate these results, we also tested the effects of our constructs on the PKG Iα-specific substrate, the large conductance potassium channel (K_Ca_1.1). Application of WT PKG Iα activated by either cGMP or H_2_O_2_ increased the open probabilities of the channel. Neither cGMP nor H_2_O_2_ activation of PKG Iα C42S significantly increased channel open probabilities. Moreover, cGMP-stimulated PKG Iα C117S increased K_Ca_1.1 activity, but this effect was not observed under oxidizing conditions. Finally, we observed that PKG Iα C42S caused channel flickers, indicating dramatically altered K_Ca_1.1 channel characteristics compared with channels exposed to WT PKG Iα.

**Conclusions:** Cumulatively, these results indicate that constitutive activation of PKG Iα proceeds through oxidation of Cys-117 and further suggest that the formation of a sulfur acid is necessary for this phenotype.

**Acknowledgement:** We would like to thank Werner Tegge (Helmholtz Centre for Infection Research (HZI), Braunschweig, Germany) for synthesizing the W15 peptide substrate used in the phosphotransferase assays. Mass spectrometry was supported by the Vermont Genetics Network through National Institutes of Health (NIH) Grant 8P20GM103449 from the INBRE program of NIGMS. SDS-PAGE experiments were imaged with support from NIH Grants 5 P30 RR032135 from the COBRE Program of the National Center for Research Resources and 8 P30 GM103498 from NIGMS. We also thank the Hondal Laboratory at the University of Vermont for helpful discussions and use of resources.

**Reference**

This research was originally published in the Journal of Biological Chemistry.

- Sheehe JL, Bonev AD, Schmoker AM, Ballif BA, Nelson MT, Moon TM, Dostmann WR. Oxidation of cysteine 117 stimulates constitutive activation of the type Iα cGMP-dependent protein kinase. J Biol Chem. 2018;293(43):16791–802.

## S 2-06 Crystal structure of PKG Iβ holoenzyme reveals a trans-inhibiting dimer assembly

### Choel Kim^1,2^, Rajesh Sarma^1^, Darren E. Casteel^3^

#### ^1^Baylor College of Medicine, Pharmacology and Chemical Biology, Houston Texas, US; ^2^Baylor College of Medicine, Biochemistry and Molecular Biology, Houston Texas, US; ^3^University of California, San Diego, Medicine, La Jolla California, US

##### **Correspondence:** Choel Kim - ckim@bcm.edu

*Journal of Translational Medicine* 2019, **17(2):**S 2-06

**Introduction:** As the major molecular switch for regulation of smooth muscle/vascular tone and nociception, mammalian cGMP dependent protein kinase I is a promising therapeutic target for hypertensive diseases and chronic pain. The lack of structural information on the PKG holoenzyme has hindered a detailed understanding of its regulation, though the holoenzyme structure for cAMP dependent protein kinase (PKA) suggests plausible models for PKG regulatory (R) and catalytic (C) domain interactions in the inhibited state.

**Methods:** We determined a crystal structure of PKG Iβ holoenzyme complex at 2.3 Å that enables us to visualize the R–C interface of PKG Iβ of the inhibited state for the first time.

**Results:** We crystallized a monomeric PKG Iβ that lacks the dimerization domain, but the asymmetric unit of the crystal contains a twofold symmetric dimer. The interfaces formed between PKG R and C domains are similar to those seen in the PKA Iα holoenzyme with the inhibitor sequence docked to the active site cleft. However, the overall topology unexpectedly reveals that the R domain of each PKG monomer binds the C domain of the other monomer, giving rise to inhibition in trans (Fig. 1).

**Fig. 1:**
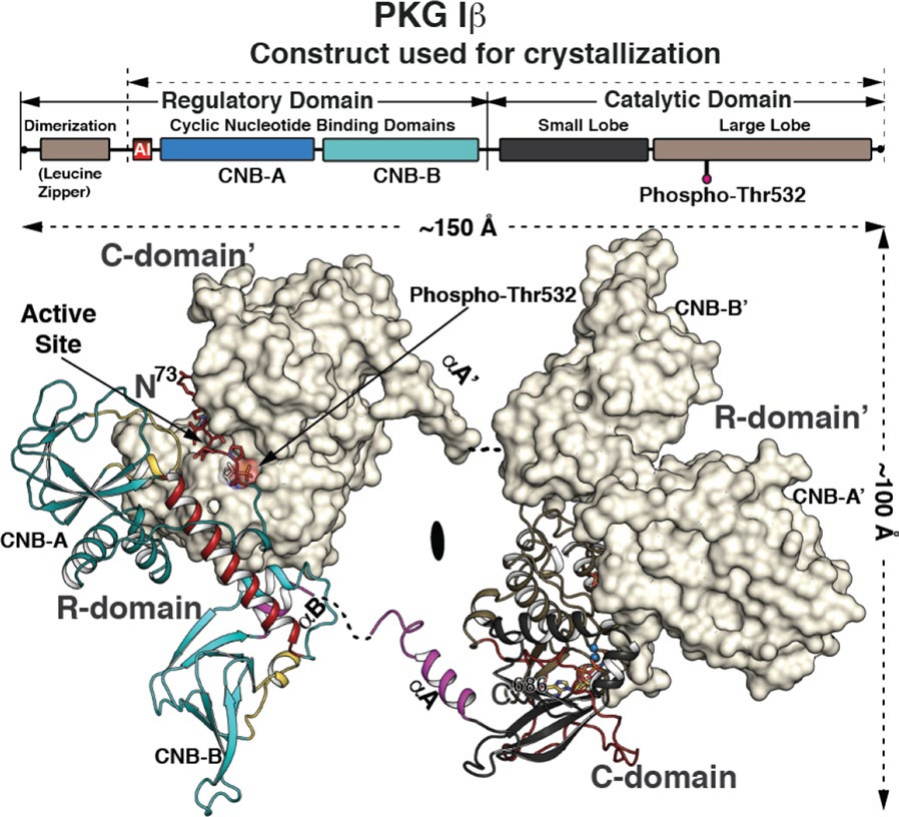
Crystal structure of the PKG Ib holoenzyme complex. The domain organization is shown at the top and the structure of the PKG Ib holoenzyme complex below. The trans-inhibiting dimer is shown with one monomer with surface and the other in cartoon representation. The autoinhibitor (AI) sequence and the interlinking helix between CNB-A and B are colored in red. CNB-A is colored in teal, CNB-B in cyan and PBCs in yellow. The small and large lobes are colored in black and tan respectively. The C-terminal loop is shown in red. The disordered regions between the R and C-domain are shown in dotted lines

**Conclusions:** In the light of previous PKG structural biology, this structure suggests that the PKG inhibited and activated states are stabilized by mutually exclusive domain–domain contacts that either occlude or expose the active site. Our previous structure of the activated state of the isolated PKG Iβ regulatory domain [1] identified a dimer formed by R–R domain interactions (mediated in part by interfacial cGMP). Results from other studies [2–4] suggest how these domain–domain contacts might be differentially stabilized by cyclic nucleotide binding, and how conformational changes associated with nucleotide binding might bias the topology of each full-length monomer towards or away from the trans-inhibited dimer state. Because of sequence differences, PKG lacks some key local contacts that stabilize the PKA holoenzyme R–C interface, perhaps because PKA must overcome mass action to inactivate its catalytic domain, whereas native PKG is already pre-assembled as a dimer. These differences provide starting points for rationally modulating the PKG activation constant by mutagenesis to dissect the details of the mechanism of activation. The quaternary assembly seen in the trans-inhibiting dimer of PKG Iβ differs significantly from other kinases, suggesting a unique regulation mechanism for PKG I with implications for the kinetics, cooperativity, CNB domain nucleotide selectivity, and isotype-specificity of activation.

**Acknowledgement:** This project was supported by National Institutes of Health Grant R01 GM090161 (CK) and by National Institutes of Health Grant RO1 HL132141 (DEC). The expression of PKG Iβ was supported in part by the Protein and Monoclonal Antibody Production Shared Resource at Baylor College of Medicine with funding from National Institutes of Health Cancer Center Support Grant P30 CA125123.

**References**

1. Kim JJ, Lorenz R, Arold ST, Reger AS, Sankaran B, Casteel DE, Herberg FW, Kim C. Crystal structure of PKG I:cGMP complex reveals a cGMP-mediated dimeric interface that facilitates cGMP-induced activation. Structure. 2016;24:710–20.
2. Huang GY, Kim JJ, Reger AS, Lorenz R, Moon EW, Zhao C, Casteel DE, Bertinetti D, Vanschouwen B, Selvaratnam R, Pflugrath JW, Sankaran B, Melacini G, Herberg FW, Kim C. Structural basis for cyclic-nucleotide selectivity and cGMP-selective activation of PKG I. Structure. 2014;22:116–24.
3. Kim JJ, Casteel DE, Huang G, Kwon TH, Ren RK, Zwart P, Headd JJ, Brown NG, Chow DC, Palzkill T, Kim C. Co-crystal structures of PKG Ibeta (92-227) with cGMP and cAMP reveal the molecular details of cyclic-nucleotide binding. PLoS ONE. 2011;6:e18413.
4. Qin L, Sankaran B, Aminzai S, Casteel DE, and Kim C. Structural basis for selective inhibition of human PKG Ialpha by the balanol-like compound N46. J Biol Chem. 2018;293:10985–92.

## S 3-01 Guanylyl cyclase-A activation decreases heart size and adiposity in male mice

### Jerid Robinson^1^, Chastity Healy^2^, Deborah Dickey^1^, Brandon Wagner^2^, Siu-Pok Yee^3^, Laurinda Jaffe^3^, Madeline Gauthier^2^, John Osborn^2^, Timothy O’Connell^2^, Lincoln Potter^1^

#### ^1^University of Minnesota, Department of Biochemistry, Minneapolis Minnesota, US; ^2^University of Minnesota, Department of Physiology, Minneapolis Minnesota, US; ^3^University of Connecticut, Department of Cell Biology, Farmington Connecticut, US

##### **Correspondence:** Lincoln Potter - potter@umn.edu

*Journal of Translational Medicine* 2019, **17(2):**S 3-01

**Introduction:** Atrial natriuretic peptide (NP) and B-type NP reduce blood pressure and cardiac hypertrophy by activating guanylyl cyclase (GC)-A, a transmembrane, cGMP-synthesizing receptor (1). GC-A is basally phosphorylated on multiple serines and threonines and dephosphorylation renders the enzyme unresponsive to NP stimulation. We generated a knock-in mouse called GC-A^8E/8E^ where glutamates were substituted for the eight conserved phosphorylation sites in GC-A to mimic a constitutively phosphorylated enzyme that is activated normally by NPs but cannot be inactivated by dephosphorylation to determine which processes are regulated by changes in GC-A phosphorylation (2).

**Methods:** CRISPR–CAS9 technology was used to generate the GC-A^8E/8E^ mice. Wild type and GC-A^8E/8E^ mice were implanted with Data Science International Inc. (St. Paul, MN) transmitter (model HD-X11) for continuous monitoring of arterial pressure and heart rate. Hearts from 12 to 13-week-old male and female wild type and mutant mice were removed and weighed. Heart apexes were embedded in paraffin blocks on cassettes such that the sections would be transverse at the midpoint of the ventricles. Male 12–13 week old GC-A^WT/WT^ and GC-A^8E/8E^ mice were assessed for body composition by EchoMRI.

**Results:** ANP-dependent guanylyl cyclase activities were elevated two- to fourfold in heart and kidney membranes from the GC-A^8E/8E^ mice with no changes in GC-A protein levels. Blood pressure measured in response to low salt, normal salt, high salt, or dehydration did not differ between the mutant and wild type mice. In contrast, the hearts of the male, but not female, mutant mice were 12% smaller than hearts from wild type animals, which was explained in part by a decrease in cardiomyocyte area. Male 12–13 week old GC-A^WT/WT^ and GC-A^8E/8E^ mice were also assessed for body composition by EchoMRI. Male GC-A^8E/8E^ mice have a 30% reduction in percent fat mass compared to GC-A^WT/WT^ mice, whereas, lean mass and free water mass was unchanged between the wild type and mutant mice. The male GC-A^8E/8E^ mice also had less total mass than wild type mice (Fig. 8D), which was accounted for by the decreased fat mass. Finally, serum testosterone levels were 25% higher in the mutant compared to the wild type mice.

**Conclusions:** Increased GC-A activity results in decreased heart size, increased adiposity and increase testosterone levels in male mice. The contribution of the increased testosterone levels to the heart and adipose effects is under investigation.

**Acknowledgement:** This work was supported by National Institutes of Health Grant R01GM098309, a University of Minnesota Foundation Bridge Grant to LRP, a University of Minnesota-Mayo Clinic Partnership grant, a University of Minnesota Academic Health Center Faculty Research and Development Grant to LRP as well as an NIHT32DK007203 Grant to JWR. Grants from the Fund for Science and the Hormone Receptor Fund to LRP.

**References**

- Kuhn M. molecular physiology of membrane guanylyl cyclase receptors. Physiol Rev. 2016;96(2):751–804.
- Otto NM, McDowell WG, Dickey DM, Potter LR. A Glutamate-substituted mutant mimics the phosphorylated and active form of guanylyl cyclase-A. Mol Pharmacol. 2017;92(1):67–74. **(Epub 2017/04/17)**.

## S 3-02 Paracrine cyclic GMP-mediated anti-inflammatory actions of C-type natriuretic peptide

### Michaela Kuhn, Wen Chen

#### Institute of Physiology, University of Wuerzburg, Wuerzburg, Germany

##### **Correspondence:** Michaela Kuhn - michaela.kuhn@mail.uni-wuerzburg.de

*Journal of Translational Medicine* 2019, **17(2):**S 3-02

**Introduction:** C-type “natriuretic” peptide (CNP) and its guanylyl cyclase-B (GC-B) receptor form a local signaling pathway in the bone growth plate which is essential for endochondral bone growth [1, 2]. In addition, CNP is expressed in different types of cardiovascular cells, i.e. in vascular endothelium. It was shown that *exogenous*, synthetic CNP exerts antiinflammatory effects in different disease model, suggesting that the *endogenous* hormone may support vascular barrier functions [1, 3]. Indeed, in mice disruption of endothelial CNP provoked endothelial dysfunction and enhanced leucocyte rolling and extravasation [4]. Intriguingly, these studies indicated that natriuretic peptide receptor-C (NPR-C, the NP “clearance” receptor) and cyclic GMP-independent, G-protein coupled intracellular pathways mediate such vascular barrier protecting actions of CNP [4]. However, our own together with published studies show that the cyclic GMP-producing GC-B receptor for CNP is co-expressed with the NPR-C receptors in macro- and microvascular endothelial cells and in different types of immune cells [5].

**Methods:** To dissect the possible local (peri)vascular, i.e. antiinflammatory functions of the endogenous CNP/GC-B/cyclic GMP signaling pathway we generated novel genetic mouse models with conditional, deletions of the GC-B receptor in different cells involved in inflammatory processes, and characterized the impact on vascular barrier functions in physiology and disease.

**Results:** Our observations strengthen the concept that endothelial CNP via GC-B/cGMP signalling stabilizes specific types of immune cells at baseline and prevents their excessive activation in response to stressors such as ischemia.

**Conclusions:** CNP, via GC-B/cGMP signaling, contributes to the physiological maintenance of vascular integrity and attenuates ischemic tissue damage and thromboinflammation.

**Acknowledgement:** These studies are supported by the Deutsche Forschungsgemeinschaft (SFB TR166, DFG KU1036 7-1 and 8-1) and the Bundesministerium für Forschung und Technik (BMBF 01 EO1004).

**References**

1. Kuhn M. Molecular physiology of membrane guanylyl cyclase receptors. Physiol Rev. 2016;96:751–804.
2. Nakao K, Osawa K, Yasoda A, Yamanaka S, Fujii T, Kondo E, Koyama N, Kanamoto N, Miura M, Kuwahara K, Akiyama H, Bessho K, Nakao K. The local CNP/GC-B system in growth plate is responsible for physiological endochondral bone growth. Sci Rep. 2015;5:10554.
3. Bae CR, Hino J, Hosoda H, Arai Y, Son C, Makino H, Tokudome T, Tomita T, Kimura T, Nojiri T, Hosoda K, Miyazato M, Kangawa K. Overexpression of C-type natriuretic peptide in endothelial cells protects against insulin resistance and inflammation during diet-induced obesity. Sci Rep. 2017;7:9807.
4. AJ Moyes, RS Khambata, I Villar, KJ Bubb, et al., A Ahluwalia, AJ Hobbs. Endothelial C-type natriuretic peptide maintains vascular homeostasis. J Clin Invest. 2014;124:4039–51.
5. Špiranec K, Chen W, Werner F, Nikolaev VO, Naruke T, Koch F, Werner A, Eder-Negrin P, Diéguez-Hurtado R, Adams RH, Baba HA, Schmidt H, Schuh K, Skryabin BV, Movahedi K, Schweda F, Kuhn M. Endothelial C-type natriuretic peptide acts on pericytes to regulate microcirculatory flow and blood pressure. Circulation. 2018;138:494–508.

## S 3-03 cGMP microdomains regulate natriuretic receptor signalling in cardiac myocytes

### Viacheslav O. Nikolaev

#### University Medical Center Hamburg-Eppendorf, Institute of Experimental Cardiovascular Research, Hamburg, Germany

##### **Correspondence:** Viacheslav O. Nikolaev - v.nikolaev@uke.de

*Journal of Translational Medicine* 2019, **17(2):**S 3-03

**Introduction:** 3′,5′-cyclic guanosine monophosphate (cGMP) is an ubiquitous second messenger that regulates cardiac function by acting in distinct subcellular microdomains. Some functionally important microdomains are formed for example around calcium handling proteins. Local cGMP dynamics is usually regulated by anchored pools of kinases and cyclic nucleotide degrading enzymes phosphodiesterases (PDEs). However, such compartmentalised cGMP signals have not been extensively studied before. Over the last years, we developed cytosolic and targeted Förster resonance energy transfer (FRET) based biosensors expressed in transgenic mice to monitor cytosolic and microdomains-specific cGMP.

**Methods:** We developed cytosolic and targeted Förster resonance energy transfer (FRET) based biosensors expressed in transgenic mice to monitor cytosolic and microdomains-specific cGMP. Using scanning ion conductance microscopy (SICM) combined with FRET, we could determine exact membrane localisation of natriuretic peptide receptors guanylyl cyclases A and B (GC-A and GC-B) and to analyse subcellular compartmentation of their cGMP signals.

**Results:** Whereas GC-B (receptor for C-type natriuretic peptide, CNP) is uniformly localised on the cardiomyocyte membrane, functional GC-A receptors (for atrial natriuretic peptide, ANP) are found exclusively in membrane invaginations called transverse (T)-tubules. Although both ANP and CNP protect from cardiac hypertrophy, their effects on contractility are markedly different, from almost no effect (ANP) to strong negative inotropic and positive lusitropic responses (CNP) with unclear underlying mechanisms. Using out real time imaging techniques, we could show that uniform CNP membrane localisation leads to CNP/GC-B/cGMP signals which diffuse over long distances inside the cell, whereas ANP/GC-A/cGMP signals are highly confined to T-tubular microdomains by local pools of PDE2 activity.

**Conclusions:** This provides a clear molecular basis and explains distinct functional effects engaged by the two natriuretic peptide receptors in the heart.

## S 3-04 NP/NO regulation of cardiac sympathetic drive

### David J. Paterson

#### University of Oxford, Department of Physiology, Anatomy & Genetics, Oxford, UK

##### **Correspondence:** David J. Paterson - david.paterson@dpag.ox.ac.uk

*Journal of Translational Medicine* 2019, **17(2):**S 3-04

**Introduction:** Emerging evidence suggests that impairment of nitric oxide (NO) and naturietic peptide activation of cyclic guanosine monophosphate (cGMP) disrupts intracellular calcium homeostasis and increases cardiac sympathetic neurotransmission. We tested whether phosphodiesterase 2A (PDE2A), which regulates the action of BNP-activated cGMP, was directly involved in modulating Ca^2+^ handling from stellate ganglia (SG) neurons and cardiac norepinephrine (NE) release in rats and humans with an enhanced sympathetic phenotype. We also tested whether gene knockdown of PDE2A or overexpression of neuronal NO could rescue the sympathetic phenotype.

**Methods:** Stellate ganglia (SG) were harvested from patients undergoing stellectomy for sympathetic hyperactivity or from donor patients for molecular profiling (Western analysis and qRT-PCR). In rats predisposed to hypertension and their controls, stellate ganglia were also isolated for bulk and single cell RNAseq. An adenoviral vector expressing PDE2A or catalytically inactive PDE2A (dnPDE2A) tagged with red fluorescent protein mCherry was transduced into cultured rat cardiac sympathetic neurons (for calcium current and transient measurements). An adenoviral vector expressing only mCherry was used as a control for comparing the effect of viral transduction following application of BNP. cGMP, cAMP and PKA were measured using FRET sensors (Gi500, pfPKG, H187 AKAR). A viral vector encoding a noradrenergic promotor for nNOS or its adaptor protein was given in vivo in diseased and normal rats to establish if neurotransmission could be normalised.

**Results:** PDE2A was upregulated in stellate ganglia from both human and rat diseased ganglia. Single cell seq also revealed the presence of PDE2A in *Th* positive neurons. nNOS, sGC and cGMP was decreased in stellate ganglia from pre-hypertensive rats. This was associated with increased conductance of I_CaN_ (Cav 2.2), increased calcium transients and neurotransmission. Diseased neurons also had a blunted response to cGMP to increasing concentrations of BNP. Similarly overexpression of PDE2A in healthy neurons recaptulated the diseased cellular phenotype. Inhibition of PDE2A with BAY 60-7550 or dnPDE2A restored calcium balance and neurotransmission in diseased neurons. Gene transfer of nNOS or the adaptor protein to nNOS (CAPON) also rescued abnormal transmission in diseased neurons.

**Conclusions:** These results suggest that impaired second messenger signalling coupled to dysregulation of PDE2A is linked to abnormal sympathetic hyperactivity in states associated with hypertension and heart failure. Site specific targeting of the cyclic nucleotide-PDE2A pathway may provide therapeutic utility.

**Acknowledgement:** This work was supported by a BHF Programme grant.

**References**

- Herring N, Kalla M, Paterson DJ. The nervous system and cardiac arrhythmias: current concepts and emerging therapies. Nature Revs Cardiol. 2019. **(In press)**.
- Liu K, Li D, Hao G, McCaffary D, Neely O, Woodward L, Ioannides D, Lu CJ, Brescia M, Zaccolo M, Tandri H, Ajijola OA, Ardell JL, Shivkumar K, Paterson DJ. Phosphodiesterase 2A as a therapeutic target to restore cardiac neurotransmission during sympathetic hyperactivity. JCI Insight. . 2018. 10.1172/jci.insight.98694.
- Li D, Lu C-J, Hao G, Wright H, Woodward L, Liu K, Vergari E, Surdo NC, Herring N, Zaccolo M, Paterson DJ. Efficacy of B type natriuretic peptide is coupled to phosphodiesterase 2A in cardiac sympathetic neurons. Hypertension. 2015;66(1):190–8.

## S 3-05 Sacubitril/valsartan improves LV function in chronic pressure overload independently of intact cGMP-dependent protein kinase I alpha signaling

### Kelly Tam, Daniel A. Richards, Mark J. Aronovitz, Gregory L. Martin, Iris Z. Jaffe, Robert M. Blanton

#### Tufts Medical Center, Molecular Cardiology Research Institute, Boston Massachusetts, US

##### **Correspondence:** Robert M. Blanton - rblanton@tuftsmedicalcenter.org

*Journal of Translational Medicine* 2019, **17(2):**S 3-05

**Introduction:** Combined angiotensin receptor/neprilysin inhibition with sacubitril/valsartan (Sac/Val) has recently been approved as a novel therapeutic strategy in heart failure (HF) [1]. Neprilysin degrades multiple circulating peptides, but the presumed mechanism of benefit of sacubitril occurs via prevention of natriuretic peptide (NP) degradation, leading to augmentation of cGMP-dependent protein kinase (PKG) signaling. The potential additive effects of sacubitril on top of angiotensin receptor blocker blockade with valsartan remain poorly understood. Further, the specific requirement of PKG for the cardiovascular effects of Sac/Val in presure overload are unknown. We previously demonstrated that mutation of the PKGI alpha leucine zipper protein interaction domain blocks the anti-remodeling effects of the cGMP augmenting drug sildenafil in left ventricular (LV) pressure overload [2]. The current study tested the hypothesis that the PKGIα LZ domain also mediates the anti-remodeling effects of Sac/Val in LV pressure overload.

**Methods:** We subjected 12 week old wild type (WT) or PKG Leucine Zipper Mutant (LZM) mice to 56 day LV pressure overload by moderate (26G) transaortic constriction (TAC) or to sham surgery. At 14 days post-TAC, mice were randomized mice to vehicle (Veh), valsartan (Val, 52 mg/kg/day), and Sac/Val (50 mg/kg/day Sac, 52 mg/kg/day Val) by gavage. At day 56, echocardiography, LV invasive hemodynamics, and organ harvest were performed.

**Results:** Compared with WT mice, LZM TAC mice developed worse LV fractional shortening (FS) from day 14 to day 56, as well as increases in LV end diastolic dimension, indicating more severe response to TAC. In WT TAC mice, both Val and Sac/Val reduced LV hypertrophy to the same degree. However, in WT mice only Sac/Val, but not Val, improved LV FS compared with vehicle, indicating an additive effect of sacubitril to valsartan alone. In LZM TAC mice, Sac/Val also improved LV FS, whereas Val alone did not improve this parameter. Sac/Val, but not Val, also improved invasive measures of LV systolic function in LZM TAC mice, compared with vehicle or with Val. When compared by genotype, Sac/Val induced identical improvements in LV end systolic diameter and in LV FS between WT and LZM mice.

**Conclusions:** These findings indicate a selective effect of Sac/Val versus Val alone on LV function in moderate pressure overload. Mutation of the PKGIα LZ domain induces a more severe TAC phenotype but does not abolish the effect of Sac/Val on LV function. These findings support that the PKGIα LZ domain does not mediate effects of Sac/Val in TAC, and suggest that signaling other than the NP-cGMP-PKGIa axis may mediate the benefit of neprilysin inhibition in HF (Fig. 1).

**Fig. 1:**
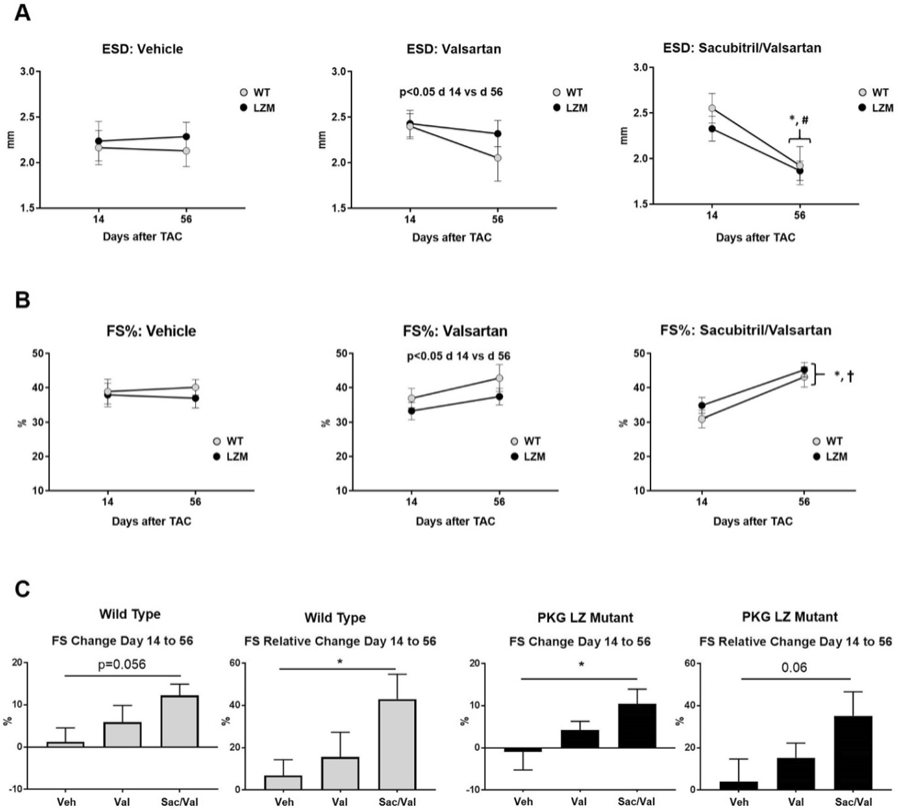
Similar effects of sacubitril/valsartan on LV function in wild type and in PKGIa LZ mutant mice. **a** End systolic diameter (ESD) on Day 14 (start of drug) or Day 56 (final day of drug) in WT and PKG LZ mutant mice treated with vehicle, valsartan, or sacubitril/valsartan. **b** Fractional shortening (FS%) on Day 14 or Day 56 in WT and PKG LZ mutant mice treated with vehicle, valsartan, or sacubitri/valsartan. *p < 0.01 WT day 56 vs WT day 14; ^#^p = 0.06 LZM day 56 vs LZM day 14; ^†^p < 0.01 LZM day 56 vs LZM day 14. **c** Summary data for absolute and relative change in LV fractional shortening from day 14 to day 56. *p < 0.05. For each treatment n = 8 WT, 10–11, LZM

**Acknowledgement:** This study was funded by Novartis Pharmaceuticals through an investigator initiated preclinical study award (LCZ696BUSNC19T) to RMB and IZJ, and by the NIH R01HL131831 to RMB.

**References**

1. McMurray JJ, Packer M, Desai AS, Gong J, Lefkowitz MP, Rizkala AR, Rouleau JL, Shi VC, Solomon SD, Swedberg K, Zile MR. Angiotensin-neprilysin inhibition versus enalapril in heart failure. N Engl J Med. 2014;371:993–1004.
2. Blanton RM, Takimoto E, Lane AM, Aronovitz M, Piotrowski R, Karas RH, Kass DA, Mendelsohn ME. Protein kinase g iα inhibits pressure overload-induced cardiac remodeling and is required for the cardioprotective effect of sildenafil in vivo. J Am Heart Assoc. 2012;1:e003731.

## S 4-01 A role of the guanylyl cyclase GC-A in auditory function

### Lukas Rüttiger^1^, Dorit Möhrle^1^, Markus Wolters^2^, Philipp Eckert^1^, Philine Marchetta^1^, Steffen Wolter^1^, Katrin Reimann^1^, Frank Schweda^3^, Michaela Kuhn^4^, Jutta Engel^5^, Marlies Knipper^1^

#### ^1^University of Tübingen, Department of Otolaryngology Head&Neck Surgery, Tübingen, Germany; ^2^University of Tübingen, Interfaculty Institute of Biochemistry, Tübingen, Germany; ^3^University of Regensburg, Institute of Physiology, Regensburg, Germany; ^4^University of Würzburg, Department of Physiology, Würzburg, Germany; ^5^Saarland University, Department of Biophysics, Homburg, Germany

##### **Correspondence:** Lukas Rüttiger - lukas.ruettiger@uni-tuebingen.de

*Journal of Translational Medicine* 2019, **17(2):**S 4-01

**Introduction:** In the inner ear, the cGMP signaling pathway has been described to facilitate both protective and harmful processes in response to traumatic events. The aim of this study was to investigate the otoprotective role of the particulate cGMP generator guanylyl cyclase type A (GC-A) and its ligands atrial and B-type natriuretic peptides (ANP, BNP) for auditory function.

**Methods:** Transgenic mice knockout (KO) for GC-A were tested for hearing function and for auditory recovery from noise injury. We studied GC-A, ANP and BNP expression in the hearing organ, the inner ear (cochlea) with the organ of corti containing hair cells for mechano-electrical transduction of sound information. We determined the hearing function of the mice from auditory brainstem responses (ABRs) and distortion-product otoacoustic emissions (DPOAEs).

**Results:** Our results show that GC-A is expressed in outer hair cells (OHCs), while ANP and BNP are expressed in OHCs and sensory inner hair cells (IHCs). We analyzed the hearing function of GC-A KO animals before and after auditory trauma from loud sound exposure. In the mouse, GC-A deficiency leads to age related, progressive hearing impairment. Already at young age, GC-A deficiency increases the vulnerability of our hearing organ to loud sound exposure.

**Conclusions:** A role of cGMP within the ANP and BNP/GC-A/cGMP signaling pathway in the inner ear is considered for age and noise induced hearing loss (Fig. 1).

**Fig. 1:**
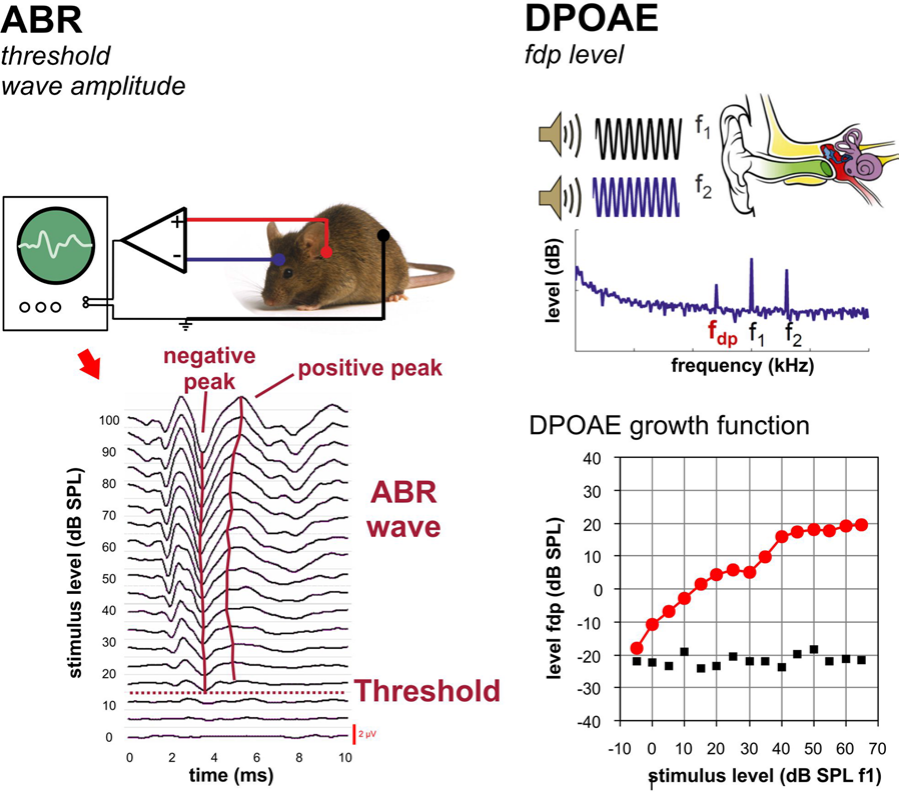
Methods to determine the hearing function in mice. *ABR* auditory brainstem responses. Auditory evoked summation potentials are recorded from the scalp. The potentials to increasing sound stimulation contain characteristic deflections (waves). Wave thresholds depict the hearing sensitivity. Wave amplitudes and latencies depict the capacitiy of distinct stages within the ascending auditory pathway to process auditory information. *DPOAE* distortion products of the otoacoustic emissions. An acoustic measure to determine the function of the outer hair cells. Both procedures are also regularly used in the clinic for human hearing diagnostic

**Acknowledgement:** This work was supported by grants from the Deutsche Forschungsgemeinschaft (FOR 2060 project FE 438/6-1, RU 713/3-2, DFG-Kni-316-10-1, SPP 1608 RU 316/12-1, and SPP 1608 KN 316/12-1).

## S 4-02 Role of natriuretic peptide receptors in pulmonary vascular endothelial function

### James R. Klinger^1,2^, Ashok Kumar^2^, Julie Braza^2^, Huetran Duong^2^, Michaela Kuhn^3^, Elizabeth O. Harrington^1,2^

#### ^1^Rhode Island Hospital Brown University, Division of Pulmonary and Critical Care Medicine, Providence Rhode Island, US; ^2^Providence Veterans Administration Medical Center, Vascular Research Lab, Providence Rhode Island, US; ^3^Universität Würzburg, Physiologisches Institut I, Würzburg, Germany

##### **Correspondence:** James R. Klinger - james_klinger@brown.edu

*Journal of Translational Medicine* 2019, **17(2):**S 4-02

**Introduction:** Atrial natriuretic peptide (ANP) protects against acute lung injury, but the receptor(s) that mediate this effect is not known. We examined the effect of genetic deletion of particulate guanylyl cyclase-linked (GC-A) and non guanylyl cyclase-linked receptor (NPR-C) on the ability of ANP to mitigate acute lung injury.

**Methods:** Endothelial specific GC-A knockout mice (GC-A(−/−))were created by homologous loxP/Tie2-Cre-mediated recombination. Global, NPR-C knock out mice (NPRC(−/−)) were obtained from Jackson Labs (Bar Harbor, ME). Acute lung injury was induced by oral pharyngeal aspiration of pseudomonas aeruginosa-103 (PA103) 30 min after continuous intravenous infusion of ANP (180 ng/kg/min) for 150 min in 1.0 ml of normal saline or normal saline alone. Lung injury was assessed at 4 h by measurement of lung wet to dry weight (W/D) and protein and cell content of bronchoalveolar lavage fluid (BALF).

**Results:** In NPR-C wild-type mice (NPRC(+/+)), PA103 increased lung W/D (6.09 ± 0.52 vs 4.30 ± 0.19) and BALF protein (3.05 ± 0.65 vs 0.54 ± 0.16 mg/ml) compared to mice that aspirated saline (P < 0.05 for both). ANP + PA103 decreased lung W/D (5.36 ± 0.49 vs 6.09 ± 0.52) and BALF protein (1.26 ± 0.47 vs 3.05 ± 0.65 mg/ml) compared to mice treated with saline + PA103 (P < 0.05 for both). Similarly, PA103 caused significant increases in lung W/D (6.21 ± 0.28 vs 4.12 0.40) and BALF protein (3.39 ± 0.64 vs 0.60 ± 0.09 mg/ml) in NPRC(−/−) and ANP was just as effective at blunting PA103-induced increases in lung W/D and BALF protein (5.22 ± 0.61 vs 6.21 ± 0.28 and 1.61 ± 0.43 vs 3.39 ± 0.6 mg/ml, P < 0.05 for both). In GC-A wild-type mice (GC-A(+/+)), PA103 increased lung W/D (6.52 ± 0.25 vs 4.29 ± 0.13) and BALF protein (2.16 ± 0.38 vs 0.25 ± 0.11 mg/ml) compared to saline (P < 0.05 for both). Lung W/D ratio (4.59 ± 0.28 vs 6.52 ± 0.25) and BALF protein (0.48 ± 0.38 vs 2.16 ± 0.38 mg/ml) were lower in GC-A(+/+) mice treated with ANP + PA103 than in mice given Saline + PA103 (P < 0.05 for both). Similarly, PA103 increased lung W/D (6.09 ± 0.44 vs 4.59 ± 0.16) and BALF protein (1.82 ± 0.94 vs 0.31 ± 0.15 mg/ml) in GC-A(−/−) mice. However, there was no difference in lung W/D (6.10 ± 0.17 vs 6.09 ± 0.44) or BALF protein (1.55 ± 0.1.47 vs 1.82 ± 0.94 mg/ml) between mice given ANP + PA103 vs saline + PA103. Neither PA103 nor ANP had any significant effect on BALF cell counts. Overexpression of GC-A, but not NPR-C in pulmonary vascular endothelial cells enhanced the protective effect of ANP against TNFα-induced increases in permeability in vitro.

**Conclusions:** GC-A mediates the protective effect of ANP on pulmonary vascular endothelial barrier function and may represent a new potential target for treatment of acute lung injury (Fig. 1).

**Fig. 1:**
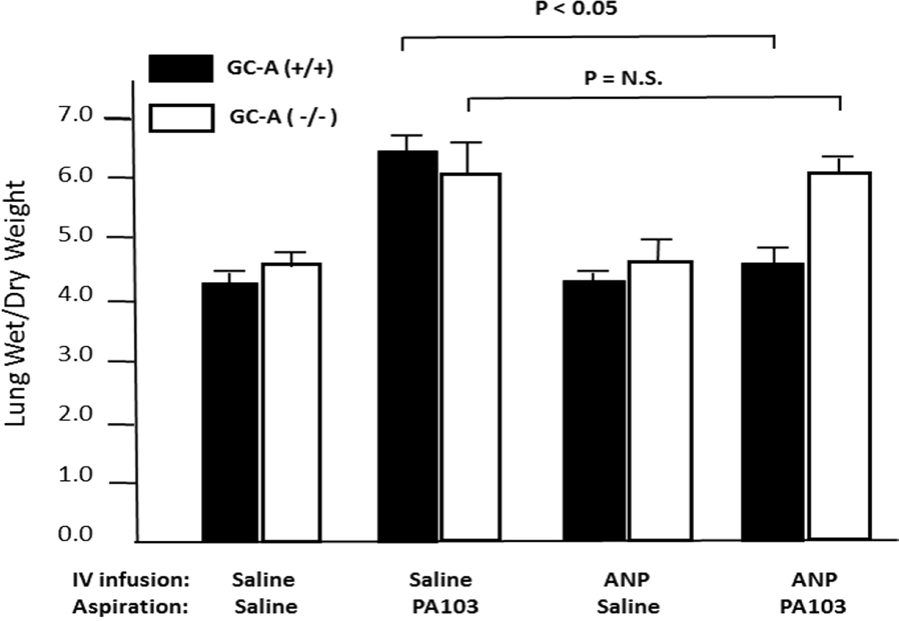
ANP fails to inhibit acute lung injury in GC-A knockout mice. Guanylyl cyclase-A wild-type (GC-A+/+) and knockout mice (GC-A−/−) mice were given an intravenous infusion of ANP or saline alone starting 30 min prior to oral pharyngeal aspiration of saline or psuedomonas 103 (PA103). Animals were sacrificed and lung weight to dry weight ratio was measured 4 h later. PA103 cause a marked increase in pulmonary edema as assessed by lung wet to dry weight in both genotypes. This effect was completely blocked by ANP infusion in wild-type mice, but ANP had no effect in GC-A knockout mice. Data are mean ± standard deviation, n = 4–6 animals per group.

## S 4-03 Mutation in cGMP-dependent protein kinase isoform Iα is associated with aortic root dilation and aneurism formation in patients, potentially due to altered kinase activity and cGMP binding kinetics

### Philipp Henning^1^, Friederike Cuello^2,3^, Friedrich W. Herberg^1^

#### ^1^University of Kassel, Department of Biochemistry, Kassel Hesse, Germany; ^2^University Medical Center Hamburg-Eppendorf, Department of Experimental Pharmacology and Toxicology, Hamburg, Germany; ^3^DZHK (German Center for Cardiovascular Research), partner site Hamburg/Kiel/Lübeck, Hamburg, Germany

##### **Correspondence:** Philipp Henning - p.henning@uni-kassel.de

*Journal of Translational Medicine* 2019, **17(2):**S 4-03

**Introduction:** The cGMP-dependent protein kinase isoform Iα (PKG Iα) is one of the main signal transducers of the nitric oxide/cGMP signalling pathway and plays a crucial role in the cardiovascular system. Malfunctioning of the kinase has severe consequences and results for example in elevated blood pressure and abnormalities in smooth muscle contraction. Recently, a missense variant of PKG Iα with replacement of valine at position 219 by isoleucine (V219I) was identified in a patient with congenital heart disease presenting with aortic aneurism and dilation of the aortic root.

**Methods:** Full-length human PKG Iα wildtype and V219I mutant (*h*PKG Iα WT/V219I) were recombinantly expressed in HEK 293T cells and purified using Strep-Tactin superflow agarose. Kinase activity and activation were measured by an enzyme-coupled photometric assay according to Cook et al. (1982). Surface plasmon resonance (SPR) direct binding assays were performed with *h*PKG Iα WT and V219I as ligand and cGMP as analyte. Both proteins were coupled to the chip surface via Strep-Tactin XT.

**Results:** In order to establish a cause and effect relationship between the mutation in PKG Iα and the human disease phenotype, this study investigated the biochemical characteristics and molecular pathomechanisms of the mutation. Therefore, the full-length mutant protein *h*PKG Iα V219I was recombinantly expressed and biochemically analysed. The activation and kinase activity as well as the binding kinetics of cGMP were monitored in comparison to the wildtype (WT) protein. While our studies demonstrate that the catalytic activity of the mutant protein does not differ from WT, it could be shown that the mutation impacts on the activation constants and the cGMP binding kinetics. The activation constants (*K*_act_) were determined by an enzyme-coupled photometric assay using three different peptide substrates (PKStide, Kemptide and VASPtide). For all three, the *K*_act_ of *h*PKG Iα V219I was shifted to roughly 50% lower cGMP concentrations. Furthermore, cGMP affinities were determined using SPR direct binding assays. These results supported the activation data, with *h*PKG Iα V219I showing a higher affinity to cGMP, increased by a factor of approximately two. The mentioned changes in PKG Iα activation and cGMP binding affinity indicate a higher cGMP sensitivity of the mutant protein.

**Conclusions:** Using different in vitro approaches, we could demonstrate that the *h*PKG Ia V219I mutation displays a higher cGMP sensitivity. In a cellular context, higher basal kinase activity due to increased cGMP affinity might help to explain the human disease phenotype, considering that PKG Ia is an important regulator of vasotone.

**Acknowledgement:** F.W.H. is funded by the Deutsche Forschungsgemeinschaft (DFG HE 1818/10). FC was funded by DFG CU 53/5-1 and the DZHK.

**Reference**

- Cook PF, Neville ME, Vrana KE, Hartl FT, Roskoski R. Adenosine cyclic 3′,5′-monophosphate dependent protein kinase: kinetic mechanism for the bovine skeletal muscle catalytic subunit. Biochemistry. 1982;21(23):5794–9.

## S 4-04 Soluble guanylate cyclase stimulation ameliorates skeletal and cardiac muscle dysfunction in an mdx model of Duchenne muscular dystrophy

### Justin Percival

#### University of Miami Miller School of Medicine, Molecular and Cellular Pharmacology, Miami Florida, US

##### **Correspondence:** Justin Percival - j.percival@med.miami.edu

*Journal of Translational Medicine* 2019, **17(2):**S 4-04

**Introduction:** Mutations in the dystrophin gene cause Duchenne muscular dystrophy (DMD), the most common muscle wasting disease. Loss of dystrophin compromises neuronal nitric oxide synthase (nNOS) signaling, which contributes to skeletal and cardiac muscle dysfunction. Approaches that enhance or restore nNOS signaling reduce dystrophic pathology in murine mdx models of DMD; therefore, enhancing nNOS signaling has potential as a stand-alone therapy or as an adjunct therapy to improve the efficacy of genetic approaches that do not restore nNOS. However, we currently lack an effective pharmacological approach to increase nNOS signaling in muscle. Soluble guanylate cyclase (sGC) is an important target and effector of nNOS in skeletal muscle. Allosteric sGC agonists called sGC stimulators are under development as a novel approach for increasing NO-cGMP signaling and are currently used clinically to treat pulmonary hypertension. Therefore, we hypothesized that sGC stimulation would ameliorate skeletal and cardiac muscle pathology in mdx mice.

**Methods:** To test our hypothesis, we randomly assigned young or aged mdx mice to vehicle or sGC stimulator treatment for 2–16 weeks and analyzed the impact of sGC stimulation on dystrophic skeletal and cardiac muscle pathology.

**Results:** In the skeletal muscles of mdx mice, sGC stimulation promoted muscle cell growth, as well as reduced muscle damage and susceptibility to lengthening contraction-induced injury and post-exercise fatigue. In addition, sGC stimulation increased thoracic diaphragm movement and reduced some breathing irregularities. In aged cardiac muscle, sGC stimulation opposed hypertrophic left ventricle remodeling and indices of diastolic dysfunction. Mechanistically, reductions in skeletal muscle pathology were associated with substantial decreases in macrophage infiltration and pro-inflammatory cytokines. sGC stimulation also decreased fibrosis, and this anti-fibrotic effect was most prominent in cardiac muscle. Improved muscle integrity and function was not due to increased utrophin expression.

**Conclusions:** In summary, these data provide pre-clinical proof-of-concept that sGC stimulation ameliorates skeletal and cardiac muscle pathology in mdx mice. In addition, these data provide important new insights into the role of NO-cGMP signaling in dystrophic pathogenesis that have important implications for the therapeutic potential of cGMP-targeted therapies for DMD. This new understanding provides a compelling rationale for the clinical testing of sGC stimulators as a novel therapy for DMD.

## S 5-01 Designing an allosteric switch: lessons learned from PKA

### Susan S. Taylor, Jason del Rio, Kristofer Haushalter, Tsan-Wen Lu

#### University of California, San Diego, Pharmacology and Chemistry and Biochemisty, La Jolla California, US

##### **Correspondence:** Susan S. Taylor - staylor@ucsd.edu

*Journal of Translational Medicine* 2019, **17(2):**S 5-01

**Introduction:** PKA and PKG have evolved to be molecular switches that use second messengers, cAMP and cGMP, to respond to extracellular queues and then translate those signals into a biological response. While they are close homologs that share a common domain organization and conserved cyclic nucleotide binding domains and are both allosterically regulated by cyclic nucleotides, they also have fundamental differences. PKG, like most other protein kinases, has the regulatory and catalytic domains on a single protomer while PKA is unique in having a “split protomer”. Having separate regulatory (R) and catalytic (C) subunits each having multiple genes and/or splice variants provides a wide range of combinatorial diversity. In addition, there are many levels of cross talk between heterologous kinases including PKA and PKG. While we have learned a great deal from single R and C subunits in PKA, it is not until one sees the R_2_C_2_ holoenzymes that one can appreciate the remarkable full range of allosteric activation as well as the structural diversity of the RI and RII holoenzymes. Disease mutations in the RIa subunit, in particular, are a treasure chest of information that emphasizes the complexity of the allosteric network. Some of these mutations are activating and lead to *Carney Complex Disease* while others are inhibitory and lead to *Acrodysostosis*. In addition to these phenotypic differences is the tissue specificity where unique isoforms are expressed. To appreciate this complexity requires an interdisciplinary strategy that goes from molecules to cells to tissues to animals and includes not only structural biology but also cell biology, pharmacology and physiology.

**Acknowledgement:** Funded in part by NIH grants DK54441 and GM130389 to sst.

## S 5-02 Phosphodiesterase 2 promotes nitric oxide/guanylyl cyclase/cGMP signalling in heart failure

### Michael E. J. Preedy^1^, Reshma Baliga^1^, Matthew Dukinfield^1^, Sandy Chu^1^, Aisah Aubdool^1^, Kristen Bubb^1^, Amie Moyes^1^, Michael Tones^2^, Adrian J. Hobbs^1^

#### ^1^William Harvey Research Institute, Barts & The London School of Medicine & Dentistry, Queen Mary University of London, London, UK; ^2^Pfizer Inc, 700 Chesterfield Parkway West, St. Louis Missouri, US

##### **Correspondence:** Michael E. J. Preedy - m.e.j.preedy@qmul.ac.uk

*Journal of Translational Medicine* 2019, **17(2):**S 5-02

**Introduction:** Heart failure (HF) is a major cause of morbidity and mortality, and novel therapies are urgently needed. Cyclic guanosine 3′, 5′-monophosphate (cGMP) helps preserve cardiac structure and function, and impaired cGMP signaling contributes to HF pathophysiology. Cyclic GMP concentrations are regulated by synthetic pathways—triggered by natriuretic peptide- (NP) or nitric oxide- (NO) sensitive guanylyl cyclases (GCs)—in cooperation with degradation by phosphodiesterases (PDEs). The expression and activity of PDE2 is up-regulated in HF, but a causative role for this isoform in pathogenesis has not been established. We hypothesized that selective pharmacological inhibition of PDE2 promotes the cardioprotective effects of cGMP in HF, and sought to determine whether this is dependent on NP/GC-A or NO/GC-1α signaling.

**Methods:** Male C57BL/6 wild-type (WT), GC-A^−/−^ and GC-1α^−/−^ mice (21–23 g) were anesthetized (1.5% isoflurane in O_2_) and abdominal aortic constriction (AAC) performed by tying a 4-0 thread against a 25-gauge needle at the suprarenal level. Animals were assigned randomly to receive the selective PDE2 inhibitor (PDE2i) BAY 60-7550 (10 mg/kg/day) or vehicle (0.5% carboxymethylcellulose + 10% polyethylene glycol) by daily oral gavage, initiated 3 weeks (3 w) post-AAC. M-mode echocardiograms were obtained from anesthetized mice at baseline, 3 w and 6 w post-AAC using a VisualSonics Vevo 770 system. At 6 w, isolated left ventricles (LV) were cut transversely below the mitral valves, fixed in 10% formalin and embedded in paraffin wax. Tissue sections were dewaxed, rehydrated and stained using a picrosirius red kit or wheat germ agglutinin Alexa Fluor 647 antibody to assess fibrosis and cardiomyocyte size, respectively. Cyclic GMP concentrations in whole-heart homogenates were determined using commercially available kits. Results are expressed as mean ± SEM. P < 0.05 denotes significance. The n value denotes the number of animals used per group. Data were analysed by one-way ANOVA with Bonferroni’s post-test.

**Results:** AAC reduced the ejection fraction, and increased the LV internal dimeter (LVIDs), LV to body weight ratio (LV/BW), and fibrotic burden across all genotypes, confirming HF development (Fig. 1). PDE2i significantly reversed each of these indices in WT and GC-A^−/−^ mice, but had little or no effect in GC-1α^−/−^ animals. PDE2i reduced cardiomyocyte area and increased cGMP levels in WT and GC-A^−/−^, but not GC-1α^−/−^, mice.

**Fig. 1:**
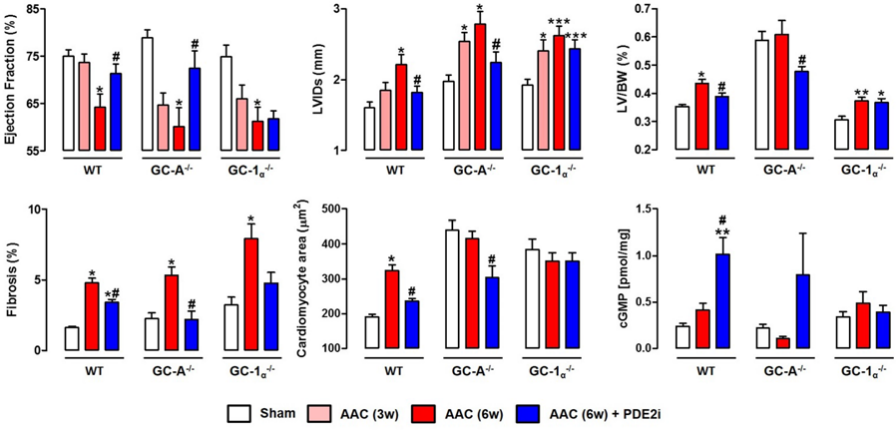
Effects of PDE2i on HF. *p < 0.05, *p < 0.01, ***p < 0.001 v respective sham; ^#^p < 0.05 v respective AAc (6 w) (n = 6–12)

**Conclusions:** These data demonstrate that PDE2i offsets the pathogenesis of experimental HF in a NO/GC-1α-dependent manner, and reveal PDE2 to be a novel, promising therapeutic target.

**Acknowledgement:** This study was supported by the British Heart Foundation.

## S 5-03 PDE3 inhibition drives a CNP/cGMP pathway to enhance cAMP signalling in HF

### Silja Meier^1^, Kjetil W. Andressen^1^, Jan Magnus Aronsen^2,3^, Ivar Sjaastad^2^, Tor Skomedal^1^, Jan-Bjørn Osnes^1^, Eirik Qvigstad^1^, Finn Olav Levy^1^, Lise R. Moltzau^1^

#### ^1^University of Oslo and Oslo University Hospital, Department of Pharmacology, Oslo, Norway; ^2^University of Oslo and Oslo University Hospital, Institute of Experimental Medical Research, Oslo, Norway; ^3^Bjørknes College, Oslo, Norway

##### **Correspondence:** Lise R. Moltzau - l.r.moltzau@medisin.uio.no

*Journal of Translational Medicine* 2019, **17(2):**S 5-03

**Introduction:** Natriuretic peptide levels are increased in heart failure (HF). Atrial (ANP) and brain (BNP) natriuretic peptide mediate their effects preferentially through the natriuretic peptide receptor (NPR)-A, and C-type natriuretic peptide (CNP) through NPR-B. NPRs are membrane bound guanylyl cyclases that increase cyclic GMP (cGMP) production when activated. We have previously shown that NPR-B stimulation by CNP enhances β_1_-adrenoceptor (β_1_-AR)- and 5-HT_4_ serotonin receptor-mediated signaling in failing hearts, probably through inhibition of phosphodiesterase (PDE) 3, a potential detrimental effect in the failing heart. In this study we examine the role of PDE2 and PDE3 in regulating the CNP-induced enhancement of β_1_-AR and 5-HT_4_ signaling in non-failing (Sham) and failing (HF) hearts.PDE2 is a dual-selective PDE and can potentially be stimulated by cGMP.

**Methods:** Chronic heart failure was induced in male Wistar rats by 6-week coronary artery ligation. Contractility studies were performed ex vivo in left ventricular muscle strips in the presence of appropriate receptor agonist and antagonists. cGMP measurements were performed on isolated left ventricular cardiomyocytes and PDE activity assays on left ventricular cardiomyocyte homogenates.

**Results:** CNP, by stimulating NPR-B, was able to enhance β_1_-AR- and 5-HT_4_-mediated inotropic responses in Sham and HF left ventricular strips. CNP elicited a similar increase of cGMP in cardiomyocytes from Sham and HF. cGMP reduced the cAMP-PDE activity of PDE3 and increased the cAMP-PDE activity of PDE2 concentration-dependently in cardiomyocyte homogenates in a similar way in Sham and HF. In Sham inhibition of PDE2 by EHNA amplified the CNP-induced enhancement of β_1_-AR- and 5-HT_4_-mediated inotropic responses. In HF PDE2 inhibition did not influence the functional effects of CNP despite increasing the cGMP response to the same marked extent as in Sham.

**Conclusions:** There is a preserved mechanism of CNP-induced enhancement of β_1_-AR and 5-HT_4_ in Sham and HF. cGMP levels and cGMP-mediated activation and inhibition of cAMP-PDE activity of PDE2 and PDE3, respectively, are similar in Sham and HF. However, PDE2 seems more involved in regulating the β_1_-AR and 5-HT_4_ enhancement in Sham compared to HF, which might reflect differences between Sham and HF in PDE2 and cyclic nucleotide compartmentation.

A version of this abstract was previously published in the proceedings from the 7th International Conference on cGMP Generators, Effectors and Therapeutic Implications [1].

**Reference**

1. BMC Pharmacol Toxicol. 2015;16(S1):A66.

## S 5-04 Rac1-sGC/cGMP crosstalk in adipocytes and vascular smooth muscle

### Alexander Pfeifer, Staffan Hildebrand

#### University of Bonn, Pharmcology and Toxikology, Bonn, Germany

##### **Correspondence:** Alexander Pfeifer - alexander.pfeifer@uni-bonn.de

*Journal of Translational Medicine* 2019, **17(2):**S 5-04

**Introduction:** Cardiovascular disease (CVD) is the leading cause of death world-wide. CVD is closely linked to dysregulation of vascular smooth muscle cell (VSMC) proliferation and migration, which has been strongly implicated in both the progression of plaque formation, and neointimal hyperplasia following clinical intervention. The nitric oxide (NO)-cGMP pathway plays a central protective role in vascular biology [1–5]. NO signaling leads to increased VSMC differentiation and relaxation through activation of its receptor soluble guanylate cyclase (sGC), leading to the generation of the second messenger cGMP. NO-cGMP signaling also potently inhibits VSMC migration and invasion. Consequently, activating the NO-cGMP pathway has led to decreased neointimal hyperplasia in several animal models.

**Results:** Recent work in our lab has identified a novel VASP-Rac1 pathway regulating sGC expression and cGMP signaling in brown adipocytes: mice deficient of VASP have increased Rac1 activity, leading to increased sGC expression and promoting adipogenic differentiation. We therefore investigated the role of VASP and Rac1 in cGMP signaling in vascular smooth muscle cells. Surprisingly, although Rac1 activation increased sGC expression in murine VSMCs, VASP^−/−^ mice have normal vascular cGMP signaling. Analysis of mice expressing constitutively active Rac1 under the control of the smooth muscle-specific Acta2 promoter also failed to show any differences in NO-mediated vascular relaxation. However, activation of Rac1 in human VSMCs strongly reduced both sGC protein and mRNA expression.

**Conclusions:** Our preliminary data indicate that activation of Rac1 reduces sGC expression in human VSMCs through interference with specific Notch ligand–receptor interactions.

**References**

1. Jennissen K, et al. A VASP-Rac-soluble guanylyl cyclase pathway controls cGMP production in adipocytes. Sci Signal. 2012;5:ra62
2. Massberg S, et al. Increased adhesion and aggregation of platelets lacking cyclic guanosine 3′,5′-monophosphate kinase I. J Exp Med. 1999;189:1255–64.
3. Aszodi A, et al. The vasodilator-stimulated phosphoprotein (VASP) is involved in cGMP- and cAMP-mediated inhibition of agonist-induced platelet aggregation, but is dispensable for smooth muscle function. EMBO J. 1999;18:37–48.
4. Pfeifer A, et al. Defective smooth muscle regulation in cGMP kinase I-deficient mice. Embo J. 1998;17:3045–51.
5. Pfeifer A, et al. Intestinal secretory defects and dwarfism in mice lacking cGMP- dependent protein kinase II. Science. 1996;274:2082–6.

## S 6-02 How stimulator compounds and nitric oxide bind and stimulate soluble guanylyl cyclase (GC1)

### William R. Montfort, Cheng-Yu Chen, Su Chung, Amanda Johnson, Andrzej Weichsel, Sarah Young, Jessica Kievenaar

#### University of Arizona, Department of Chemistry and Biochemistry, Tucson Arizona, US

##### **Correspondence:** William R. Montfort - montfort@email.arizona.edu

*Journal of Translational Medicine* 2019, **17(2):**S 6-02

**Introduction:** Our studies center on nitric oxide signaling and the nitric oxide receptor, soluble guanylyl cyclase (sGC or GC1). We seek structural insight into signal transduction; mechanism in drug-based stimulation; regulation through transmembrane protein CD47; and the role of sGC and NO in promoting breast cancer progression. Here, we focus on biophysical measurements of signal transduction in GC1 and provide the first model for stimulator compound binding.

**Methods:** Much of our biophysical data are with GC1 from the tobacco hornworm/hawkmoth. Hawkmoth GC1 is highly homologous to mammalian GC1 and responds to GC1 stimulatory compounds. One focus is on a family of truncated GC1 proteins that lack cyclase domains but respond to stimulator compounds, referred to as GC1-NT. GC1-NT constructs can be produced in large quantity and provide many advantages for structure/function studies. Studies with bacterial H-NOX proteins and mammalian GC1 are also included.

**Results:** NO and CO binding display linked equilibria with stimulator compound binding to GC1-NT, with binding of one enhancing binding of the other. Using photolabile stimulators, mass spectrometry and GC1-NT, we identified the binding pocket for stimulator compounds, which includes a heme-domain pocket. We also found that bacterial homologues of the GC1 heme domain (H-NOX proteins) bind to stimulator compounds. Using NMR spectroscopy, we have determined a full model for compound binding to the H-NOX from *Shewanella woodyi*. Mutations introduced into the binding pocket of GC1-NT confirm its role in compound binding and one such mutation mimics compound action, including tightened CO affinity and generation of a geminate recombination phase after CO photolysis. A crystal structure of an H-NOX protein with mimetic mutation indicate the changes due to mutation are small.

Additional studies suggest signal transduction proceeds through the GC1 coiled-coil ‘signaling helix’, the domain with the highest sequence homology among GC1 proteins. Studies of GC1-NT with modified coiled-coil domains display altered CO affinity to the GC1 heme and altered kinetics for NO-induced proximal histidine release. Stabilizing the coiled-coil through introduction of a disulfide bond leads to loss of CO affinity.

**Conclusions:** We propose a model for signal transduction between heme domain and cyclase domains that requires small but critical rearrangement of the coiled-coil domain. A conserved binding pocket for stimulator compounds has been identified and predominately resides in the heme domain but likely includes the interface with other GC1 domains.

## S 6-03 Structural aspects of sGC activation

### Michael A. Marletta^1^, Benjamin G. Horst^1^, Daniel J. Rosenberg^2^, Michal Hammel^2^

#### ^1^University of California Berkeley, Department of Chemistry, Berkeley California, US; ^2^Lawrence Berkeley National Laboratory, Molecular Biophysics and Integrated Bioimaging Division, Berkeley California, US

##### **Correspondence:** Michael A. Marletta - marletta@berkeley.edu

*Journal of Translational Medicine* 2019, **17(2):**S 6-03

**Introduction:** Mammalian sGC is a heterodimer composed of α- and β-subunits (Fig. 1). The C-terminus of each subunit contains a catalytic domain and the active site is composed of residues from both subunits. The catalytic domains also form a pseudosymmetric site that contains residues known to be involved in nucleotide binding, but lack the complete complement of amino acids required for catalysis. Sequence analysis shows that each subunit also contains well-defined PAS-like domain, and a predicted coiled-coil region. The N-termini of the α- and β-subunits are homologous to the H-NOX (Heme-Nitric oxide/OXygen) family of proteins. The N-terminus of β-subunit contains a ferrous heme cofactor that serves a receptor for NO. Additional studies point toward a more complicated role for NO in the regulation of activity. Structural and biochemical results have broadened the current molecular view of the regulation of sGC and provide a framework to understand the action of sGC modulators of activity.

**Fig. 1:**
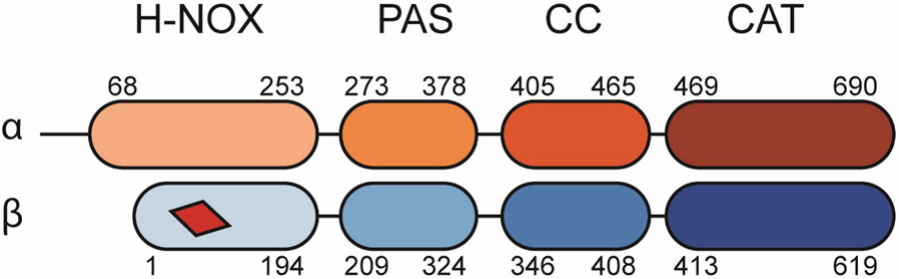
Domain organization of sGC subunits. Schematic of soluble guanylate cyclase domains. The four domains of the α (in orange) and β (in blue) subunits are listed above: *H-NOX* heme nitric oxide and oxygen binding domain, *PAS* Per-Arnt-Sim domain, *CC* coiled-coil domain, and the *CAT* catalytic domain. The approximate numbering of the Human α1 and β1 domains is shown. The heme cofactor, shown as a red diamond, binds to β1 H105

**Methods:** The quaternary conformational changes upon activation of sGC were examined using size exclusion chromatography coupled to small angle X-ray scattering (SEC-SAXS). Heterologously expressed sGC purified to homogeneity was reduced and passed over an anaerobic SEC column either with or without NO present in the running buffers. The resultant single peak was used with an in-line flow cell as the sample for SAXS at the Advanced Light Source Beamline 12.3.1. Rigid body modeling was conducted with BILBOMD to sample different conformations and select the dominant conformations.

**Results:** sGC displays clear multi-domain protein scattering curves that confirm the well-behaved state of the protein in solution. The confirmation of sGC with no NO(g) added adopts a contracted conformation with the β-subunit H-NOX bringing it in proximity to the catalytic domains. Upon addition of NO, sGC adopts two different conformations in solution: the first is the same as without NO, and the second is more extended with the H-NOX-PAS bundle moved away from the catalytic domains.

**Conclusions:** We have shown that sGC adopts distinct conformations in both the inactive and active state. The addition of NO results in a large conformational extension of sGC into the active state. Using a technique that examines sGC in solution is important to understand physiological aspects of sGC activation. The nature of how a diatomic gas causes such a large conformational change is currently under investigation.

**Acknowledgement:** We acknowledge NIH grant R01GM127854 and we thank Gregory Hura for assistance in SEC-SAXS-MALS data collection and analysis. SAXS data collection at SIBYLS (Advanced Light Source) is funded through DOE BER Integrated Diffraction Analysis Technologies (IDAT) program and NIGMS Grant P30 GM124169-01, ALS-ENABLE.

## S 6-04 News within NO/cGMP signalling

### Doris Koesling^1^, Jan Giesen^1^, Lukas Menges^1^, Ernst-Martin Füchtbauer^2^, Evanthia Mergia^1^, Michael Russwurm^1^

#### ^1^Ruhr-Universität Bochum, Pharmacology, Bochum, Germany; ^2^Aarhus University, Molecular Biology and genetics, Aarhus, Denmark

##### **Correspondence:** Michael Russwurm - michael.russwurm@rub.de

*Journal of Translational Medicine* 2019, **17(2):**S 6-04

**Introduction:** The NO/cGMP signaling cascade has an established role in physiological functions like smooth muscle relaxation, inhibition of platelet aggregation and modulation of synaptic transmission.

**Methods:** In order to be able to monitor cGMP increases in living cells, we generated a knock-in mouse that stably and ubiquitously expresses a FRET-based cGMP sensor (cGi500). With an EC_50_ of 500 nM for cGMP, the cGMP sensor allows detection of cGMP concentrations between 100 and 3000 nM.

**Results:** Here, we report on cGMP signals induced by agonists at glutamatergic NMDA- and AMPA-receptors in cultured cortical and hippocampal neurons. Although NMDA-receptor represents the prototype of the activator of the Ca^++^-dependent neuronal NO-synthase, we also observed cGMP responses induced by AMPA and studied the underlying signalling events. With neurons isolated from NO-GC1 or NO-GC2 deficient mice, we addressed the contribution of either NO-GC isoform to the NMDA- or AMPA-induced cGMP responses.

As numerous reports about NO-induced cGMP effects in the heart exist, we also aimed to measure NO-induced cGMP in cardiac cells. Whereas we failed to increase cGMP with any of the known NO-GC stimulators (NO, activators, stimulators) in cardiomyocytes even under phosphodiesterase-inhibiting conditions, NO induced tremendous cGMP increases with high potency in cardiac fibroblasts. The cGMP content in cardiac fibroblasts turned out to be as high as the one in smooth muscle cells as determined in radioimmunoassays.

**Conclusions:** Further studies are required to examine the functional role of cGMP in cardiac fibroblasts; conceivably, the NO-induced cGMP response is involved in fibrosis.

## S 6-05 Dissection of particulate and soluble guanylyl cyclase pathways based on human genetics

### Florian Sohler, Daniel Freitag

#### Bayer AG, Cardiovascular Data Science, Wuppertal North Rhine-Westphalia, Germany

##### **Correspondence:** Florian Sohler - florian.sohler@bayer.com

*Journal of Translational Medicine* 2019, **17(2):**S 6-05

**Introduction:** The particulate and soluble guanylyl cyclase (pGC and sGC) pathways regulate cGMP via the natriuretic peptide cell surface receptors and the sGC, respectively. We use functional genetic variants to describe consequences of modulating the GC pathways.

**Methods:** We queried the GWAS catalog to identify genetic variants in the cGMP pathway with known associations with blood pressure or platelet traits. For genes with more than two SNPs we identified groups of SNPs in linkage disequilibrium and selected the SNP with the strongest blood pressure association for each group.

For each selected SNP we ran linear and logistic regressions to identify associations with one of 39 quantitative and 121 disease traits within the UK biobank resource. Two-dimensional clustering on the t-statistic of the regression models was performed to identify groups of variants and traits.

We computed genetic scores for the sGC and pGC separately and investigated differences between these pathways by performing statistical heterogeneity tests.

**Results:** Genetic associations within the UK biobank show robust blood pressure effects of almost all GC pathway-modulating variants but also differences in associations with pulse pressure, platelet traits, anthropometric traits, immune cell counts and cardiovascular diseases.

Using two-dimensional clustering, we found that variants cluster according to gene and pathway (sGC vs pGC) supporting our approach of creating a genetic score for each of the pathways (see Fig. 1). Variants in the sGC pathway are strongly associated with coronary artery disease and myocardial infarction, as was previously shown [1]. pGC variants are associated with greater changes in pulse pressure than sGC variants. Furthermore, bp-lowering pGC alleles are associated with lower arterial stiffness and increased mean platelet volume (MPV) while sGC variants are associated with decreased MPV.

**Fig. 1:**
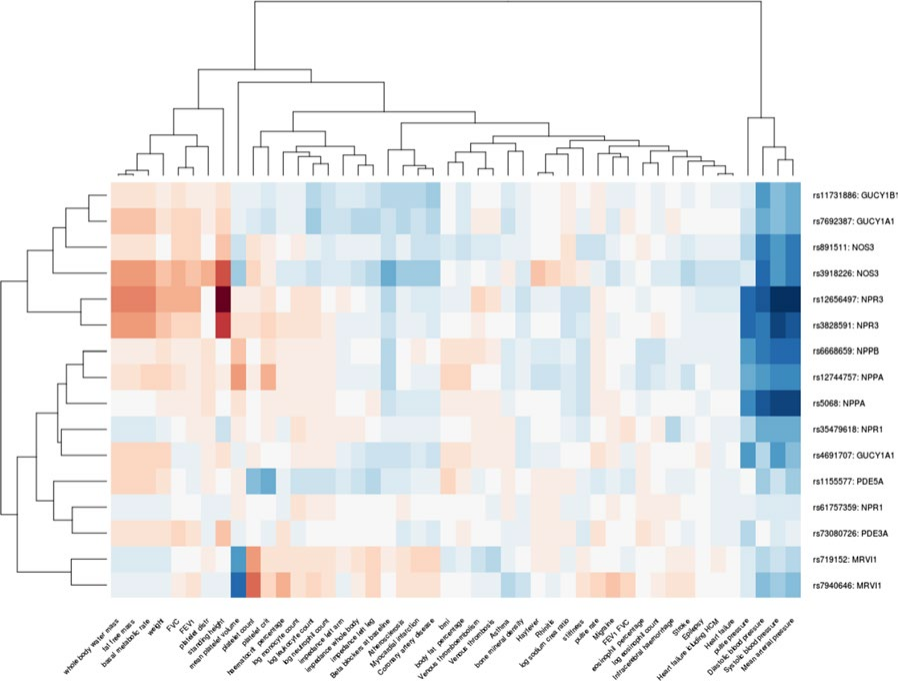
Heatmap showing clustering of genetic variants and associated traits. Each cell corresponds to the result of a linear or logistic regression. A blue cell denotes a reduction of the quantitative trait or disease risk, red denotes increase. The color intensity shows the association strength (absolute value of the t statistic). With few exceptions, the SNPs cluster according to the pGC vs sGC pathways. For the traits there is a cluster with blood pressure traits and one with anthropometric traits. There are also some interesting sub-clusters, including one containing myocardial infarction and atherosclerosis

**Conclusions:** These results highlight several differences in the genetic associations of sGC and pGC pathways and could help to identify the best therapeutic targets among the cGMP-modulating mechanisms. In particular, the protective effects for myocardial infarction seem to be specific to the sGC pathway, and variants with associations with lower MI risk are also associated with lower leukocyte counts.

The described genetic approach should be considered complementary to pharmacological investigations in animal models. While it cannot fully reflect a pharmacological intervention, it can provide first human data about effects of modulation of the cGMP pathway.

**Reference**

1. Emdin CA, et al. Phenotypic consequences of a genetic predisposition to enhanced nitric oxide signaling. Circulation. 2018;137(3):222–32.

## S 7-01 Praliciguat, a clinical-stage sGC stimulator, improved insulin sensitivity, lipid tolerance and energy utilization in a diet-induced obesity mouse model

### Chad Schwartzkopf^1^, John Hadcock^1^, Julien Roux^2^, Guang Liu^1^, Courtney Shea^1^, Mark Currie^1^, Todd Milne^1^, Jaime Masferrer^1^, Juli Jones^1^

#### ^1^Cyclerion Therapeutics, Cambridge Massachusetts, US; ^2^Biomeostasis, La Penne-sur-Huveaune, France

##### **Correspondence:** Chad Schwartzkopf - cschwartzkopf@cyclerion.com

*Journal of Translational Medicine* 2019, **17(2):**S 7-01

**Introduction:** Praliciguat (IW-1973) is a soluble guanylate cyclase (sGC) stimulator currently in clinical trials as a potential treatment for diabetic nephropathy and heart failure with preserved ejection fraction. In an exploratory, Phase 2 study in 26 patients with type 2 diabetes and hypertension on standard of care therapy, 14 days of praliciguat treatment showed positive trends in reducing fasting plasma glucose, HOMA-IR, LDL cholesterol, and triglycerides. In animal models, praliciguat distributes broadly to tissues and elicits hemodynamic, anti-inflammatory, anti-fibrotic, and metabolic effects.

**Methods:** We assessed the metabolic effects of praliciguat in the diet-induced obesity (DIO) mouse model. DIO mice were fed high-fat diet (HFD) formulated with praliciguat to achieve a C_max_ similar to a daily oral administration of 6 mg/kg for 6 weeks. Metabolic assessments began following 1 week of dosing and occurred over the subsequent 5 weeks. Obese mice maintained on control HFD and lean mice maintained on low-fat diet served as controls. In order to minimize any beneficial contribution of brown fat thermogenesis on metabolism, which is rare in humans but common in mice, mice were housed at thermoneutrality (30 °C).

**Results:** Mice treated with praliciguat had lower fasting plasma insulin, c-peptide, triglycerides and HOMA-IR than obese control mice. In addition, energy expenditure was higher in praliciguat-treated than control mice throughout a 24 h recording period, and triglycerides were lower following a lipid tolerance test. Caloric intake, body weight, and body composition were unaffected by praliciguat treatment. The HFD-induced increase in gene expression of liver tumor necrosis factor ɑ (Tnf), lipoprotein lipase (Lpl) and patatin-like phospholipase domain-containing protein 3 (Pnpla3) in control DIO mice was attenuated in praliciguat-treated DIO mice. The improvement in insulin sensitivity was associated with the restoration of liver PI3 K (pAKT-Thr308) signaling, but not MAPK (pERK) signaling.

**Conclusions:** In conclusion, in DIO mice housed at thermoneutrality, mice treated with praliciguat had an increased energy utilization, improved insulin sensitivity, lower plasma triglycerides, and improved triglyceride handling following an oral lipid challenge compared to DIO control mice. Together, these results illustrate the metabolic effects associated with praliciguat treatment in obese mice.

## S 7-02 The NO-cGMP pathway in red blood cells

### Miriam Cortese-Krott

#### Cardiovascular Research Laboratory, Division of Cardiology, Pneumology and Angiology, Medical Faculty, Heinrich Heine University, Dusseldorf, Germany

##### **Correspondence:** Miriam Cortese-Krott - Miriam.Cortese-Krott@med.uni-duesseldorf.de

*Journal of Translational Medicine* 2019, **17(2):**S 7-02

**Introduction:** Endothelial dysfunction is characterized by decreased nitric oxide (NO) bioavailability and impaired cGMP production by the NO receptor soluble guanylate cyclase(sGC) in the vasculature and in platelets. Work from our and other laboratories found that red blood cells (RBCs) play a fundamental role in systemic NO metabolism, produce NO under hypoxic conditions and carry an endothelial nitric oxide synthase (eNOS). It is unknown whether eNOS-dependent signaling occurs in RBCs and its functional significance in physiology and pathophysiology of RBCs. The talk will present the results of our current investigations aimed to demonstrate that RBCs carry a functional sGC-signaling pathway and to analyze its functional significance in vivo in mice and in clinical cohorts with coronary artery disease (CAD).

**Methods:** We isolated and determined the presence, isoform identity and activity of sGC and of proteins belonging to its downstream signaling pathway, including phosphodiesterase (PDE)-5 and protein kinase G (PKG) in RBC lysates by chromatography, western blot, mass spectrometry, and enzymatic assays using 32P-GTP or coupled to radioimmunoassay (RIA). The NO-dependent stimulation of cellular cGMP levels and VASP phosphorylation was assessed by RIA and western blot. RBC-specific sGC knock out mice were obtained by applying the Cre/LoxP approach.

**Results:** We demonstrated that human and murine RBCs carry a catalytically active α1β1-sGC (isoform 1), which converts 32P-GTP into 32P-cGMP, as well as PDE5 and PKG. Specific sGC stimulation by NO + BAY 41-2272 increases intracellular cGMP-levels up to 1000-fold with concomitant activation of the canonical PKG/VASP-signaling pathway. This response to NO is blunted in α1-sGC knockout (KO) RBCs, but fully preserved in α2-sGC KO. In patients with stable CAD and endothelial dysfunction, red cell sGC expression/activity and responsiveness to NO is fully preserved, although sGC oxidation is increased in both groups. The preliminary characterization of RBC sGC KO show specificity of the gene targeting procedure, decrease in cGMP levels in RBCs from RBC sGC KO mice, and preliminary data show changes in their hematological prophyle.

**Conclusions:** These data demonstrate that an intact sGC/PDE5/PKG-dependent signaling pathway exists in RBCs, which remains fully responsive to NO and sGC stimulators/activators in patients with endothelial dysfunction. Characterization of RBC sGC KO mice will reveal the significance of this pathway in RBC physiology and pathophysiogy.

## S 7-03 Inhaled nitric oxide as a therapeutic strategy for neurological disorders

### Nikolaus Plesnila

#### Institute for Stroke and Dementia Research (ISD), University of Munich Medical Center, Experimental Stroke Research, Munich, Germany

##### **Correspondence:** Nikolaus Plesnila - Nikolaus.Plesnila@med.uni-muenchen.de

*Journal of Translational Medicine* 2019, **17(2):**S 7-03

**Introduction:** Neurological disorders, such as ischemic and hemorrhagic stroke and traumatic brain injury, often involve the cerebral vasculature. Production of nitric oxide (NO) by endothelial cells is essential for proper function of cerebral vessels and adequate perfusion of the brain parenchyma. Consequently, reduced endothelial NO production, as often observed under pathological conditions, further damages the brain and results in additional loss of neuronal function.

Various strategies were developed to restore endothelial NO signaling, however, many approaches suffer from systemic side effects, e.g. hypotension, due to lack of spatial specificity, i.e. delivery of NO to affected but also unaffected vascular beds.

**Methods:** Data currently presented was mainly acquired using one and two-photon fluorescence microscopy of mouse cerebral microcirculation in vivo treated with inhaled NO (iNO, 50 ppm) following acute brain injury by ischemic stroke, subarachnoid hemorrhage, and traumatic injury. Brain lesions were quantified by histopathology and functional outcome by behavioral analysis.

**Results:** The current presentation will focus on our observation that inhaled NO is transported from the lung to the brain where it is released only in vascular territories with reduced partial pressure of oxygen. This property of inhaled NO can be exploited to direct NO towards vascular beds suffering from low blood flow thus improving cerebral perfusion, reducing vascular inflammation, and protecting the brain from further damage.

**Conclusions:** Experimental data in models of ischemic and hemorrhagic stroke and traumatic brain injury suggest that inhaled NO may be a valid therapeutic tool to reduce brain damage after acute injury.

## S 7-04 Essential role of guanylate cyclase-1α in platelets in the protective effects of NO inhalation after cardiac arrest and resuscitation in mice

### Fumito Ichinose

#### Department of Anesthesia, Critical Care, and Pain Medicine, Massachusetts General Hospital, 55 Fruit street, Boston, Massachusetts

##### **Correspondence:** Fumito Ichinose - fichinose@mgh.harvard.edu

*Journal of Translational Medicine* 2019, **17(2):**S 7-04

**Introduction:** Inhaled nitric oxide (NO) improves neurological and cardiovascular outcomes and survival after experimental cardiac arrest and cardiopulmonary resuscitation (CA/CPR) in multiple species. Biological effects of NO are mediated by activation of guanylate cyclase (GC). However, mechanisms responsible for the protective effects of NO inhalation after CA/CPR are incompletely understood. The objective of this study is to elucidate the mechanisms responsible for the beneficial effects of inhaled NO after CA/CPR.

**Methods:** Effects of NO breathing on outcomes after CA/CPR were studied in adult male C57BL/6J wild-type (WT) mice and mice deficient for the 1α subunit of GC (GC-1αKO). Mice were subjected to potassium chloride-induced cardiac arrest and resuscitated by manual CPR and breathed air or NO in air thereafter. To determine the role of GC in bone marrow (BM)-derived cells in the protective effects of inhaled NO, we subjected chimeric WT mice with WT BM and WT mice with GC-1αKO BM to CA/CPR with or without NO inhalation. Role of GC-1α in platelets in the beneficial effects of inhaled NO was further examined by studying platelet-specific GC-1α conditional knockout mice (GC-1αKO^PLT^) subjected to CA/CPR.

**Results:** Cardiac arrest and CPR increased peripheral polymorphonuclear neutrophils (PMN), inflammatory cytokines and chemokines, and P-selectin positive platelets. Post-CA mice exhibited a marked entrapment of red blood cells in the cerebral microcirculation and shortened tail bleeding time. Breathing NO attenuated the increments in PMN and hypercoagulability in WT, but not in GC-1αKO mice. Similarly, NO breathing attenuated the CA/CPR-induced pulmonary artery hypertension and biventricular dysfunction in WT, but not in GC-1αKO mice. Inhaled NO markedly improved 10-day survival rate and neurological function scores in WT mice with WT BM, but not in WT mice with KO BM after CA/CPR. Beneficial effects of inhaled NO on survival and neurological outcomes were abolished by platelet-specific deletion of GC-1α in mice. These observations suggest critical role of GC in platelets and other BM-derived cells in the beneficial effects of inhaled NO after CA/CPR.

**Conclusions:** These observations revealed an essential role of NO/GC-dependent signaling in BM-derived-cells, in particular platelets, in the protective effects of inhaled NO against neurological and cardiovascular injury after CA/CPR.

## S 7-05 Function of pericytes during organ fibrosis

### Dieter Groneberg, Annemarie Aue, Fabian Schwiering, Nils Englert, Amelie Reigl, Andreas Friebe

#### University Würzburg, Physiology, Würzburg Bavaria, Germany

##### **Correspondence:** Dieter Groneberg - dieter.groneberg@uni-wuerzburg.de

*Journal of Translational Medicine* 2019, **17(2):**S 7-05

**Introduction:** Normal wound healing occurs in several overlapping phases: inflammation, angiogenesis, matrix deposition, and cell recruitment. Fibrosis is a pathological feature of most chronic inflammatory diseases. In fibrosis, compared to wound healing, the matrix deposition is excessive resulting in destruction of normal tissue architecture which may lead to organ failure. In fibrogenesis and angiogenesis pericytes play a major role. We have previously shown that pericytes in several different tissue express high amounts of NO-GC.

**Methods:** To characterize the fibrotic response, we performed lineage tracing using cell/promoter-specific expression of the red fluorescent dye tdTomato. We identified several suitable Cre lines to linage trace NO-GC expressing pericytes in several organs (lung, liver, skin and heart). Using confocal microscopy, we traced NO-GC^+^-pericytes during fibrosis in these organs.

**Results:** NO-GC is highly expressed in pericytes, as judged by colocalization with different pericyte markers (desmin, NG2 and PDGFRß) and by its perivascular localization. During the fibrotic response, pericytes proliferate in lung, liver and skin. Following CCl_4_-induced liver injury NO-GC^+^ pericytes respond by migrating into the wounded area and by increasing NO-GC expression. In the lung, bleomycin induced the formation of two types of PDGFRß-positive myofibroblasts which differ in NO-GC expression and tissue localization. NO-GC^+^ pericytes differentiate into αSMA-expressing myofibroblast which constantly expresses PDGFRβ and proliferate within the alveolar wall. We also identified NO-GC^−^ myofibroblasts which show de novo PDGFRβ synthesis and occupy the alveolar space. After repetitive damage, intra-alveolar myofibroblasts disappear whereas NO-GC^+^ pericytes remain in the alveolar wall. In both liver and lung, pericyte-specific deletion of NO-GC augmented the severity of CCl_4_/bleomycin-induced fibrosis.

**Conclusions:** Clearly, NO-GC-expressing pericytes are of paramount importance for wound healing and fibrosis in several different organs. Therefore, the NO/cGMP cascade may be an attractive target for pharmacological modulation in a variety of fibrotic human diseases.

## S 7-06 New FRET-based cyclic GMP biosensors measure low cGMP concentrations in cardiomyocytes and neurons

### Gaia Calamera^1,2^, Dan Li^3^, Andrea H. Ulsund^1,2^, Jeong Joo Kim^4^, Oliver C. Neely^3^, Lise R. Moltzau^1,2^, Marianne Bjørnerem^1,2^, David Paterson^3^, Choel Kim^4,5^, Finn Olav Levy^1,2^, Kjetil W. Andressen^1,2^

#### ^1^University of Oslo and Oslo University Hospital, Department of Pharmacology, Oslo, Norway; ^2^University of Oslo and Oslo University Hospital, Center for Heart Failure Research, Oslo, Norway; ^3^Oxford University, Department of Physiology, Anatomy and Genetics, Oxford, UK; ^4^Baylor College of Medicine, Department of Pharmacology and Chemical Biology, Houston Texas, US; ^5^Baylor College of Medicine, Verna and Marrs McLean Department of Biochemistry and Molecular Biology, Houston Texas, US

##### **Correspondence:** Gaia Calamera - gaia.calamera@studmed.uio.no

*Journal of Translational Medicine* 2019, **17(2):**S 7-06

**Introduction:** Several FRET (Fluorescence Resonance Energy Transfer)-based biosensors for intracellular detection of cyclic nucleotides have been designed in the past decade. However, few such sensors are available for cGMP and even fewer that detect low nanomolar cGMP concentrations. Our aim was to develop a FRET-based cGMP biosensor with high affinity for cGMP as a tool for intracellular signalling studies.

**Methods:** We used the carboxyl-terminal cyclic nucleotide binding domain of *Plasmodium falciparum* cGMP-dependent protein kinase flanked by different FRET pairs to generate two new cGMP biosensors (Yellow *Pf*PKG and Red *Pf*PKG). Biosensors were expressed in HEK293 cells, rat cardiomyocytes and stellate ganglion neurons and affinity, selectivity and responses to activating guanylyl cyclase A (GC-A), guanylyl cyclase B (GC-B) or soluble guanylyl cyclase (sGC) was measured.

**Results:** Here, we report that these cGMP biosensors display high affinity for cGMP (EC_50_ of 20 ± 6 nM) and detect cGMP produced through sGC and GC-A in stellate ganglion neurons and GC-B in cardiomyocytes. These sensors are therefore optimal tools for real-time measurements of low concentrations of cGMP in living cells.

**Conclusions:** These sensors are optimal tools for real-time measurements of low concentrations of cGMP in living cells.

## S 8-01 Novel PDE10 inhibitors with anticancer activity that suppress Wnt-induced β-catenin transcription by activating cGMP/PKG signaling

### Gary Piazza

#### University of South Alabama/ADT Pharmaceuticals LLC, Mitchell Cancer Institute, Mobile Alabama, US

##### **Correspondence:** Gary Piazza - gpiazza@health.southalabama.edu

*Journal of Translational Medicine* 2019, **17(2):**S 8-01

**Introduction:** We and others have reported that PDE10 is overexpressed in certain cancer types and associated with reduced survival among specific groups of cancer patients. Genetic silencing of PDE10 expression or inhibition of enzymatic activity by inhibitors such as Pf2545920 can suppress the growth of tumor cells expressing high PDE10 levels, while cells from normal tissues with low PDE10 expression have reduced sensitivity. The mechanism of growth inhibition appears to involve the activation of cGMP/PKG signaling to phosphorylate and induce degradation of the oncogenic pool of β-catenin to block Wnt-induced Tcf-transcription of proteins essential for tumorigenesis. Pf2545920 suppressed tumor growth in a mouse model of colon cancer, but caused sedation.

**Methods:** To develop new PDE10 inhibitors with improved anticancer activity that do not cross the blood brain barrier, a focused library of indenes was screened for PDE10 and tumor cell growth inhibitory activity.

**Results:** Two chemically distinct lead compounds were identified with different tissue distribution patterns following oral administration. One lead compound coded as MCI-030 achieved high concentrations in colonic mucosa and suppressed tumor formation in the *APC*^min^ mouse model of colon cancer. A second lead compound, MCI-048, achieved high concentrations in lungs and was effective in multiple mouse models of lung cancer. Both compounds were efficacious at dosages that were well tolerated and represent drug development candidates.

**Conclusions:** These observations provide evidence that PDE10 is overexpressed in certain cancers and essential for tumor cell growth. We have identified novel inhibitors with strong anticancer activity in vivo without discernable toxicity.

## S 8-02 Phosphodiesterase 9A regulates nitric-oxide-independent cGMPsignaling in brain

### Frank S. Menniti^1^, John F. Harms^2^, Christopher J. Schmidt^3^

#### ^1^The George & Anne Ryan Institute for Neuroscience, University of Rhode Island, Kingston Rhode Island, US; ^2^Internal Medicine Research Unit, Pfizer Global Research and Development, Cambridge Massachusetts, US; ^3^Pfizer Innovation and Research Lab Unit, Pfizer Global Research and Development, Cambridge, US

##### **Correspondence:** Frank S. Menniti - mennitifs@gmail.com

*Journal of Translational Medicine* 2019, **17(2):**S 8-02

**Introduction:** PDE9A is a cGMP-specific phosphodiesterase expressed in neurons throughout the brain that has attracted attention as a therapeutic target to treat cognitive disorders. Indeed, PDE9A inhibitors are under evaluation in clinical trials as a treatment for Alzheimer’s disease and schizophrenia. However, little is known about the cGMP signaling cascades regulated by PDE9A. Canonical cGMP signaling in brain follows the activation of neuronal nitric oxide synthase (nNOS) and the generation of nitric oxide, which activates soluble guanylyl cyclase and cGMP synthesis. We investigated whether PDE9A regulates a cGMP pool whose synthesis is driven by nNOS signaling.

**Methods:** Increases in cGMP levels were measured in mouse cortex, hippocampus, and striatum after pharmacological inhibition of PDE9A. We investigated the effects on the PDE9A inhibitor-induced cGMP increases of pharmacological manipulation of glutamate and dopamine signaling, two neurotransmitter systems that signal via nNOS. We also examined the effects of genetic deletion of nNOS, iNOS, eNOS, PDE10A, and PDE1B on the PDE9A inhibitor-induced cGMP increases.

**Results:** Pharmacological inhibition of PDE9A produced robust increases in cGMP levels in cortex, hippocampus, and striatum. Pharmacological manipulation of glutamate and dopamine signaling or genetic deletions of nNOS, iNOS, eNOS, PDE10A, and PDE1B had no significant effects on the PDE9A inhibitor-induced cGMP increases.

**Conclusions:** Our analysis indicates that in mice, PDE9A regulates a pool of cGMP that is independent of nNOS, specifically, and nitric oxide signaling in general. This PDE9A-regulated cGMP pool appears to be highly compartmentalized and independent of cGMP pools regulated byPDE1B and PDE10A. These findings provide a new foundation for study of the upstream and downstream signaling elements regulated by PDE9A and its potential as a therapeutic target for brain disease.

**Acknowledgement:** This work was funded by Pfizer Inc and the authors were employed by Pfizer when these studies were conducted.

## S 8-03 PDE10A-cGMP signaling and pathological vascular remodeling

### Lingfeng Luo, Bradford Berk, Chen Yan

#### University of Rochester School of Medicine and Dentistry Rochester NY, Aab Cardiovascular Research Institute, Rochester New York, US

##### **Correspondence:** Chen Yan - chen_yan@urmc.rochester.edu

*Journal of Translational Medicine* 2019, **17(2):**S 8-03

**Introduction:** Intimal hyperplasia is a hallmark of vascular occlusive disorders, including post-angioplasty restenosis, vein bypass graft stenosis and accelerated atherosclerosis. Abnormal growth and accumulation of synthetic smooth muscle cells (SMCs) in the intimal lesion are a major underlying cause. Our genome-wide association studies among 17 inbred mouse strains have linked cyclic nucleotide phosphodiesterase 10A (PDE10A) to carotid artery intimal hyperplasia. However, PDE10A function in cardiovascular system remains unknown.

**Methods:** To study the role and underlying mechanism of PDE10A in the pathogenesis of vascular SMCs and structural remodeling, we used in vitro primary cultured SMCs, human saphenous vein explants, as well as in vivo mouse femoral artery injury models. PDE10A selective inhibitor TP-10 and global PDE10A knock out mice were also used.

**Results:** We observed that PDE10A expression was low in contractile SMCs but drastically elevated in synthetic SMCs in vitro and in various mouse vascular injury models in vivo. Additionally, PDE10A was highly induced in neointimal SMCs of human atherosclerotic lesions. More importantly, injury-induced neointimal formation was significantly attenuated by PDE10A deficiency or PDE10A inhibitors in vivo. PDE10A inhibition also suppressed vascular remodeling of human saphenous vein explants ex vivo. In cultured synthetic SMCs, the expression of PDE10A was further induced by growth factors or inflammatory cytokines. PDE10A depletion by RNA interference or PDE10A inhibition attenuated SMC proliferation in vitro with a cell cycle arrest at G1 phase and down-regulation of cyclin D1. Interestingly, PDE10A inhibition did not apparently induce SMC death. Mechanistic studies revealed that PDE10A regulated SMC proliferation by modulating cyclic GMP signaling, which was seemingly associated with natriuretic peptide receptors.

**Conclusions:** These results support a critical role for PDE10A in synthetic SMC growth and vascular hyperplasia remodeling, and suggest that PDE10A may represent a novel molecular target for proliferative vascular disorders.

## P1 A search for a new allosteric binding site on the natriuretic peptide receptor A

### Henriette Andresen^1^, Jerid Robinson^2^, Deborah M. Dickey^2^, Finn Olav Levy^1^, Lincoln R. Potter^2^, Lise R. Moltzau^1^

#### ^1^University of Oslo and Oslo University Hospital, Department of Pharmacology, Oslo, Norway; ^2^University of Minnesota Medical School, Department of Biochemistry, Molecular Biology, and Biophysics, Minneapolis Minnesota, US

##### **Correspondence:** Henriette Andresen - henriette.andresen@medisin.uio.no

*Journal of Translational Medicine* 2019, **17(2):**P1

**Introduction:** Enhancement of the natriuretic peptide (NP) system has become an attractive therapeutic target since NPs and their receptors are central regulators of cardiovascular and renal homeostasis. Atrial (ANP) and brain NP (BNP) activate NP receptor (NPR)-A and C-type NP (CNP) activates NPR-B. NPRs are membrane bound guanylyl cyclases (GCs) that catalyse the production of cGMP upon activation. These receptors consist of an extracellular ligand-binding domain, a transmembrane domain and intracellular kinase homology domain (KHD), coiled-coil domain (CCD) and GC domain (GCD). We have previously identified small molecular allosteric enhancers that increased the activity of NPR-A, but not NPR-B. In order to optimize our compounds further, we want to elucidate the localisation of the allosteric binding site and understand their mechanism of action.

**Methods:** Chimeric NPR-A/NPR-B receptors were constructed using In-Fusion HD plus cloning kit. Compounds ability to modulate the efficacy of BNP and CNP in chimeric NPR-A/NPR-B receptors were investigated in transiently transfected cells and the cGMP production was measured using AlphaScreen assay for cGMP. Modulation of the enzyme activity of the GCD was investigated in substrate-velocity assays in membranes from NPR-A expressing cells and cGMP production was measured by ELISA. Radioligand binding assays were performed in membrane preparations and whole cells using ^125^I-ANP as radioligand.

**Results:** We engineered chimeric NPR-A/NPR-B receptors where the whole extracellular or intracellular domains were swapped between the receptors, in order to determine which domain or domains convey selectivity to the allosteric enhancers. Our studies showed that the compounds most likely bind to the KHD in NPR-A, but the CCD also seems to be involved. In contrast to effects observed using whole cells, none of the compounds were able to increase the guanylyl cyclase activity in substrate-velocity assays or in cGMP assays using broken cells. Similarly, none of the compounds modulated the affinity of natriuretic peptide binding in membrane preparations but increased the overall affinity of NPR-A to ANP in whole cell binding assays.

**Conclusions:** Our findings suggest that our allosteric enhancers bind the KHD and a mechanism of action that requires cell integrity.

## P2 Safety, pharmacodynamic and pharmacokinetic characterisation of vericiguat: key results from six phase I studies in healthy subjects

### Michael-Friedrich Boettcher^1^, Dirk Thomas^2^, Wolfgang Mueck^1^, Stephanie Loewen^3^, Erich Arens^6^, Kenichi Yoshikawa^5^, Jane Liu^4^, Corina Becker^1^

#### ^1^Bayer AG, Clinical Pharmacology, Wuppertal, Germany; ^2^Bayer AG, Experimental Medicine, Wuppertal, Germany; ^3^Chrestos Concept GmbH & Co. KG, Essen, Germany; ^4^Bayer Healthcare Co. Ltd., Pharmaceuticals, Development Beijing, PK Asia/China, Beijing, China; ^5^Bayer Yakuhin, Ltd, Research & Development Japan, Pharmaceuticals, Clinical Sciences, Osaka, Japan; ^6^Bayer AG (employee at time of conduct and evaluation of the studies), Clinical Pharmacology, Wuppertal, Germany

##### **Correspondence:** Corina Becker - corina.becker@bayer.com

*Journal of Translational Medicine* 2019, **17(2):**P2

**Introduction**

**Background:** Vericiguat is a once-daily oral soluble guanylyl cyclase (sGC) stimulator in clinical development for treatment of chronic heart failure (HF).

**Purpose:** To characterise safety, pharmacodynamic (PD) and pharmacokinetic (PK) properties of vericiguat in healthy males. Bioavailability (BA) and effect of food on vericiguat PK were also studied.

**Methods:** Vericiguat (oral solution/immediate-release [IR] tablet) was administered to healthy White, Chinese and Japanese men as single doses (SDs; 0.5–15 mg) or multiple doses (MDs; 1.25–10 mg; once-daily for 7 days) in six phase I studies. Safety, PD and PK profiles of vericiguat were assessed.

**Results:** Overall, 265 subjects were randomised and 255 completed their respective studies. There were no deaths or serious adverse events (AEs). In the first in human, single-ascending dose study (oral solution, fasted state in White subjects), the most common drug-related AEs in vericiguat-treated subjects were headache (5 [8.9%] vs 0 in placebo) and postural dizziness (5 [8.9%] vs 0 in placebo), consistent with the vasodilatory effect of vericiguat. Increases in heart rate (HR; < 10 bpm), vasoactive hormones and changes in cardiac impedance were observed at ≥ 5 mg vericiguat relative to placebo. The 15 mg dose (oral solution, fasted) was not well tolerated due to exaggerated PD effects of orthostatic reactions in 3 of 4 subjects. Mean vericiguat exposure (C_max_ and AUC) increased with dose (0.5–15 mg: 17.2–430 µg L^−1^ and 273–7900 µg h L^−1^, respectively).

In the MD study in Japanese subjects, SDs and MDs of 1.25–10 mg vericiguat for 7 days were well tolerated. Effects of vericiguat on HR corresponded to compensatory increases in HR following expected vasodilation. Vericiguat (IR tablet, fasted state) was rapidly absorbed (median time to reach C_max_ [t_max_]: 1.0–2.5 h) with low inter-individual variability in exposure. Observed half-life was 18–27 h. No evidence for deviation from dose-proportionality was observed. Slight accumulation in AUC was observed with MDs (accumulation indices for AUC and C_max_ were 1.40–1.66 and 1.16–1.44, respectively). In White subjects, administration of vericiguat IR tablet with food increased BA by 19%, reduced PK variability and prolonged vericiguat absorption (median t_max_: 1–1.5 h [fasted]; 4 h [fed]).

**Conclusions:** SDs and MDs of vericiguat up to 10 mg were generally well tolerated. AEs and PD changes were consistent with the mechanism of action. Vericiguat PK was linear with low inter-individual variability; the half-life indicated suitability for once-daily dosing. The PK, PD and safety profile of vericiguat was in line with the expected effects of an sGC stimulator and consistent across populations studied (Fig. 1).

**Fig. 1:**
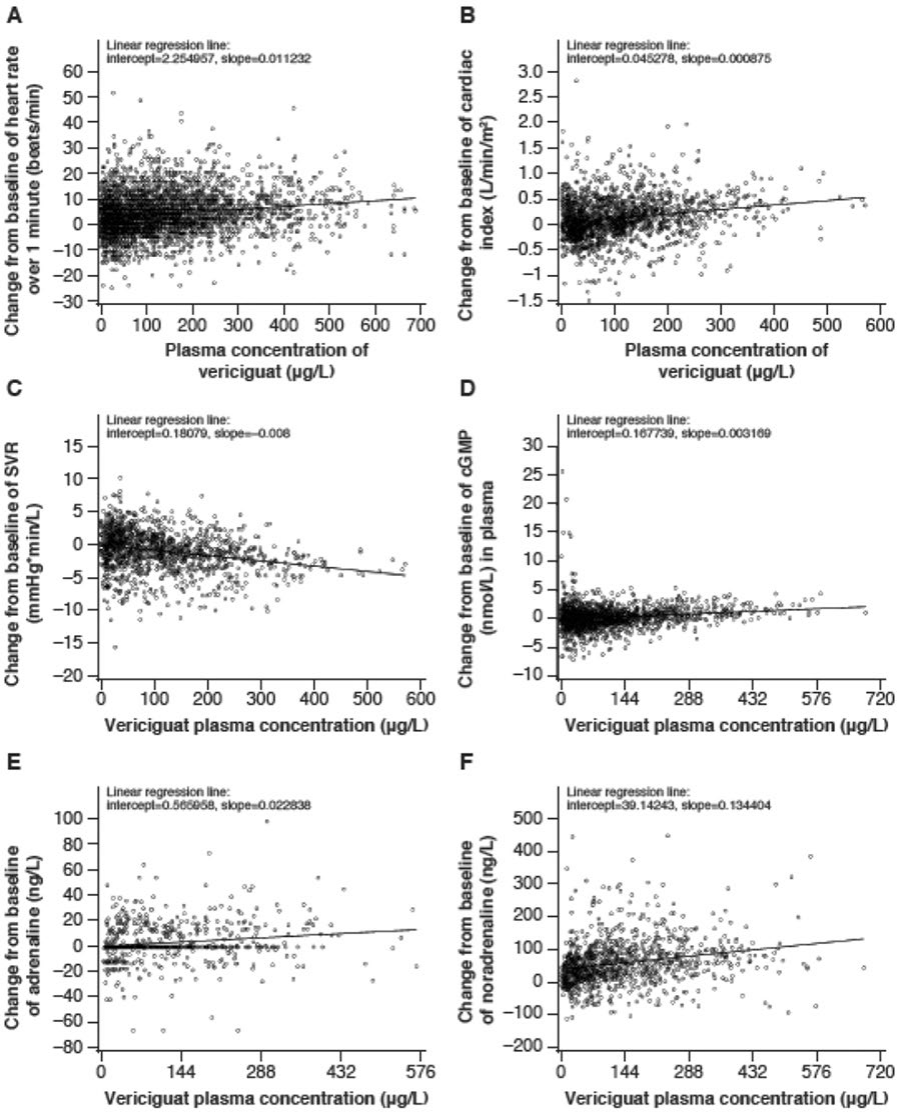
Relationship between vericiguat concentrations and hemodynamics/biomarkers. Relationship between vericiguat PK and **a** HR over 1 min, **b** cardiac index, **c** SVR, **d** cGMP, **e** adrenaline and **f** noradrenaline

**Funding:** Funding for this research was provided by Bayer and Merck Sharp & Dohme Corp., a subsidiary of Merck & Co., Inc., Kenilworth, NJ, USA.

A version of this abstract was previously published in the European Journal of Heart Failure [1].

**Reference**

1. Eur J Heart Fail. 2019;21(S1):P1182.

## P3 Soluble guanylyl cyclase (sGC) activator effects in kidney pathophysiology

### Agnes Benardeau^1^, Daniel Stehle^2^, Robert Feil^2^, Andreas Patzak^3^, Karen Griffin^4^, Anil Bidani^4^, Peter Sandner^1^, Frank Eitner^1^

#### ^1^Bayer AG, Drug Discovery, Pharmaceuticals, Wuppertal North Rhine-Westphalia, Germany; ^2^Eberhard Karls Universität Tübingen, Interfakultäres Institut für Biochemie (IFIB), Tübingen Baden-Württemberg, Germany; ^3^Charité - Universitätsmedizin Berlin, Johannes-Müller-Centrum für Physiologie, Berlin Berlin, Germany; ^4^Loyola University, Medical Center, Maywood Illinois, US

##### **Correspondence:** Agnes Benardeau - agnes.benardeau@bayer.com

*Journal of Translational Medicine* 2019, **17(2):**P3

**Introduction:** Chronic kidney diseases (CKD) are associated with increased oxidative stress that changes native, wildtype sGC to oxidized, heme-free Apo-sGC which cannot be activated by NO anymore. Soluble GC activators (sGCact) bind and activate apo-sGC independently of NO, resulting in an increase in tissue cGMP levels as well as anti-proliferative and anti-platelet effects. We hypothesize that sGCact might be capable of maintaining beneficial cGMP generation even under CKD-associated oxidative stress conditions.

**Methods:** We investigated the effects of sGCact on cellular and kidney cGMP production and vasorelaxation in ex vivo and in vivo models. We measured 1/cGMP production of glomeruli from of FRET-sGC mouse kidney slices, 2/quantified isolated renal arteriole relaxation, and 3/renal blood flow and cGMP production from isolated perfused mouse kidney. Finally, 4/we measured in vivo renal blood flow and renal vascular resistance in a rat model of CKD (diabetic and hypertensive ZSF1 rats). In vitro and ex vivo investigations were performed on native sGC and on Apo-sGC.

**Results:** Under basal conditions (in the presence of NO), sGCact did neither increase cGMP production in glomeruli of FRET mice nor potentiate NO-induced cGMP signal. ODQ reduced basal and DEA/NO-induced glomerular cGMP production (from 1.1 ± 0.1 norm AUC to − 0.4 ± 0.3 norm AUC) which was completely recovered by sGCact (2.1 ± 0.5 norm AUC). Pre-contracted and NO depleted mouse renal arterioles were dilated by sGCact with a greater and faster effect on efferent (100-fold) than on afferent arterioles (70-fold). In pre-contracted and NO depleted isolated perfused mouse kidneys, sGCact dose-dependently improved renal blood flow (RBF) (relative RBF from baseline from 0.99 ± 0.01 AU to 2.80 ± 0.10 AU) and increased kidney cGMP production (from 1.2 ± 1.8 to 13.1 ± 2.9 pmol/min/g kidney). Finally, in vivo, sGCact dose-dependently increased RBF (9.5 ± 0.5 ml/min to 13.7 ± 0.9 ml/min, p < 0.01) and reduced renal vascular resistance of CKD rats (15.7 ± 1.9 mmHg/ml min to 9.5 ± 1.8 mmHg/ml min, p < 0.01).

**Conclusions:** sGCact increased renal cGMP production, vasodilated renal arterioles and increased RBF in ex vivo models under NO depletion. Similar improvements of RBF were measured in hypertensive and diabetic rats. sGCact could represent a promising new class of drugs for the treatment of patients suffering from CKD.

## P4 Soluble guanylate cyclase modulators relaxed isolated corpus cavernosum from spontaneously hypertensive rats

### Gabriela M. Bertollotto, Camila S. Estancial, Mariana G. de Oliveira, Aline S. Nicoletti, Gabriela R. Passos, Gilberto De Nucci, Edson Antunes, Fabíola Z. Mónica

#### UNICAMP, Department of Pharmacology, Campinas, Brazil

##### **Correspondence:** Gabriela M. Bertollotto - gabriela.bertollotto@gmail.com

*Journal of Translational Medicine* 2019, **17(2):**P4

**Introduction**

**Objective:** Hypertension is a risk factor for the development of erectile dysfunction (ED) and is associated with impairment of NO-sGC-cGMP pathway. This study is aimed to characterize the relaxing response induced by sGC modulators in isolated corpus cavernosum (CC) from spontaneous hypertensive rats (SHR) and Wistar Kyoto (WKY).

**Methods:** SHR and WKY 16 weeks were used. Concentration–response curves (CRCs) to BAY 41-2272, BAY 60-2770, tadalafil and acetylcholine (ACh) were carried out. In some experiments, CRCs to BAYs and ACh were also done in the presence of sGC inhibitor, ODQ or BAY 41-2272/BAY 60-2770 (100 nM), respectively. Intracellular levels of cGMP and protein expression sGCalpha1, sGCbeta1 and PDE5 were also determined. Results are expressed as mean ± SEM. Student’s *t* test or one-way ANOVA were used.

**Results:** In CC from SHR the relaxation induced by BAY 41-2272 (pEC50: 6.01 ± 0.08 and Emax = 66 ± 0.4%) or BAY 60-2770 (pEC50: 6.99 ± 0.15 and Emax = 87 ± 4%) did not differ significantly in comparison to CC from WKY (BAY 41: pEC50: 6.11 ± 0.16 and Emax = 73 ± 4% and BAY60: pEC50: 7.01 ± 0.16 and Emax = 89 ± 8%). In the presence of ODQ BAY 41-2272- or BAY 60-2770-induced relaxation was reduced and potentiated, respectively to similar magnitude in CC from WKY and SHR. Although no difference in the relaxing response induced by BAY 60-2770 was observed between groups, in CC from SHR stimulated with BAY 60-2770 (1 µM) alone or in combination with ODQ (10 µM) the cGMP levels were 3- and 2-times greater in comparison to CC from WKY. In CC stimulated with sodium nitroprusside, the cGMP levels were reduced in SHR than in WKY. The relaxation induced by ACh was reduced in CC from SHR (Emax: 3.4 ± 1.4%) in comparison with WKY (Emax: 9.8 ± 1.2%). In the presence of BAY 41-2272 (WKY Emax: 15 ± 1.3% and SHR Emax: 8.7 ± 0,4%) or BAY 60-2770 (Emax WKY: 16 ± 0.7% and SHR: 9.4 ± 1.8%) a significant potentiation in ACh-induced relaxation was observed. The relaxing response induced by tadalafil was significantly lower in CC from SHR (pEC50: 6.03 ± 0.41 Emax: 25 ± 8%) than in WKY (pEC50: 5.81 ± 0.19 Emax: 59 ± 9%). Protein expression for PDE5 was greater in CC from SHR than in WKY, while no difference was observed for sGCalpha1 and sGCbeta1.

**Conclusions:** sGC stimulator and activator are highly effective at relaxing CC from SHR. Thus our study provides a rationale for the use of sGC modulators in patients with ED, especially in those unresponsive to PDE5 inhibitors, but clinical trials are required.

**Acknowledgement**

**Disclosures:** none

**Ethical approval:** All experimental protocols were approved by the Animal Ethical Committee of UNICAMP (CEUA/UNICAMP: 3516-1).

**Financial support:** Sao Paulo Research Foundation (FAPESP 2017/15175-1; 2018/07364-1).

## P5 sGC stimulation and pde5 inhibition decrease sinusoidal resistance and reduce fibrosis in rats with biliary

### Ksenia Brusilovskaya^1,4^, Philipp Königshofer^1,4^, Daniel Lampach^1,4^, Adrian Szodl^1,4^, Paul Supper^1,4^, David Bauer^1,4^, Andrea Beer^2^, Judith Stift^2^, Bruno K. Podesser^3^, Martha Seif^1,4^, Kerstin Zinober^1,4^, Nataliya Rohr-Udilova^1,4^, Michael Trauner^1,4^, Thomas Reiberger^1,4^, Philipp Schwabl^1,4^

#### ^1^Medical University of Vienna, Inner medicine III/Gastroenterology and Hepatology, Vienna Wien, Austria; ^2^Medical University of Vienna, Pathology, Vienna Wien, Austria; ^3^Medical University of Vienna, Center of Biomedical Research, Vienna Wien, Austria; ^4^Medical University of Vienna, Hepatic Hemodynamic Laboratory, Vienna Wien, Austria

##### **Correspondence:** Ksenia Brusilovskaya - ksenia.brusilovskaya@meduniwien.ac.at

*Journal of Translational Medicine* 2019, **17(2):**P5

**Introduction:** Liver cirrhosis is a leading cause of death in the Western world, while portal hypertension (PHT) is the main driver for severe complications in cirrhotic patients. In cirrhotic PHT, the altered NO/sGC/cGMP pathway contributes to fibrosis development and endothelial dysfunction. Current medical treatment of PHT is limited to non-selective beta-blockers, while there is no special treatment against liver fibrosis. Modulation of sGC activity presents a beneficial approach to promote vasodilation, in line with fibrosis prevention. In bile duct ligated rats (BDL), we investigated the sGC stimulator riociguat (RIO), sGC activator cinaciguat (CINA) and phosphodiesterase-5 inhibitor tadalafil (TADA).

**Methods:** Male Sprague–Dawley rats underwent BDL or sham-operation. Starting 1 week after surgery—RIO (0.5 mg/kg), CINA (1 mg/kg), TADA (1.5 mg/kg) or vehicle (VEH) were gavaged for 3 weeks. Portal pressure (PP), mean arterial pressure, heart rate and splanchnic/portal blood flow were measured as a primary outcome parameter. Additionally, liver fibrosis, hepatic inflammation, and hepatic cGMP levels were assessed.

**Results:** Cirrhotic BDL-VEH rats showed a significant increase in PP (13.07 ± 0.97 mmHg) compared to healthy controls. Both RIO (9.96 ± 0.7 mmHg, p = 0.021) and TADA (10.27 ± 0.86 mmHg, p = 0.050) treatment reduced PP without affecting systemic hemodynamics. Furthermore, RIO reduced intrahepatic vascular resistance (2.86 ± 0.25 vs. 4.85 ± 0.54 mmHg/min mL, p = 0.005). BDL rats developed significant fibrosis, while BDL-RIO rats presented with the strongly reduced chrome-aniline-blue stained area (2.14 ± 0.3% vs. 4.15 ± 0.53%, p = 0.011). Accordingly, hepatic hydroxyproline content was lower in BDL-RIO and BDL-TADA groups (RIO 350 ± 30 µg/g, p = 0.003; TADA 282 ± 50 µg/g, p = 0.003 vs. BDL-VEH 503 ± 20 µg/g). In line also transaminase levels were lower in the RIO and TADA groups (AST: RIO: − 36%, p < 0.001; TADA: − 24%, p = 0.006/ALT: RIO: − 32%, p = 0.035; TADA: − 27%, p = 0.053), as well as intrahepatic IL6 expression in BDL-RIO rats (− 44%, p = 0.053).Expression of hepatic cGMP was significantly enhanced by RIO, but not by TADA or CINA. In cirrhotic BDL rats, 1 mg/kg CINA caused weight loss, systemic hypotension yet no amelioration of PHT, increased lactate levels and TNF-α expression.

**Conclusions:** The sGC stimulator riociguat and the PDE5 inhibitor tadalafil showed beneficial effects in cirrhotic rats by reducing liver fibrosis and decreasing portal hypertension. High dosing of sGC activators such as 1 mg/kg cinaciguat may be associated with toxicity in cirrhosis (Fig. 1).

**Fig. 1:**
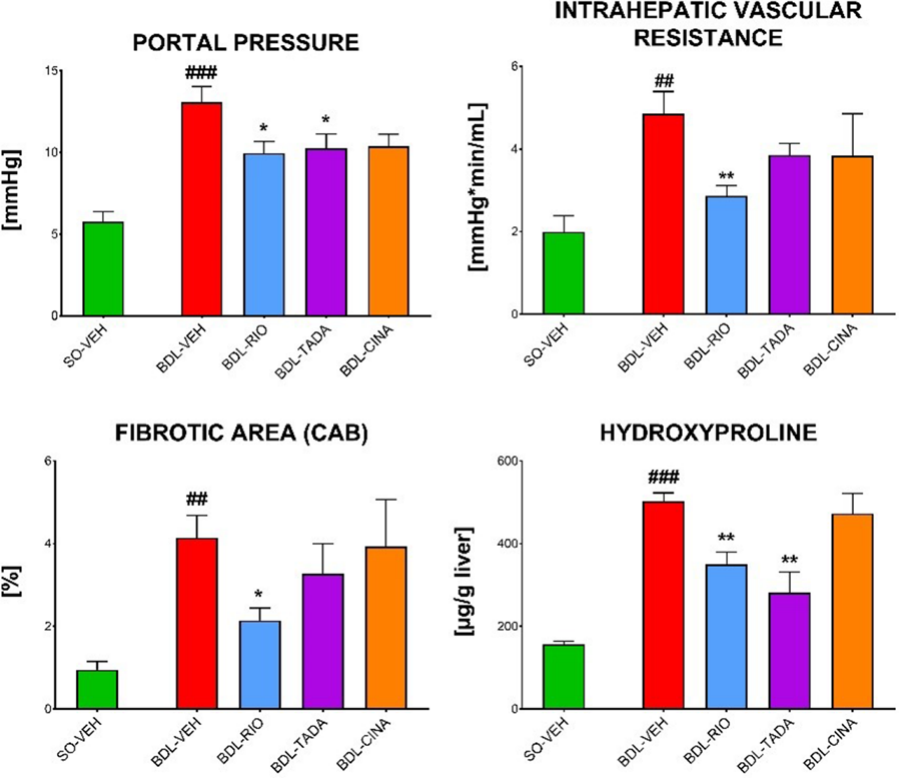
The figure present effects of different sGC modulators/PDE-5 inhibitor on portal pressure/hepatic intravascular resistance and fibrosis parameters (incl. CAB stained area and hydroxyproline content)

**Acknowledgement:** The study was supported by Boehringer-Ingelheim.

## P6 Understanding the allosteric mechanism of *Plasmodium falciparum* cGMP-dependent protein kinase by NMR

### Olivia Byun^1^, Katherine Van^1^, Jinfeng Huang^2^, Friedrich W. Herberg^3^, Choel Kim^4,5^, Giuseppe Melacini^1,2^

#### ^1^McMaster University, Department of Biochemistry and Biomedical Sciences, Hamilton Ontario, Canada; ^2^McMaster University, Department of Chemistry and Chemical Biology, Hamilton Ontario, Canada; ^3^University of Kassel, Department of Biochemistry, Kassel Hesse, Germany; ^4^Baylor College of Medicine, Department of Pharmacology, Houston Texas, US; ^5^Baylor College of Medicine, Department of Biochemistry and Molecular Biology, Houston Texas, US

##### **Correspondence:** Olivia Byun - byunja@mcmaster.ca

*Journal of Translational Medicine* 2019, **17(2):**P6

**Introduction:** Malaria is a life-threatening disease responsible for about a million fatalities per year worldwide and about half of the world population is at risk. Most of the malaria deaths are caused by *Plasmodium falciparum*. An essential regulator for the development of *P. falciparum* is the cyclic GMP (cGMP) dependent protein kinase (*Pf*PKG). *Pf*PKG is composed of a regulatory region and a catalytic domain. In the absence of cGMP, the regulatory region inhibits the kinase domain. Upon cGMP binding to the regulatory region, the inhibition is released and *Pf*PKG is activated.

**Methods:** Targeting directly the active site of *Pf*PKG poses a major selectivity challenge, since the kinase catalytic domains are highly conserved among eukaryotes. One promising approach to circumvent this problem is to selectively target less conserved regulatory regions of *Pf*PKG, such as the cGMP-binding domains (CBDs). Here, we utilize solution NMR (Nuclear Magnetic Resonance) to study the structure and dynamics of *Pf*CBD-D in its apo (unbound) and effector bound states, as well as to identify the allosteric sites, which provide an excellent opportunity for selective inhibition.

**Results:** The chemical shift covariance analysis (CHESCA) allows us to map the allosteric networks by identifying groups of residues that exhibit a concerted response to perturbations. The latter include different analogs of cGMP that induce a range of kinetic *Pf*PKG responses. Our CHESCA analysis reveals a novel allosteric network of *Pf*CBD-D. Interestingly, this identified network not only spans regions affected by cGMP-dependent structural variations, but also sites that are controlled by dynamically driven allostery.

**Conclusions:** These novel allosteric sites are expected to provide avenues for the development of potential selective inhibitors and open new therapeutic opportunities for treating malaria, decreasing recurrence rates and circumventing resistance to current malaria drugs, such as artemisinin.

## P7 Unraveling of the mechanism behind PKG dependent regulation of NOX4/5 gene expression for improved ischemic stroke therapy

### Ana I. Casas^2^, Alexander G. Grønning^1^, Friederike Langhauser^3^, Christoph Kleinschnitz^3^, Jan Baumbach^4^, Harald H. Schmidt^2^

#### ^1^University of Southern Denmark, Computational Biology, Odense, Denmark; ^2^Maastricht University, Department of Pharmacology and Personalized Medicine, Maastricht, Netherlands; ^3^University Clinics Essen, Department of Neurology, Essen, Germany; ^4^Technical University of Munich, Experimental Bioinformatics, Munich, Germany

##### **Correspondence:** Ana I. Casas - a.casasguijarro@maastrichtuniversity.nl

*Journal of Translational Medicine* 2019, **17(2):**P7

**Introduction:** Reactive oxygen species (ROS) formation is considered a key pathomechanism affecting NO-cGMP signalling by (i) uncoupling NO synthase, (ii) scavenging NO, and (iii) oxidatively damaging the NO receptor, soluble guanylate cyclase, sGC, yielding its heme-free and NO-insensitive apo-form. ROS overexpression is a key mechanism post-stroke and NADPH oxidases are considered the primary source in this pathomechanism. In fact, overexpression of NOX4 and NOX5 contribute to direct neurotoxicity and blood–brain barrier leakage.

**Methods:** To further aid the development of treatments for ischemic stroke, it is vital to elucidate the underlying mechanisms of the PKG dependent regulation of NOX4/5 gene expression. For this purpose, databases containing information about protein–protein and kinase-substrate interactions plus transcriptional regulation of genes have been used in synergy to extract pathways that can explain the phenomenon.

**Results:** Here, we identify a previously not described cross-talk mechanism where NOX4/5 ROS formation is regulated in a apo-sGCa/PKG dependent manner. Human brain microvascular endothelial cells exposed to oxygen–glucose–deprivation, apo-sGC treatment nearly completely prevented cell death while NOX4 and NOX5 are surprisingly downregulated. Similarly, in mice, which do not express the NOX5 gene, NOX4 expression upon transient middle cerebral artery occlusion was dramatically downregulated resulting in reduced oxidative and nitrative stress and reduced infarct size in a PKG dependent manner.

**Conclusions:** This approach will lead towards to a precise network pharmacology mechanistic-based stroke therapy where currently only approaches focused on single drug or symptom are available.

**Acknowledgement:** This study was supported by the ERC (Advanced Investigator Grant 294683/RadMed and Proof-of-Concept Grant 737586/SAVEBRAIN, both to H.H.H.W.S.), short-term scientific missions by the COST Actions EU-ROS and Open- MultiMed (to A.G.), and Kootstra Talented Fellowship (UM) (to A.I.C.). J.B.’s work was financially supported by VILLUM Young Investigator Grant 13154. J.B. and H.H.H.W.S. also received support from H2020 Project 777111-REPO-TRIAL.

**References**

1. Casas AI, Dao VT-V, Daiber A, Maghzal GJ, Di Lisa F, Kaludercic N, et al. Reactive oxygen-related diseases: therapeutic targets and emerging clinical indications. Antioxid Redox Signal. 2015;23(14):1171–85.
2. Casas AI, Geuss E, Kleikers PWM, Mencl S, Herrmann AM, Buendia I, Egea J, Meuth SG, Lopez MG, Kleinschnitz C, Schmidt HHHW. NOX4-dependent neuronal autotoxicity and blood–brain barrier breakdown explain the superior sensitivity of the brain to ischemic damage. Proc Natl Acad Sci USA. 2017;114(46):12315–20.
3. Langhauser F, Casas AI, Dao VTV, Guney E, Menche J, Geuss E, Kleikers PW, López MG, Barabási AL, Kleinschnitz C, Schmidt HHHW. A diseasome cluster-based drug repurposing of soluble guanylate cyclase activators from smooth muscle relaxation to direct neuroprotection. NPJ Syst Biol Appl. 2017;4(1):8.
4. Casas AI, Kleikers PWM, Geuss E, Langhauser F, Adler T, Busch DH, Gailus-Durner V, de Angelis MH, Egea J, Lopez MG, Kleinschnitz C, Schmidt HHHW. Calcium-dependent reactive oxygen formation and blood–brain barrier breakdown by NOX5 limits post-reperfusion benefit in stroke. J Clin Investig. 2019.
5. Casas AI, Hassan AA, Larsen SJ, Gomez-Rangel V, Elbatreek M, Kleikers PWM, Guney E, Egea J, Lopez MG, Baumbach J, Schmidt HHHW. From single drug targets to synergistic network pharmacology in ischemic stroke. Proc Natl Acad Sci. 2019.

## P8 PKGIα is activated by oxidation in vitro, but not in intact cells

### Sahar Aminzai, Tingfei Hu, Renate B. Pilz, Darren E. Casteel

#### University of California, San Diego, Department of Medicine, La Jolla California, US

##### **Correspondence:** Darren E. Casteel - dcasteel@ucsd.edu

*Journal of Translational Medicine* 2019, **17(2):**P8

**Introduction:** The type I cGMP-dependent protein kinases play key roles in cardiovascular physiology (1). While the enzymes are canonically downstream of nitric oxide/naturetic peptide induced cGMP generation, various groups have reported oxidation-induced direct activation of the kinase (2, 3). However, in contrast to some of these reports, we have previously shown that oxidation of PKGIα at C43 does not directly increase kinase activity (4).

**Methods:** We used in vitro and cell based assays to examine oxidation-induced PKGI activation.

**Results:** We found that PKGIα stored overnight at 4 °C became activated in an oxidation dependent manner, which was prevented in the presence of DTT. Since PKGIα C43S was activated to a similar extent as wild-type enzyme, the activation was independent of disulfide bond formation at C43. Activation was also prevented in the presence of EDTA, indicating dependence on trace metals in the elution buffer (PBS + 100 nM Flag-peptide). In addition, the higher basal activity after overnight storage in elution buffer could not be reversed by subsequent reduction with DTT, suggesting that cysteines were oxidized to sulfinic or sulfonic acid. Interestingly, under the same overnight storage conditions, PKGIβ was not activated. Similarly, Sheehe et al. recently found that short exposure of purified PKGI to H_2_O_2_ activated PKGIα to a much greater extent than PKGIβ, and proposed that once C118 was modified to an acidic form, it interacted with basic residues unique to the PKGIα autoinhibitory loop, leading to an opening of the catalytic cleft (5). We found that residues within the isoform-specific autoinhibitory loop were responsible for PKGIα activation. While this result is consistent with the activation mechanism proposed by Sheehe et al., we observed that mutation of the proposed, mechanistically-important basic residues in the PKGIα autoinhibitory loop to the corresponding non-basic residues in PKGIβ did not prevent activation. In addition, while PKGIα with a C118D mutation initially had a slightly higher basal activity than wild-type enzyme, the basal activity dramatically increased after overnight storage, indicating that the enzyme was being activated by modification at one or more additional sites. To explore whether oxidants activate PKGIα in cells, we treated rat cardiac-derived H9C2 cells with H_2_O_2_ under a variety of experimental conditions and monitored VASP phosphorylation. While we were able to observe robust PKGIα crosslinking, we were unable to detect H_2_O_2_-induced PKGIα activation in cells.

**Conclusions:** While PKGIα is activated by oxidation in vitro, whether oxidants induce PKGIα activation in cells remains unclear.

**References**

- Hofmann F, Wegener JW. cGMP-dependent protein kinases (cGK). Methods Mol Biol. 2013;1020:17–50
- Burgoyne JR, Madhani M, Cuello F, Charles RL, Brennan JP, Schroder E, Browning DD, Eaton P. Cysteine redox sensor in PKGIa enables oxidant-induced activation. Science. 2007;317:1393–7.
- Landgraf W, Regulla S, Meyer HE, Hofmann F. Oxidation of cysteines activates cGMP-dependent protein kinase. J Biol Chem. 1991;266:16305–11.
- Kalyanaraman H, Zhuang S, Pilz RB, Casteel DE. The activity of cGMP-dependent protein kinase Ialpha is not directly regulated by oxidation-induced disulfide formation at cysteine 43. J Biol Chem. 2017;292:8262–8.
- Sheehe JL, Bonev AD, Schmoker AM, Ballif BA, Nelson MT, Moon TM, Dostmann WR. Oxidation of cysteine 117 stimulates constitutive activation of the type Ialpha cGMP-dependent protein kinase. J Biol Chem. 2018;293:16791–802.

## P9 Regulation of VSMC growth and atherosclerosis by cGMP and fibronectin

### Hyazinth Dobrowinski^1^, Moritz Lehners^1^, Andreas Friebe^2^, Susanne Feil^1^, Robert Feil^1^

#### ^1^University of Tübingen, Interfakultäres Institut für Biochemie, Tübingen, Germany; ^2^University of Würzburg, Institute of Physiology, Würzburg, Germany

##### **Correspondence:** Hyazinth Dobrowinski - hyazinth.dobrowinski@uni-tuebingen.de

*Journal of Translational Medicine* 2019, **17(2):**P9

**Introduction:** It is well known that an increase of cGMP relaxes vascular smooth muscle cells (VSMCs) and, thereby, regulates vascular tone and blood flow. Upon pathophysiological changes in their environment, e.g. during development of atherosclerotic plaques, VSMCs are involved in a process named vascular remodeling. Previous studies with atherosclerotic mice have suggested that cGMP signaling affects vascular remodeling. However, it is not fully understood how cGMP derived via different cGMP generator pathways (e.g. NO-induced vs. natriuretic peptid-induced) affects VSMC growth and survival, and if cGMP signaling interacts with extracellular matrix proteins like fibronectin (Fn).

**Methods:** The impact of the cGMP signaling pathway on the growth and survival of primary VSMCs from mouse aorta was analyzed in real time using the impedance-based xCELLigence system. Control VSMCs were compared to VSMCs lacking NO-sensitive guanylyl cyclase (NO-GC) or cGMP-dependent protein kinase I (cGKI). Moreover, we investigated if Fn regulates VSMC growth behavior and phenotype, and whether this might be connected to cGMP signaling, by growth monitoring, Western blot analysis and FRET-based cGMP measurements in the presence and absence of Fn. Smooth muscle-specific Fn knockout mice (Fn-smko) were generated on an apolipoprotein E-deficient background to investigate the specific role of Fn in VSMCs for atherosclerosis.

**Results:** By comparing control VSMCs with NO-GC- and cGKI-deficient VSMCs, we found that activation of the NO-GC/cGMP/cGKI cascade promotes the growth of VSMCs without affecting their viability. In contrast, stimulation of the membrane-bound guanylyl cyclase A by atrial natriuretic peptide (ANP) had only a moderate effect on VSMC growth, but a strong pro-survival effect. Furthermore, our data showed that Fn ablation accelerates VSMC growth. Interestingly, VSMCs from Fn-smko mice had an increased activity of NO-GC. Thus, amplified NO/cGMP signaling might explain the increased growth of Fn-deficient VSMCs. Experiments with Fn-smko mice are in progress to test if Fn in VSMCs modulates the development of atherosclerotic lesions in vivo.

**Conclusions:** Our findings indicate that VSMCs have distinct cGMP pools that regulate VSMC growth and survival differently, i.e. a NO-induced pool and an ANP-stimulated pool, which promote VSMC growth and survival, respectively. These growth effects might be modulated by a novel crosstalk between Fn and cGMP signaling and affect the growth and stability of atherosclerotic plaques in vivo.

**Acknowledgement:** This work was supported by the DFG (FOR 2060 projects FE 438/5-2 and FE 438/6-2). The authors declare no competing financial interests.

## P10 The pseudokinase domains of guanylyl cyclase-A and -B allosterically increase the affinity of their catalytic domains for substrate

### Aaron Edmund^1^, Lincoln Potter^1^, Timothy Walseth^2^, Nicholas Levinson^2^

#### ^1^University of Minnesota, Biochemistry Department, Minneapolis Minnesota, US; ^2^Univeristy of Minnesota, Department of Pharmacology, Minneapolis Minnesota, US

##### **Correspondence:** Aaron Edmund - edmun043@umn.edu

*Journal of Translational Medicine* 2019, **17(2):**P10

**Introduction:** Natriuretic peptides (NPs) regulate multiple physiologic systems by activating transmembrane receptors that contain intracellular guanylyl cyclase domains, such as GC-A and GC-B, also known as Npr1/NPR-A and Npr2/NPR-B, respectively (1). Both enzymes contain an intracellular, phosphorylated, pseudokinase domain (PKD) that is critical for activation of the C-terminal cGMP-synthesizing guanylyl cyclase domain (2). Because ATP allosterically activates GC-A and GC-B (3,4), we investigated how ATP binding to the PKD influences the guanylyl cyclase activities of GC-A and GC-B.

**Methods:** The x-ray structure of the Src family kinase LCK (PDB ID: 3LCK) was used to create a homology model of the PKD, using the SWISS-MODEL server (5). Molecular modeling indicated all the residues of the ATP binding site of the prototypical kinase PKA, except the catalytic aspartate, are conserved in the PKDs of GC-A and GC-B. Kinase-inactivating alanine substitutions for the invariant lysine in subdomain II or the aspartate in the DYG-loop of GC-A and GC-Bwere created by site-directed mutagenesis. Effects of mutations on enzymatic activity were assessed by performing guanylyl cyclase assays. PyMol was used to predict how gain of function mutations affect the structure of the PKDs and guanylyl cyclase assays were used to determine if the predictions increased allosteric activity.

**Results:** Kinase-inactivating alanine substitutions for the invariant lysine in subdomain II or the aspartate in the DYG-loop of GC-A and GC-B failed to decrease enzyme phosphate content, consistent with the PKDs lacking kinase activity. In contrast, both mutations reduced enzyme activation by blocking the ability of ATP to decrease the Michaelis constant without affecting NP-dependent activation. The analogous lysine-to-alanine substitution in a glutamate-substituted phosphomimetic mutant form of GC-B also reduced enzyme activity, consistent with ATP stimulating guanylyl cyclase activity through an allosteric, phosphorylation-independent mechanism. Mutations designed to rigidify the conserved regulatory or catalytic spines within the PKDs increased guanylyl cyclase activity, increased sensitivity to NP-stimulation, or reduced the Michaelis constant in the absence of ATP, consistent with ATP binding stabilizing the PKD in a conformation analogous to that observed in catalytically active kinases.

**Conclusions:** We conclude that allosteric mechanisms evolutionarily conserved in their PKDs promote the catalytic activation of transmembrane guanylyl cyclases by increasing affinity for the substrate, GTP (Fig. 1).

**Fig. 1:**
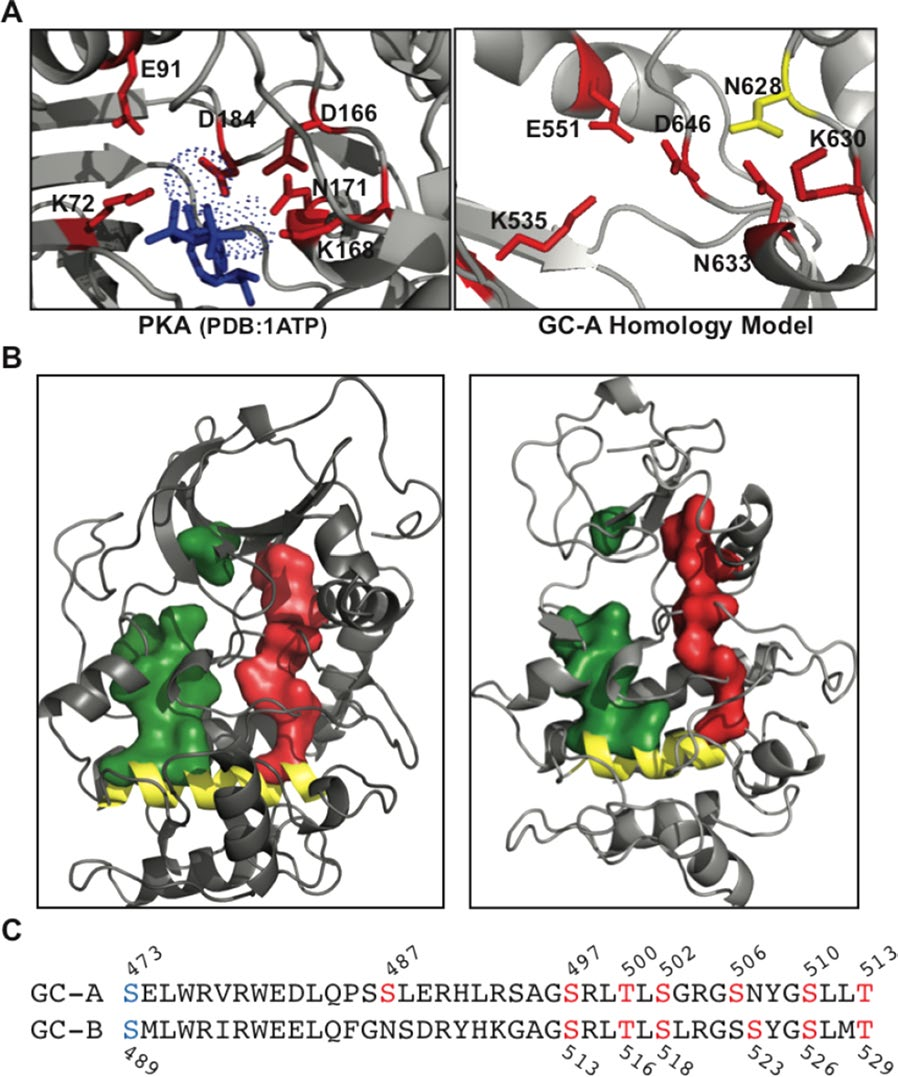
The residues that bind ATP in PKA are conserved in the PKD of GC-A. **a** Structure of the active site of the kinase domain of PKA and the corresponding site in the PKD of GC-A showing conservation of the residues (red) that interact with ATP (blue) in the active site. **b** Structures showing the R-spine (red), the C-spine (green), and the F helix (yellow). **c** The serine and threonine residues mutated in nonphosphorylated or phosphomimetic constructs are indicated with their position numbers

**Acknowledgement:** We thank G. Veglia (University of Minnesota) and L. Jaffe (University of Connecticut Health Center) for the expert discussions and for the constructive comments on this manuscript. Funding: This work was funded by NIH grants T32AR050938 to A.B.E. and NIHR01GM098309 to L.R.P. Grants from the University of Minnesota of Academic Health Center for Faculty Research Development, Office of the Vice President for Research Grants, and the Minnesota Partnership for Biotechnology and Medical Genomics Grant, Fund for Science and Hormone Receptor Fund to L.R.P. also support this work.

**References**

- Kuhn M. Molecular physiology of membrane guanylyl cyclase receptors. Physiol Rev. 2016;96(2):751–804.
- Mishra V, Goel R, Visweswariah SS. The regulatory role of the kinase-homology domain in receptor guanylyl cyclases: nothing ‘pseudo’ about it! Biochem Soc Trans. 2018. **(Epub 2018/11/12)**.
- Antos LK, Potter LR. Adenine nucleotides decrease the apparent Km of endogenous natriuretic peptide receptors for GTP. Am J Physiol Endocrinol Metab. Research Support, N.I.H., Extramural Dec 2007;293(6):E1756–63. **(Epub 2007/09/13)**.
- Robinson JW, Potter LR. Guanylyl cyclases a and B are asymmetric dimers that are allosterically activated by ATP binding to the catalytic domain. Sci Signal. 2012;5(240):ra65. **(Epub 2012/09/06)**.
- Edmund AB, Walseth TF, Levinson NM, Potter LR. The pseudokinase domains of guanylyl cyclase-A and -B allosterically increase the affinity of their catalytic domains for substrate. Sci Signal. 2019;12(566). **(Epub 2019/01/29)**.

## P11 NO-sensitive guanylyl cyclase expression discriminates two types of myofibroblasts in lung fibrosis

### Nils Englert^1^, Annemarie Aue^1^, Achim Schmidtko^2^, Dieter Groneberg^1^, Andreas Friebe^1^

#### ^1^Universität Würzburg, Physiologisches Institut, Würzburg Bavaria, Germany; ^2^Universität Frankfurt, Institut für Pharmakologie und Klinische Pharmazie, Frankfurt Hesse, Germany

##### **Correspondence:** Nils Englert - nils.englert@web.de

*Journal of Translational Medicine* 2019, **17(2):**P11

**Introduction:** Interstitial pulmonary fibrosis (IPF) is a chronic and progressive disease caused by inappropriate wound healing after repetitive lung tissue injury. Fibrosis is accompanied by the formation of myofibroblasts (α-SMA^+^) and excessive production of collagen leading to functionally restricted tissue. Because of limited therapeutic options, median survival of IPF patients is 3–5 years. Anti-fibrotic effects of the NO/cGMP pathway have already been described in other organs such as liver, skin and kidney. Consequently, NO-sensitive guanylyl cyclase (NO-GC) could have a protective effect within lung fibrosis.

**Methods:** Mice deficient in NO-GC (GCKO) allow the investigation of NO-GC-mediated effects. Lung injury was induced by a single intratracheal dose of bleomycin. Lungs were harvested at day 7, 14 or 21 after bleomycin application. Immunohistochemistry was used to identify the NO-GC-expressing cell type(s) and to characterise the fibrotic tissue, e.g. the formation of myofibroblasts. Hydroxyproline assay was performed to quantify the extent of fibrosis.

**Results:** NO-GC is mainly expressed in lung pericytes. Bleomycin treatment leads to destruction of alveolar epithelial cells (AEC), formation of myofibroblasts and increase in collagen content. NO-GC expression discriminates two types of myofibroblasts: (1) intra-alveolar, NO-GC-negative and (2) interstitial, NO-GC-positive myofibroblasts. After bleomycin administration, collagen content in WT and GCKO mice was increased compared to the respective untreated controls at all assessed time points. Furthermore, fibrotic response in GCKO lungs was more pronounced compared to WT.

**Conclusions:** Our results indicate that NO-GC has an anti-fibrotic effect in pulmonary fibrosis. Based on the distinct location and NO-GC expression, interstitial myofibroblasts may constitute ideal therapeutic targets to influence lung fibrosis. In addition, myofibroblasts should not be addressed as a homogeneous target cell type within this disease.

## P12 Can erythrocytes produce biologically active NO?

### Stepan Gambaryan^1,2^

#### ^1^Russian Academy of Sciences, Sechenov Institute of Evolutionary Physiology and Biochemistry, St. Petersburg, Russia; ^2^UNIVERSITÄTSMEDIZIN der Johannes-Gutenberg Universität, Centrum für Thrombose und Hämostase (CTH), Mainz, Germany

##### **Correspondence:** Stepan Gambaryan - gambaryan@klin-biochem.uni-wuerzburg.de

*Journal of Translational Medicine* 2019, **17(2):**P12

**Introduction:** Red blood cells (RBCs) and platelets represent the major cell population in mammalian blood. Functional interactions between RBCs and platelets are known for a long time from clinical observations. The bleeding time is prolonged in patients with anemia independent on their platelet count and could be corrected by RBCs transfusion. Consequently, RBCs transfusion could increase platelet activation and might be dangerous in the treatment of coronary artery diseases. However, in numerous papers nitric oxide (NO) released from RBCs, especially in the presence of nitrite, was shown to inhibit platelet activation. Endogenous NO is a well-known and potent inhibitor of platelet activation and platelet inhibitory effects of NO are mediated solely by sGC activation. Intact platelet sGC is highly sensitive to endogenous NO and even nanomolar NO concentrations are sufficient for cGMP generation and PKG activation. The main aim of our study was to evaluate whether RBCs under different conditions (oxygenated, deoxygenated, in the presence of nitrite) could release biologically active NO which will be involved in inhibition of platelet activation.

**Methods:** For evaluation of platelet sGC activity we used our well-established model based on NO/sGC/cGMP/PKG dependent vasodilator-stimulated phosphoprotein (VASP) phosphorylation in human platelets. In our experiments RBCs were mixed with platelet suspension and sGC activation was assed by VASP phosphorylation.

**Results:** We show that in the whole blood, RBCs prevent NO-mediated inhibition of ADP and TRAP6-induced platelet activation. Likewise, coincubation of RBCs with platelets results in strong inhibition of NO-induced sGC activation. Under hypoxic conditions, incubation of RBCs with NO donor leads to Hb-NO formation which also inhibits sGC activation in platelets.

**Conclusions:** All our experiments are in accordance with clinical observations and clearly show that RBCs, in any conditions, can not be sources of NO, they act only as a strong NO scavengers and prevent NO-mediated platelet inhibition.

**Acknowledgement:** The work was supported by the grant from RFBR № 17-00-00141 (17-00-00139).

## P13 No-GC in pericytes as modulator of skin fibrosis

### Amelie Reigl^1,2^, Florian Groeber-Becker^2^, Andreas Friebe^1^, Dieter Groneberg^1^

#### ^1^University of Wuerzburg, Physiology, Würzburg Bavaria, Germany; ^2^University of Wuerzburg, Tissue Engineering and Regenerative Medicine, Würzburg Bavaria, Germany

##### **Correspondence:** Dieter Groneberg - dieter.groneberg@uni-wuerzburg.de

*Journal of Translational Medicine* 2019, **17(2):**P13

**Introduction:** Disturbed wound healing affects millions of people worldwide. Skin fibrosis is a failure of tissue repair, which is characterized by initial inflammation and appearance of myofibroblasts producing excessive extracellular matrix. There is evidence that nitric oxide (NO) plays an important role in skin fibrosis. NO-sensitive guanylyl cyclase (NO-GC) is the main target for NO. In skin, the exact cell type expressing NO-GC as well its role during wound healing and fibrosis has yet to be identified.

**Methods:** To investigate the function of NO-GC in skin, we used the bleomycin model of skin fibrosis induced by repetitive (21 days) subcutaneous bleomycin injections (0.5 mg/ml) in the neck. Reporter mice were used to lineage trace NO-GC^+^ cells within the course of the fibrotic reaction.

**Results:** We found NO-GC expression in skin pericytes indicated by colocalisation with the pericyte marker PDGFRß; NOGC^+^ pericytes were found to be closely associated with CD31^+^ endothelial cells in the hypodermis of the skin. Tamoxifen-induced expression of the reporter dye tdTomato under the control of SMMHC (smooth muscle myosin heavy chain) promotor was found to colocalize with NO-GC^+^ pericytes. tdTomato-labeled pericytes are spindle shaped and mainly located within a collagen IV matrix which surrounds the adipocytes of the hypodermis. After bleomycin injection, the fibrotic reaction occurred exclusively in the hypodermis. We showed an increase of collagen III and IV and a higher density of fibroblasts. tdTomato labeled pericytes remain in the collagen IV matrix.

**Conclusions:** We assume that NOGC^+^ pericytes constitute an interesting therapeutic target to treat skin fibrosis. In the next step, we want to identify the role of NO-GC using additional promotor-specific lineage tracing of NOGC^+^ cells and knockdown of NO-GC.

## P14 Characterization of cyclic nucleotide phosphodiesterases potentially involved in pain processing

### Tilman Groß^1^, Jonas Petersen^1^, Lea Kennel^1^, Jessica Schlaudraff^2^, Domenico Del Turco^2^, Ruirui Lu^1^, Wiebke Kallenborn-Gerhardt^1^, Achim Schmidtko^1^

#### ^1^Goethe-Universität Frankfurt am Main, Institut für Pharmakologie und Klinische Pharmazie, Frankfurt am Main Hesse, Germany; ^2^Goethe-Universität Frankfurt am Main, Institut für Klinische Neuroanatomie, Frankfurt am Main Hesse, Germany

##### **Correspondence:** Tilman Groß - t.gross@em.uni-frankfurt.de

*Journal of Translational Medicine* 2019, **17(2):**P14

**Introduction:** Accumulating evidence suggests that cGMP essentially contributes to the processing of chronic pain. However, little is known about the cyclic nucleotide phosphodiesterases (PDEs) that regulate the duration and amplitude of cGMP signaling in nociceptive tissues. Twenty-one PDE genes have been identified, which are classified into 11 families (PDE1 to PDE11), each with distinct substrate specificity and tissue distribution. Here, we investigated the distribution of cGMP-hydrolyzing PDEs in the spinal cord and dorsal root ganglia in order to identify PDEs potentially involved in pain processing.

**Methods:** Immunohistochemistry, in situ hybridization, laser microdissection and quantitative RT-PCR experiments were performed to characterize the localization and expressional regulation of cGMP-hydrolyzing PDE isoforms in the mouse spinal cord and dorsal root ganglia. We also analyzed the effects of selective PDE inhibitors in animal models of pain.

**Results:** We found distinct distribution patterns of cGMP-hydrolyzing PDEs in the spinal cord and dorsal root ganglia. Various PDEs are expressed in pain relevant excitatory and inhibitory dorsal horn inter-neurons and C-fiber DRG-neurons. Expression of distinct PDEs is upregulated in the spinal cord after persistent nociceptive stimulation. Moreover, pain behavior was modulated by treatment with PDE inhibitors.

**Conclusions:** Based on these findings we hypothesize that various cGMP-hydrolysing PDEs contribute to pain processing in a specific manner.

**Acknowledgement:** This work was supported by the Deutsche Forschungsgemeinschaft (FOR 2060 project SCHM 2629/3-1).

## P15 Renoprotective effects of soluble guanylate cyclase (sGC) stimulation in hypertensive rats

### Nadine Haase^1,2^, Kristin Kräker^1,2^, Lajos Markó^1,2^, Hendrik Bartolomaeus^1,2^, Damian Brockschnieder^3^, Peter Sandner^3^, Dominik Müller^1,2^, Ralf Dechend^1,4^, Nicola Wilck^1,5^

#### ^1^Experimental and Clinical Research Center (ECRC), a joint cooperation between the Max-Delbrück Center for Molecular Medicine and the Charité Medical Faculty, Berlin Berlin, Germany; ^2^DZHK (German Centre for Cardiovascular Research), partner site Berlin, Berlin Berlin, Germany; ^3^Bayer AG, Drug Discovery, Berlin Berlin, Germany; ^4^HELIOS-Klinikum, Berlin Berlin, Germany; ^5^Charité – Universitätsmedizin Berlin, Med. Klinik m. S. Nephrologie und Internistische Intensivmedizin CCM/CVK, Berlin Berlin, Germany

##### **Correspondence:** Nadine Haase - nadine.haase@mdc-berlin.de

*Journal of Translational Medicine* 2019, **17(2):**P15

**Introduction:** Renal damage is a hallmark of persistent arterial hypertension. Current anti-hypertensive pharmacotherapies aim to protect from renal damage. However, this goal is not reached in many cases since both, pressure-dependent and -independent mechanisms can contribute to renal damage. Soluble guanylate cyclase (sGC) stimulation addresses an important signalling pathway in the cardiovascular and cardiorenal system. We have recently published that sGC stimulation using BAY 41-8543 significantly reduces blood pressure and protects from experimental heart failure with preserved ejection fraction in rats. Here we aimed to investigate the effect of BAY 41-8543 on renal damage in hypertensive rats. To test blood pressure dependent and blood pressure independent effects, we used two different doses of BAY 41-8543.

**Methods:** We used 4-week-old male double transgenic rats (dTGR) expressing both human renin and angiotensinogen genes. dTGR progressively develop hypertension and renal damage, leading to a high mortality at 7 weeks of age. We treated dTGR with either 0.3 mg/kg/d, 3 mg/kg/d BAY 41-8543 or vehicle daily for 3 weeks by oral gavage. Age-matched vehicle-treated Sprague–Dawley (SD) rats served as healthy controls.

**Results:** Both doses improved the survival at week 7 as compared to vehicle. The higher dose reduced mean arterial blood pressure significantly as compared to vehicle, whereas the lower dose did not. Still, the lower dose significantly augmented cGMP serum levels. High dose-treated dTRG were protected from albuminuria throughout the experiment, whereas the low dose reduced albuminuria only in weeks 5–6. Cystatin C serum levels were significantly reduced after 3 mg/kg/d BAY 41-8543 treatment compared to vehicle, indicating preservation of glomerular filtration. Kidney injury marker NGAL was significantly reduced in the serum of dTGR treated with 3 mg/kg/d BAY 41-8543. Anti-CD68 immunofluorescence of kidney sections indicated significantly less immune cell infiltration in dTGR treated with the higher dose. Further histologic characterization of renal damage markers was undertaken.

**Conclusions:** The sGC stimulator BAY 41-8543 can dose-dependently protect from hypertensive induced renal damage. The magnitude of the effect of BAY 41-8543 was higher in a blood pressure lowering dose but also a blood pressure neutral dose was beneficial.

## P16 Investigating the protective role of the NO/sGC/PKG pathway in diabetic nephropathy in vivo and in vitro

### Manuela Harloff, Jens Schloßmann

#### University of Regensburg, Department of Pharmacology and Toxicology, Regensburg, Germany

##### **Correspondence:** Manuela Harloff - manuela.harloff@ur.de

*Journal of Translational Medicine* 2019, **17(2):**P16

**Introduction:** Diabetic nephropathy (DN) is a severe microvascular complication of a persisting Diabetes mellitus. DN is the leading cause of end-stage renal disease worldwide, which requires dialysis and renal replacement therapy. A specific therapy does not exist so far because underlying mechanisms are still not completely understood. Previous studies showed that treatments with sGC-activators prevent kidney damages [1, 2]. In this work we want to investigate the role of the NO/sGC/PKG pathway in the development of diabetic nephropathy using the sGC-activator Cinaciguat as a potential therapeutic approach.

**Methods:** For the in vivo studies, we established a mouse model for DN using wild type and eNOS-KO mice [3]. Diabetes mellitus was induced by streptozotocin injections (50 mg/kg/d; i.p.) on 5 consecutive days. After 12 weeks the mice developed DN-typical kidney damages. Half of these mice received Cinaciguat (3 mg/kg/d, p.o.) in the last 4 weeks.

For further in vitro analyses we used primary murine mesangial cells isolated from mouse kidneys. To simulate the blood glucose levels in diabetes, the cells were incubated with normal glucose (8 mM d-Glucose, NG) and high glucose (25 mM d-Glucose, HG) conditions for 48 h. To activate the NO/sGC/PKG signaling, cells under HG conditions were additionally treated with 1 mM 8-Br-cGMP or with 20 µM cinaciguat.

**Results:** After induction of diabetes, the mice developed typical symptoms like elevated blood glucose levels, glucosuria or higher blood pressure. Treatment with cinaciguat appeared to have no influence on these symptoms.

After 12 weeks of diabetes changes in the kidneys can be detected. Diabetic kidneys showed an increased expression of Collagen IV and α-SMA associated with fibrotic processes. In kidneys from cinaciguat treated mice, no increase of these markers were detectable.

Thrombospondin-1 (TSP-1), an important regulator of transforming growth factor (TGF)-β, plays a critical role in the development of kidney damage [4]. TSP-1 gene expression was upregulated in diabetic kidneys, whereas mice treated with cinaciguat showed significantly lower levels of TSP-1 mRNA.

In mesangial cells HG conditions led to a higher TSP-1 expression. Further incubation of the cells with 8-Br-cGMP or Cinaciguat retained the protein expression levels equally to normal glucose conditions. Moreover the expression of the glucose transporter GLUT-1 was increased under HG conditions conceivably induced by higher TSP-1 levels. Activation of PKG with 8-Br-cGMP inhibited the higher GLUT-1 expression.

**Conclusions:** Our first results show that the activation of the NO/sGC/PKG pathway with cinaciguat has protective effects on DN and might be an appropriate new therapy option.

**References**

- Schinner E, Schramm A, Kees F, et al. The cyclic GMP-dependent protein kinase Ialpha suppresses kidney fibrosis. Kidney Int. 2013;84(6):1198–206.
- Boustany-Kari CM, Harrison PC, Chen H et al. A soluble guanylate cyclase activator inhibits the progression of diabetic nephropathy in the ZSF1 rat. J Pharmacol Exp Ther. 2016;356(3):712–9.
- Nakagawa T, Sato W, Glushakova O, et al. Diabetic endothelial nitric oxide synthase knockout mice develop advanced diabetic nephropathy. J Am Soc Nephrol. 2007;18(2):539–50.
- Lu A, Miao M, Schoeb TR, et al. Blockade of TSP1-dependent TGF-beta activity reduces renal injury and proteinuria in a murine model of diabetic nephropathy. Am J Pathol. 2011;178(6):2573–86.

## P17 Chronic stimulation of the soluble guanylate cyclase (sGC) with vericiguat improves outcome in an HFpEF-like (heart failure with preserved ejection fraction) rat model

### Christina Jochem, Mira Pavkovic, Nina Scheerer, Elke Hartmann, Peter Sandner, Ilka Mathar

#### Bayer AG, Research and Development, Wuppertal North Rhine-Westphalia, Germany

##### **Correspondence:** Christina Jochem - christina.jochem@bayer.com

*Journal of Translational Medicine* 2019, **17(2):**P17

**Introduction:** Heart failure is one of the leading causes of death worldwide and until now, the pathophysiology of HFpEF is poorly understood and many treatment approaches failed. HFpEF represents roughly 50% of all HF patients and is characterized by impaired diastolic function, associated amongst with interstitial fibrosis, LV remodeling/hypertrophy and atrial enlargement.

For the development of novel therapies and to understand the different underlying pathophysiological mechanisms, more predictive animal models are needed.

**Methods:** Since hypertension and low nitric oxide (NO) level are associated with HF and HFpEF, we treated hypertensive, renin-transgenic (RenTG) rats with L-NAME, mimicking hypertension and low NO-conditions by blockade of NO-synthesis. This rodent model is characterized by hypertension-induced HF and shows also features of an HFpEF-like phenotype. We chronically treated these rats with either placebo, or with the soluble guanylate cyclase stimulator vericiguat (0.1, 0.3, 1, 3 mg/kg QD).

**Results:** The RenTG placebo treated rats, in comparison to healthy controls (n > 10/group), developed an hypertension-induced end-organ damage and a HFpEF phenotype, including pulmonary congestion (3.4 ± 0.08 vs 3.8 ± 0>06 g/kg BW, p < 0.01), left ventricular (1.85 ± 0.05 vs 3.0 ± 0.07 g/kg BW, p < 0.01) and atrial hypertrophy (0.08 ± 0.009 vs 0.11 ± 0.006 g/kg BW, p < 0,05) and prolonged left ventricular isovolumetric relaxation constant TAU (0.012 ± 0.0004 vs 0.017 ± 0.0006s, p < 0.05) and preserved LV systolic function (EF, 58 ± 2.1 vs 55 ± 1.5%, ns). Chronic treatment with Vericiguat, showed significant improvement in cardiac function at doses where blood pressure was unchanged. In addition, there was a significant, dose-dependent reduction of mortality (67% vs 30% mortality in the control vs 0.3 mg/kg vericiguat group).

**Conclusions:** The L-NAME-treated, hypertensive RenTG rat represents a rodent model with HFpEF-like features. Chronic stimulation of sGC with Vericiguat improves cardiac function suggesting a potential treatment opportunity for HFpEF.

**Acknowledgement:** Funding was provided by Bayer AG and Merck Sharp & Dohme Corp., a subsidiary of Merck & Co., Inc., Kenilworth, NJ, USA.

## P18 Development of a soluble guanylate cyclase radioligand binding assay using [^3^H]-praliciguat

### Marco M. Kessler^1^, Deborah F. Dodge^2^, Daniel P. Zimmer^2^, Joon Jung^2^, Paul A. Renhowe^2^, John R. Hadcock^2^

#### ^1^Ironwood Pharmaceuticals, Cambridge Massachusetts, US; ^2^Cyclerion Therapeutics, Cambridge Massachusetts, US

##### **Correspondence:** Marco M. Kessler - mkessler@ironwoodpharma.com

*Journal of Translational Medicine* 2019, **17(2):**P18

**Introduction:** Soluble guanylate cyclase (sGC) stimulators are a class of small molecule agonists that bind to sGC and act in synergy with nitric oxide (NO) to increase production of cGMP from GTP. sGC stimulators have been characterized for their ability to stimulate cGMP production in cellular and purified enzyme assays, but a robust assay for determining relative binding affinities of sGC stimulators has been lacking. Praliciguat is an investigational, oral, once-daily sGC stimulator.

**Methods:** We developed a binding assay employing [^3^H]-praliciguat and size-exclusion chromatography to analyze the binding of praliciguat to purified human recombinant sGC. We also identified cofactors required for binding and used the assay to explore the relative binding affinities of other sGC stimulator compounds.

**Results:** Binding of [^3^H]-praliciguat to recombinant sGC was saturable with a *K*_D_ of 9 nM, a Hill coefficient of 1.1, and a linear Scatchard plot, indicating no cooperativity of binding and a single binding site. Binding of [^3^H]-praliciguat to sGC required NO and was optimal in the presence of the substrate GTP or a non-hydrolyzable GTP analog. Under the conditions used for binding, sGC was fully active with a cGMP-forming activity of 10.8 µmol cGMP/min/mg. A competitive radioligand binding assay using [^3^H]-praliciguat in the presence of increasing concentrations of unlabeled praliciguat was used to determine the relative binding affinity of praliciguat for sGC. This resulted in an observed K*i* of 3.64 ± 0.26 nM. The binding assay was used to determine the relative binding affinities of other sGC stimulators with observed K*i* values from 3.6 to 905 nM. The affinities (Ki) of these compounds in the sGC binding assay correlated with their potencies (EC_50_) in the cGMP formation enzyme assay. The radioligand binding assay using [^3^H]-praliciguat and sGC, followed by a challenge with excess unlabeled praliciguat was also used to estimate its dissociation rate. The k_off_ rate was 0.056 min^−1^ (t_1/2_ = 12.3 min) and the calculated association rate (k_on_) was 6.2 × 10^6^ M^−1^ min^−1^.

**Conclusions:** Praliciguat bound to sGC with high affinity and appeared to bind to a single binding site on sGC with no observed cooperativity. In this assay, praliciguat binding to sGC required NO and was optimal in the presence of the substrate, GTP. These observations are consistent with the role of praliciguat as an allosteric modulator of sGC potentiating the activity of NO. The assay can be used to explore binding kinetics and may be a useful tool for identification and characterization of sGC stimulators with novel binding and kinetic properties.

## P19 Suppression of tumorigenicity 2 as a biomarker in pulmonary arterial hypertension and its association with REVEAL risk score in riociguat-treated patients in the RESPITE study

### James R Klinger^1^, Raymond L Benza^2^, Hossein-Ardeschir Ghofrani^3^, Pavel Jansa^4^, Ekkehard Grünig^5^, Dario Vizza^6^, Christian Meier^7^, Dennis Busse^8^, Marius M Hoeper^9^

#### ^1^Division of Pulmonary, Sleep, and Critical Care Medicine, Rhode Island Hospital, Alpert Medical School of Brown University, Providence Rhode Island, US; ^2^The Cardiovascular Institute, Allegheny General Hospital, Pittsburg Pennsylvania, US; ^3^University of Giessen and Marburg Lung Center (UGMLC), member of the German Center for Lung Research (DZL), Giessen Hesse, Germany; ^4^Clinical Department of Cardiology and Angiology, 1st Faculty of Medicine, Charles University, Prague, Czech Republic; ^5^Centre for Pulmonary Hypertension, Thorax Clinic at the University Hospital, Heidelberg Baden-Württemberg, Germany; ^6^Pulmonary Hypertension Unit, Department of Cardiovascular and Respiratory Disease, La Sapienza University of Rome, Rome, Italy; ^7^Bayer AG, Berlin, Germany; ^8^Chrestos Concept GmbH & Co. KG, Essen North Rhine-Westphalia, Germany; ^9^Clinic for Respiratory Medicine, Hannover Medical School, member of the Germany Center for Lung Research (DZL), Hannover Lower Saxony, Germany

##### **Correspondence:** James R Klinger - james_klinger@brown.edu

*Journal of Translational Medicine* 2019, **17(2):**P19

**Introduction:** Suppression of tumorigenicity 2 (ST-2) is a marker of cardiac remodeling and fibrosis that correlates with disease severity in patients with pulmonary arterial hypertension (PAH), and is thought to be a predictor of clinical worsening. We performed a post hoc analysis assessing ST-2 as a potential biomarker of likelihood of response in patients switching from phosphodiesterase type 5 inhibitors (PDE5i) to riociguat, and the association of ST-2 with REVEAL risk score (RRS) in the RESPITE study.

**Methods:** RESPITE was a 24-week, open-label, uncontrolled, Phase IIIb study in PAH patients with an insufficient response to PDE5i. Patients underwent a PDE5i washout period before receiving riociguat individually adjusted up to 2.5 mg three times daily. Responder status at Week 24 was defined as freedom from clinical worsening, improvement of ≥ 30 m in 6-min walking distance, and World Health Organization functional class I/II. Data were analyzed descriptively as mean ± SD. Statistical significance was assessed using *t* tests.

**Results:** At baseline, ST-2 was 21 ± 15 ng/mL in the overall population (n = 53), and responders (n = 16) had lower ST-2 levels than non-responders (n = 35) (17 ± 8 vs 23 ± 17 ng/mL). When patients were grouped according to RRS, ST-2 levels were lowest in low-risk patients (RRS 0–6; n = 24) at baseline (15 ± 7 ng/mL) and Week 24 (16 ± 8 ng/mL) and highest in high-risk patients (RRS 9–13; n = 17) at baseline (33 ± 20 ng/mL) and Week 24 (25 ± 12 ng/mL). There was a significant positive correlation between ST-2 at Week 0 and RRS at Week 0 (*r *= 0.71; p < 0.0001) and at Week 24 (*r *= 0.61; p < 0.0001). From baseline to Week 24, ST-2 levels were reduced in the overall population (− 2.3 ng/mL, p = 0.095), responders (− 2.1 ng/mL, p = 0.094), and non-responders (− 2.4 ng/mL, p = 0.217). Reductions were also seen in RRS from Week 0 to Week 24 in the overall population (− 1.3), responders (− 3.3, p < 0.0001), and non-responders (− 0.8, p = 0.0063). Although minor changes in ST-2 levels were seen from baseline to Week 24 in the low- and intermediate-risk groups (+ 0.9 and + 0.2 ng/mL, respectively), ST-2 had decreased by –10 ng/mL in the high-risk group at Week 24.

**Conclusions:** ST-2 may have potential as a new biomarker to identify patients who may be at higher risk of 1-year mortality at treatment commencement and who may be more likely to respond to riociguat.

## P20 Soluble guanylate cyclase agonists induce bronchodilation in human small airways

### Cynthia Koziol-White^1^, Arnab Ghosh^2^, Peter Sandner^3^, Serpil Erzurum^2^, Dennis Stuehr^2^, Rey Panettieri^1^

#### ^1^Rutgers University, Institute for Translational Medicine & Science, New Brunswick New Jersey, US; ^2^Cleveland Clinic, LRI, Dept. Inflammation & Immunity, Cleveland Ohio, US; ^3^Bayer, Pharmaceuticals R & D, Wuppertal, Germany

##### **Correspondence:** Cynthia Koziol-White - cjk167@rbhs.rutgers.edu

*Journal of Translational Medicine* 2019, **17(2):**P20

**Introduction:** Despite improvements in the treatment of airway inflammation in asthma, there has been little progress in the development of novel classes of bronchodilators for nearly 50 years. Most commercial bronchodilators work through generation of cAMP, and show diminished efficacy in the presence of Th2 lung inflammation, and when patients develop β_2_AR desensitization. Although the NO-sGC-cGMP signaling pathway is known to evoke vascular smooth muscle relaxation, its role in mediating airway smooth muscle relaxation remains controversial. Recently, direct-acting sGC agonists that bypass NO generation have been developed and thus offer a new opportunity to assess the pathway in airway relaxation. The sGC agonists, BAY 41-2272 (BAY 41) and BAY 60-2770 (BAY 60), stimulate the heme-containing sGC or directly activate the heme-free form of sGC, respectively. In order to ascertain their potential value as bronchodilators, we compared BAY 41 and BAY 60 against a commercial beta agonist drug (Formoterol) in their abilities to reverse contractile agonist-induced airway bronchoconstriction in human lung samples.

**Methods:** Precision cut lung slices (PCLS) were prepared from lungs of healthy human donors. PCLS bronchioles were precontracted to 70–80% maximal with carbachol, and were then titrated with BAY 41, BAY 60, or formoterol at increasing doses. Bronchiole diameter was measured and quantified after each dose using microscopy and imaging analysis. In some cases the PCLS were incubated overnight with formoterol (100 nM) to induce β_2_AR desensitization, prior to the titrations. Production of cGMP or cAMP by the PCLS was measured using commercial kits.

**Results:** BAY 41-2272 and BAY 60-2770 reversed the carbachol-induced bronchoconstriction in human PCLS at similar concentrations and to comparable maximal levels as did formoterol (Fig. 1).

**Fig. 1:**
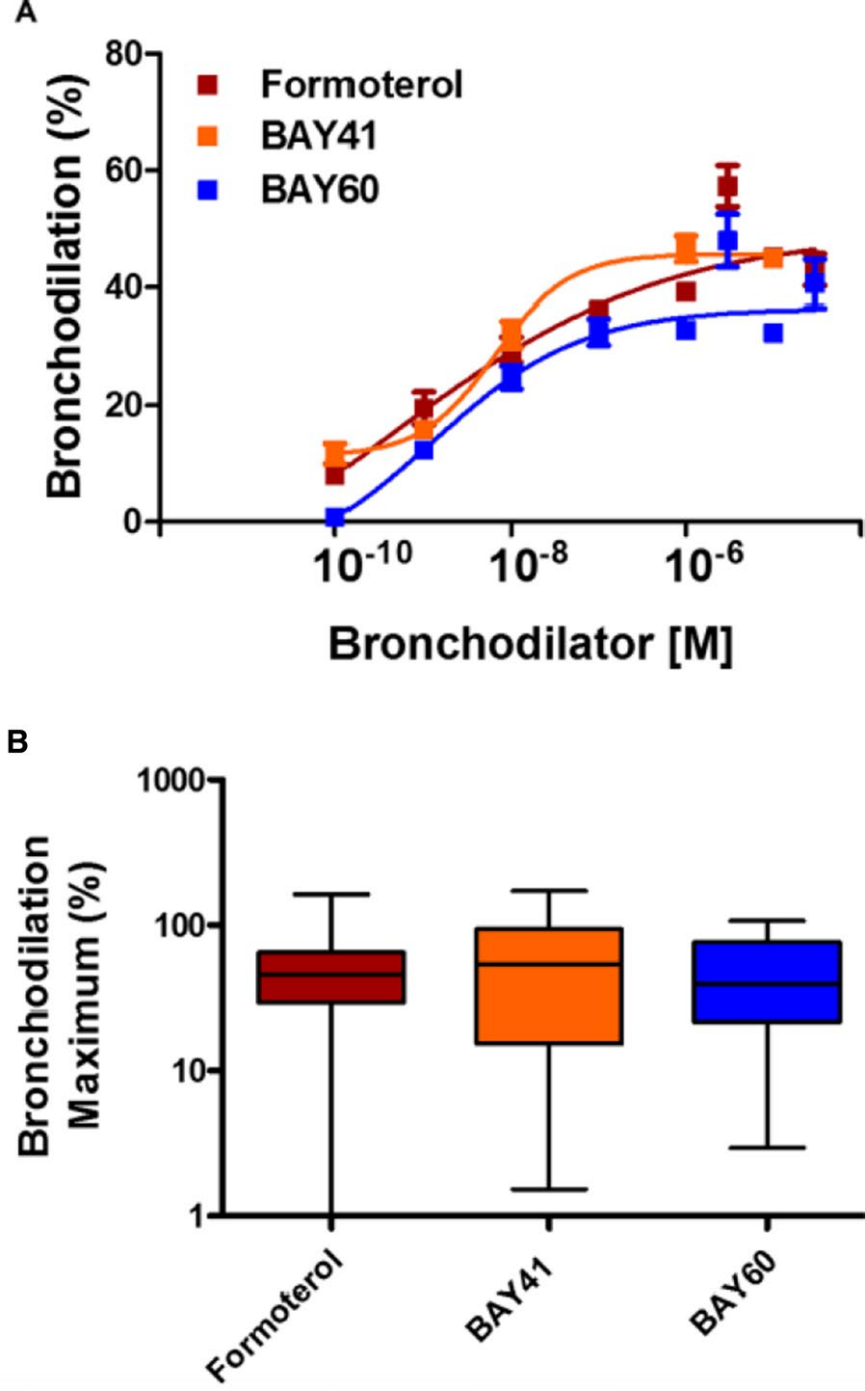
sGC agonists are effective as formoterol in bronchodilating human lung slices. Small airways in lung slices were contracted with carbachol followed by addition of the indicated compounds and image collection and processing to determine bronchiole lumen area, expressed as % compared to baseline. **a** Concentration response curves (100 pM–30 µM) toward formoterol, BAY 41, or BAY 60. **b** Maximal amount of bronchodilation induced by each bronchodilator. Data derived from n = 15 donor lungs, showing mean ± SEM with 18–27 slices tested per condition

Both BAY drugs were effective bronchodilators in human PCLS that had become desensitized to formoterol.

The sGC or β_2_AR agonists induced distinct cyclic nucleotide accumulation (cGMP vs cAMP) in the human PCLS.

**Conclusions:** Activating the sGC-cGMP pathway with sGC agonists was equi-effective as activating the β2AR-cAMP pathway with formoterol in reversing a carbachol-induced bronchoconstriction in the human airway. The sGC agonists still bronchodilated β2AR-desensitized airways. sGC stimulator and activator (BAY 41 & BAY 60) were similarly effective, indicating that both heme-replete and heme-deficient/oxidized sGC are present in the human airway samples and could be targeted for inducing bronchodilation. This should enable future studies to test whether sGC-targeted drugs alone or in combination can serve as effective bronchodilators in patients with asthma and COPD.

**Acknowledgement:** Supported by NIH grants HL081064 and HL114471. We thank William Jester, Gaoyuan Cao, and Brian Deeney for expert technical assistance.

## P21 CNP-induced cGMP signaling in the gastrointestinal tract

### Michael S. Krämer, Sarah Merz, Robert Feil, Hannes Schmidt

#### University of Tübingen, Interfaculty Institute of Biochemistry, Tübingen Baden-Württemberg, Germany

##### **Correspondence:** Michael S. Krämer - michael.kraemer@uni-tuebingen.de

*Journal of Translational Medicine* 2019, **17(2):**P21

**Introduction:** The peptide hormone C-type natriuretic peptide (CNP) and its receptor guanylate cyclase B (GC-B) are critically involved in many important physiological processes such as bone growth, oocyte maturation, homeostasis of arterial blood pressure and sensory axon bifurcation. Furthermore, mice with a global loss of GC-B activity suffer from severe gastrointestinal malfunction which probably contributes to the premature death of these animals. Previous reports suggested that CNP-induced relaxation of smooth muscle cells is involved in the regulation of pyloric and colorectal motility. However, the identity of CNP and GC-B expressing cells underlying CNP’s effects in the gastrointestinal tract needs to be resolved.

**Methods:** We used reporter mouse lines generated by our lab for the characterization of CNP and GC-B expressing cells in the gastrointestinal tract. Expression mapping using X-Gal staining of whole mount tissue from CNP- and GC-B-lacZ reporter lines (*CNP*^*wt/lacZ*^ and *GC*-*B*^*wt/lacZ*^) was followed by immunofluorescent double labeling of sections with anti-β-galactosidase and cell type-specific marker antibodies. In a second approach, we used our mouse line expressing tamoxifen-inducible CreERT2 under control of the GC-B promotor in combination with a conditional tdTomato-based reporter line (*GC*-*B*^*wt/CreERT2*^*::R26*^*wt/LSL*−*tdTomato*^) to further elucidate the identity of GC-B expressing cell populations.

**Results:** A comprehensive analysis of 13 gastrointestinal compartments demonstrated that both CNP and GC-B are expressed throughout the gastrointestinal tract. While high densities of CNP-expressing cells were detected in the esophagus, pylorus, jejunum/ileum and colon, GC-B expression was observed to be especially high in the esophagus, pylorus, colon, and mesenteric lymph nodes. Beginning to determine the cellular identity of CNP and GC-B expressing cells in the colon we found that CNP and GC-B are expressed in neuronal subpopulations of the myenteric plexus in a mostly non-overlapping manner (less than 5%). Co-labeling with an antibody specific for neuronal nitric oxide synthase demonstrated that approximately half of the CNP as well as GC-B positive cells are nitrergic myenteric neurons.

**Conclusions:** Overall, our data point towards an involvement of the CNP/GC-B system in the modulation of neuronal signaling processes which control colonic motility. This will be further investigated by cGMP imaging in cGMP sensor mice and contraction studies of mouse lines with conditionally inactivated CNP and GC-B in colonic neurons.

**Acknowledgement:** This work was supported by DFG grants FOR 2060 (FE 438/5-1 and FE 438/6-1 to RF, and SCHM 2371/1 to HS).

## P22 Comparison of different measurement methods of cGMP

### Marcel Kremser^1^, Steffen Winkler^1^, Frank Eitner^1,2^, Agnès Bénardeau^1^, Jan Kraehling^1^

#### ^1^Bayer AG, Pharma Research Center - Cardiovascular Research, Wuppertal North Rhine-Westphalia, Germany; ^2^RWTH Aachen University, Division of Nephrology and Clinical Immunology, Aachen North Rhine-Westphalia, Germany

##### **Correspondence:** Marcel Kremser - marcelkremser@yahoo.de

*Journal of Translational Medicine* 2019, **17(2):**P22

**Introduction:** The sGC/cGMP pathway is of utmost importance to cardiovascular health. Therefore, the precise measurement of cGMP concentration is crucial to assess the activity of this central signaling cascade and to quantify potential drug effects. Various methods to measure cGMP including, HPLC, ELISA (R&D Systems) and Homogeneous Time Resolved Fluorescence (HTRF, Cisbio) have been developed but were not systematically compared yet.

**Methods:** A direct comparison between commercially available ELISA and HTRF and an in-house HPLC method have been performed using cGMP-spiked buffers, cGMP derived from purified sGC and from overexpressing CHO cells, as well as urine samples and tissue samples from mice treated with vehicle and sGC activator BAY 602770.

**Results:** The direct comparison of these three methods revealed that measuring cGMP using HTRF results in smaller standard deviations and sensitivity in the nM range, while consuming less sample volume, taking less time and being more cost-effective compared to the ELISA used. HPLC proved to be the most precise method, but being only applicable in a µM range, a concentration not suitable for in vivo studies (e.g. urine: ~ 100 nM/plasma: ~ 50 nM).

**Conclusions:** HTRF proved to be suitable and reliable to measure cGMP in biological fluids and tissues. Further analysis of various samples from in vivo studies will help to support the use of HTRF-based cGMP measurements in sGC and PDE related research activities (Fig. 1).

**Fig. 1:**
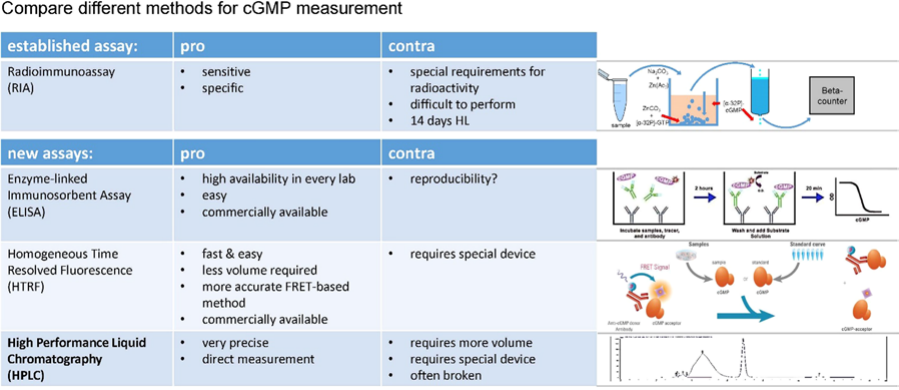
Comparison of different measurement methods of cGMP. Graphical abstract depicting the three different methods used to compare their suitability for cGMP measurements

**Acknowledgement:** Elena Kristin Kretschmer and Paul Ndiaye.

## P23 The NO/cGMP/PKG and cAMP/PKA pathways target the cell cycle regulatory system MASTL-ENSA/ARPP19- PP2A in human platelets

### Elena J. Kumm^1^, Oliver Pagel^2^, Stephanie Makhoul^1^, René Zahedi^2,3^, Albert Smolenski^4^, Stepan Gambaryan^5^, Ulrich Walter^1^, Kerstin Jurk^1^

#### ^1^University Medical Center of the Johannes Gutenberg University Mainz, Center for Thrombosis and Hemostasis (CTH), Mainz, Germany; ^2^Leibniz-Institut für Analytische Wisschenschaften, ISAS - e.V., Dortmund, Germany; ^3^Jewish General Hospital, Proteomics Centre, Lady Davis Institute, Montréal, Canada; ^4^UCD Conway Institute, UCD School of Medicine and Medical Science, University College Dublin, Dublin, Ireland; ^5^Sechenov Institute of Evolutionary Physiology and Biochemistry, Russian Academy of Sciences, St. Petersburg, Russia

##### **Correspondence:** Elena J. Kumm - elena.kumm@unimedizin-mainz.de

*Journal of Translational Medicine* 2019, **17(2):**P23

**Introduction:** Microtubule-associated Ser/Thr kinase-like (MASTL) kinases control the cell cycle from yeast to man by phosphorylating (S67) α-endosulfine (ENSA) or (S62) ARPP19, which converts them into strong inhibitors of protein phosphatase 2A (PP2A). The MASTL-ENSA-PP2A pathway controls the phosphorylation of multiple proteins and enables mitotic entry [1] but is not known in anucleate platelets. ENSA/ARPP19 were detected in our human platelet proteomic/phospho-proteomic studies with S109 ENSA/S104 ARPP19 phosphorylation in response to cAMP-/cGMP-elevating platelet inhibitors [2, 3]. Here, the cell cycle regulatory pathway MASTL-ENSA/ARPP19-PP2A was investigated in human platelets.

**Methods:** ENSA-S67/S109 or ARPP19-S62/S104 phosphorylation was analysed in intact platelets by phosphoproteomics [2] or with purified kinases (PKG, PKA, MASTL) and recombinant proteins/phosphosite-mutants by immunoblotting. Platelet PP2A activity and its regulation by recombinant pS67-ENSA/pS62-ARPP19 was investigated by PP2A-specific phosphatase assays, functional effects of PP2A inhibition on platelets by aggregometry.

**Results:** ENSA, ARPP19 and PP2A, but not the classical MASTL, were abundantly expressed in human platelets. cAMP-/cGMP-elevating platelet inhibitors increased S109 ENSA/S104 ARPP19 phosphorylation and decreased S67 ENSA/S62 ARPP19 phosphorylation. Specific ENSA-S109/ARPP19-S104 (by PKG and PKA) as well as ENSA-S67/ARPP19-S62 (by MASTL) phosphorylation was confirmed with recombinant proteins. These phosphorylation sites were also detected upon phosphatase PP1/PP2A inhibition with the toxin okadaic acid (OA) for endogenous ENSA, and recombinant wild-type ENSA/ARPP19 in intact platelets and lysates. This demonstrated a specific MASTL-like kinase activity in human platelets. A broad spectrum of platelet PP2A holoenzymes was detected by proteomics. pS67-HisENSA and pS62-GST-ARPP19 reduced platelet PP2A activity of about 20% in platelet lysates compared to controls, stronger PP2A inhibition was observed with specific protein substrates. 1 nM OA completely inhibited platelet PP2A activity. Phosphorylation of PP2A substrates (ENSA, VASP, p38) was increased in low-dose OA-treated human platelets and lysates, associated with inhibition of thrombin-induced platelet aggregation.

**Conclusions:** Both ARPP19 and ENSA, part of the cell cycle machinery, are substrates of the NO/cGMP/PKG and cAMP/PKA pathways (S109/S104) and a MASTL-like kinase (S67/S62) in human platelets. The [MASTL-like]–ENSA/ARPP19–PP2A pathway including the potent PP2A inhibition by pS62/pS67 ARPP19/ENSA is regulated in these non-dividing cells by multisite phosphorylation which may control essential platelet functions via PP2A substrates.

**Acknowledgement**: The project is supported by the Deutsche Forschungsgemeinschaft (DFG; JU 2735/2-1) and the Federal Ministry of Education and Research (BMBF 01EO1503).

**References**

1. Castro A, Lorca T. J Cell Sci. 2018.
2. Beck F, et al. Blood. 2017.
3. Makhoul S, et al. Nitric oxide. 2018.

## P24 Cysteine-rich LIM-only protein 4 (CRP4) and its role in vascular smooth muscle cell function and vascular disease

### Natalie Längst^1^, Julia Adler^1^, Andreas Peter^2^, Karsten Boldt^3^, Robert Lukowski^1^

#### ^1^University of Tübingen, Experimental Pharmacology, Department of Pharmacology, Toxicology and Clinical Pharmacy, Institute of Pharmacy, Tübingen Baden-Württemberg, Germany; ^2^University Hospital Tübingen, Institute for Clinical Chemistry and Pathobiochemistry, Department for Diagnostic Laboratory Medicine, Tübingen Baden-Württemberg, Germany; ^3^University of Tübingen, Molecular Biology of Retinal Degenerations, Institute for Ophthalmic Research, Tübingen Baden-Württemberg, Germany

##### **Correspondence:** Natalie Längst - natalie.zinn@uni-tuebingen.de

*Journal of Translational Medicine* 2019, **17(2):**P24

**Introduction:** LIM domain proteins play an essential role for normal cardiac development and function. Studying the hearts of CRP4 deficient mice, we demonstrated a direct interaction between CRP4 and cGMP-dependent protein kinase I (cGKI) in angiotensin II stressed hearts and, in consequence, lack of CRP4 exaggerated the angiotensin II provoked cardiac remodelling [1]. Although, CRP4 has been identified as a cGMP/cGKI dependent modulator of the vascular smooth muscle cell (VSMC)-specific gene program [2, 3], which is usually perturbed during vessel injury when “healthy” VSMCs switch from a contractile to a synthetic cell phenotype, the vascular functions of CRP4 in both health and disease remain largely elusive so far.

**Methods:** Primary and highly passaged VSMCs were established from the aorta of CRP4 mutant (CRP4-KO) and littermate control siblings (CRP4-WT). The localization of CRP4 as well as typical VSMC proteins were studied by immunohistochemical methods. Proteomic data were generated in order to identify CRP4-regulated proteins. Because proliferation and cell migration are hallmarks of phenotypic diversity in VSMCs, we studied whether these cell behaviours were dependent on cGMP/CRP4. Blood pressure of CRP4-WT/-KO mice was assessed at baseline and upon challenge with different cGMP-elevating agents utilizing a radiotelemetric system. Finally, we are presently investigating CRP4 and ApoE double-mutant mice fed a Western diet for atherosclerotic lesion formation via *en*-*face* oil red staining and CRP4-dependent changes in the lipid panel in plasma.

**Results:** CRP4 is predominantly localized to the cytoplasm and the perinuclear region of VSMCs. Yet, distinct changes regarding the expression profile of typical VSMC genes and proteins became apparent in contractile versus synthetic VSMCs. Accordingly, the proliferative and migratory behaviours differed in a CRP4-dependent manner between primary and cultivated VSMCs. In the absence and presence of cGMP-elevating compounds we find evidence for higher cGMP levels in synthetic VSMCs lacking CRP4, which may at least partly explain the reduced blood pressure observed in global CRP4-KO mice. Our ongoing analyses of a recently established CRP4 and ApoE double-mutant mouse model should further validate the role of this LIM-only protein in VSMCs within atherosclerotic lesions in vivo.

**Conclusions:** CRP4 controls blood pressure under physiological conditions and it serves different functions in “healthy” VSMCs and during phenotype modulation of VSMCs in disease. Our combined ex vivo and in vivo analysis of CRP4-deficient VSMCs and mouse models imply that CRP4 may be a cGMP-dependent modifier of vascular diseases associated with hypertension.

**References**

1. Straubinger J, Boldt K, Kuret A, Deng L, Krattenmacher D, Bork N, Desch M, Feil R, Feil S, Nemer M, Ueffing M, Ruth P, Just S, Lukowski R. Amplified pathogenic actions of angiotensin II in cysteine-rich LIM-only protein 4-negative mouse hearts. FASEB J. 2017;31:1620–38.
2. Zhang T, Zhuang S, Casteel DE, Looney DJ, Boss GR, Pilz RB. A cysteine-rich LIM-only protein mediates regulation of smooth muscle-specific gene expression by cGMP-dependent protein kinase. J Biol Chem. 2007;282:33367–80.
3. Huber A, Neuhuber WL, Klugbauer N, Ruth P, Allescher H–D. Cysteine-rich protein 2, a novel substrate for cGMP kinase I in enteric neurons and intestinal smooth muscle. J Biol Chem. 2000;275:5504–11.

## P25 Deficiency of the NO-sensitive guanylyl cyclase 1 sensitizes mice to COPD-related changes

### Malte Verheyen^1^, Marcus Peters^2^, Michelle Puschkarow^2^, Albrecht Bufe^2^, Doris Koesling^1^, Evanthia Mergia^1^

#### ^1^Institute of Pharamacology Ruhr-University Bochum, Bochum North Rhine-Westphalia, Germany; ^2^Department of experimental Pneumology, Bochum North Rhine-Westphalia, Germany

##### **Correspondence:** Evanthia Mergia - mergia@outlook.de

*Journal of Translational Medicine* 2019, **17(2):**P25

**Introduction:** Chronic obstructive pulmonary disease (COPD) is a progressive life-threatening lung disease associated with the development of chronic inflammation in the lung in response to inhalation of cigarette smoke (CS). The NO/cGMP pathway with known vasodilatory properties also sets the contractility degree of airway smooth muscle and is further involved in the modulation of immune responses. In this pathway, two NO-sensitive guanylyl cyclases, NO-GC1 and NO-GC2, are engaged in synthetizing cGMP in response to NO.

In lung, both NO-GCs are expressed with the NO-GC1 being responsible for 90% of the NO-GC activity. Consistent with a reduced cGMP formation, pulmonary resistance is higher in NO-GC1 KO mice and increases stronger, when the airways are challenged with Methacholine. This suggests a localisation of NO-GC1 in airway smooth muscle. Regarding the action site of NO-GC2, pulmonary resistance of NO-GC2 KO mice tended to be reduced and was attenuated in response to Methacholine challenge. A finding that assigns NO-GC2 a function different from NO-GC1.

**Methods:** Here, we studied the roles of the NO-GC isoforms in the pathogenesis of COPD by using mice deficient in either NO-GC1 or NO-GC2. To induce COPD we exposed the mice to cigarette smoke in a nose-only exposure system for 5 weeks.

**Results:** Exposure to CS induced the recruitment of inflammatory cells to the bronchoalveolar lavage (BAL). Macrophages numbers increased significantly in NO-GC2 KO and WT mice. In BAL of NO-GC1 KO mice was a trend toward increased alveolar macrophages and therefore the increase upon CS exposure was not significant. In contrast, BAL fluids of CS-exposed NO-GC1 KOs revealed higher lymphocyte levels indicating a role of NO-GC1 in the regulation of immune responses. Measurements of lung function parameters in vivo revealed changes that were more pronounced in CS-exposed NO-GC1 KO mice and tended to be attenuated in NO-GC2 KOs.

**Conclusions:** Thus, we conclude that NO-GC1 deficiency enhances the susceptibility for the development of COPD.

## P26 Peritubular contractile cells in the testis respond to cGMP signaling—new revelations by time-lapse imaging and Fourier analysis

### Andrea Mietens^1^, Sabine Tasch^1^, Gerrit Eichner^2^, Ralf Middendorff^1^

#### ^1^Justus Liebig University, Institute for Anatomy and Cell Biology, Giessen Hesse, Germany; ^2^Justus Liebig University, Mathematical Institute, Giessen Hesse, Germany

##### **Correspondence:** Andrea Mietens - andrea.mietens@anatomie.med.uni-giessen.de

*Journal of Translational Medicine* 2019, **17(2):**P26

**Introduction:** The seminiferous tubules of the testis are surrounded by contractile peritubular cells (PTCs). Their contractile function ensures transportation of spermatozoa which are still immature and immotile when shed from the seminiferous epithelium, towards the epididymal duct where spermatozoa mature and acquire motility as well as fertilizing capacity. PTC contractile function and its regulation contribute to maintaining male fertility. Components of the cGMP signaling system which mediates relaxation of smooth muscle cells were described in contractile PTCs, but functional data are scarce.

**Methods:** To investigate contractility of seminiferous tubules ex vivo, we used a combination of time-lapse imaging and Fourier analysis. Isolated rat and human seminiferous tubules were embedded in a collagen lattice for stabilization and observed by time-lapse imaging. To assess movements of the rat seminiferous tubule wall, changing grey values within defined regions of interest (ROIs) were tracked over time and translated into characteristic frequency spectra by the use of Fourier analysis. Contractile activity of human seminiferous tubules was analyzed by using a kymograph function on the recorded time stacks. cGMP production was enhanced by the NO donor SNP and cGMP degradation was inhibited by the PDE5 inhibitor sildenafil.

**Results:** In the rat seminiferous tubule wall, time-lapse imaging revealed spontaneous contractions with an undulating and irregular contraction pattern. Fourier analysis generated characteristic frequency spectra that form several groups of distinct spectral patterns. Stimulating cGMP generation by SNP slowed down contractile frequency and the corresponding Fourier spectra were shifted towards slower frequencies. This frequency shift was more pronounced when PDE5 was additionally inhibited by sildenafil. First findings point towards an association of certain Fourier spectra with distinct stages of spermatogenesis and a differential susceptibility towards cGMP signaling. In the human testis, disturbances in spermatogenesis are often associated with fibrotic changes of the peritubular structures. Moreover, contractility of the tubules and cGMP effects are also disturbed.

**Conclusions:** Time-lapse imaging combined with Fourier analysis is a valuable tool to study contractile function of seminiferous tubules and its regulation under near-physiological conditions and the impact of various signaling pathways or drugs. Altered cGMP signaling affects function of contractile PTCs in the testis and could contribute to male infertility.

## P27 The effects of the sGC stimulator BAY 41-2272 in an experimental model of Duchenne Muscular Dystrophy (DMD)

### Shalini Murali Krishnan, Ina Hagelschuer, Ilka Mathar, Jutta Meyer, Elke Hartmann, Mira Pavkovic, Peter Sandner

#### Bayer AG, Research & Development, Pharmaceuticals - Heart & Vascular Disease Research, Wuppertal, Germany

##### **Correspondence:** Shalini Murali Krishnan - shalini.murali@bayer.com

*Journal of Translational Medicine* 2019, **17(2):**P27

**Introduction:** Duchenne Muscular Dystrophy (DMD) is a monogenetic disorder affecting 1 in 5000 boys worldwide. The lack of dystrophin causes severe progressive muscle degeneration and weakness. As a result the affected boys require the aid of a wheelchair and non-invasive ventilation already in their teenage years. In addition, it has a significant impact on cardiac function, causing cardiomyopathy and eventually heart failure. Currently, there is no cure for DMD, therefore it is crucial that new therapies for effective medical management are identified to improve the quality of life and prolong life expectancy.

Increasing cGMP levels is known to be cardio-protective and also improve skeletal muscle function. Therefore, in this study, we aimed to determine if an sGC stimulator, BAY41-2272, displayed beneficial effects in the treatment of DMD.

**Methods:** Here, we used 20 wk old, male mdx/mTR^G2^ mice, an experimental model of DMD susceptible to develop cardiomyopathy early. C57Bl6/J mice were used as wild-type controls (WT). Mice received either normal food (NF), or food containing BAY41-2272 (300 ppm) for 16 wks.

**Results:** During treatment, skeletal muscle function was assessed using the rotarod, 4-limb hang and grip strength tests every 4 wks. All 3 techniques demonstrated that mdx/mTR^G2^ mice had reduced skeletal muscle function and treatment with BAY41-2272 improved grip strength. Contrary to a previous report, ECHO assessments demonstrated that mdx/mTR^G2^ mice do not display any cardiac dysfunction at 36 wks, however invasive hemodynamics displayed that mdx/mTR^G2^ mice have reduced mean arterial pressure vs WT and this was further reduced with BAY41-2272 administration.

**Conclusions:** In summary, mdx/mTR^G2^ mice have impaired skeletal muscle function, however they display a normal cardiac phenotype compared to WT mice. BAY41-2272 treatment improves skeletal muscle function and decreased BP compared to mdx/mTR^G2^ mice administered NF. Further investigations are required, to fully assess the levels of muscle damage using histological examination and potential treatment effects of sGC stimulators.

**Acknowledgement:** We would like to thank C. Schade, C. Jochem, E. Clemente, A. Neumann, S. Breetzke, A. Kuhl, C. Elliot, H. Rexing, H. Otte, K. Zinner, K. Schulte and B. Poehler for their technical expertise.

## P28 Development of a cGMP phospho-protein biomarker panel for patient stratification

### Cristian Nogales^1^, Elisa Anastasi^4^, Alexander G. B. Grønning^2^, Alexandra Petraina^1^, Keith Flannagan^4^, Maja Stevanoska^1^, Sarah Chenine^1^, Ana I. Casas^1^, Jan Baumbach^3^, Anil Wipat^4^, Harald H. H. W. Schmidt^1^

#### ^1^Maastricht University, Pharmacology and Personalised Medicine, Maastricht, Netherlands; ^2^University of Southern Denmark, Computational Biology, Odense, Denmark; ^3^Technical University of Munich, Experimental Bioinformatics, Munich, Bavaria, Germany; ^4^University of Newcastle, School of Computing (ICOS), Newcastle, United Kingdom

##### **Correspondence:** Cristian Nogales - c.nogales@maastrichtuniversity.nl

*Journal of Translational Medicine* 2019, **17(2):**P28

**Introduction:** There is a crisis with current disease definitions. Most disease definitions are symptom- or organ-based and have no mechanistic basis. Consequently, different disease mechanisms are clustered under the same disease phenotype and the most marketed drugs have an alarmingly high number needed to treat (NNT). In order to shift to a precision medicine approach, we need to understand the precise mechanisms behind diseases and to develop biomarkers to stratify patients for mechanism-based drug therapy. Pathological cGMP signaling is a common mechanism of a cluster of disease phenotypes such as stroke, heart failure with preserved ejection fraction (HFpEF) and resistant hypertension. The precise defects of impaired cGMP signaling are not well-understood and there are no precision diagnostic tools to stratify patients for cGMP modulating drugs.

**Methods:** In our approach, we make use of the latest developments in recombinant antibodies, constantly evolving databases and new computational tools. Phage Display Technology takes advantage of the structure and biology of bacteriophages, usually the bacteriophage M13, to develop new recombinant antibodies against specific antigens. We use Antibody Phage Display Technology to develop antibodies against new phospho-proteins when no commercial antibodies are available.

**Results:** Here, we generate an antibody panel against proteins modified by cGMP-dependent protein kinase (PKG) for detection of biomarkers in circulating blood and rare circulating cells both in health and disease conditions.

**Conclusions:** We use network data to predict cGMP related biomarkers and new potential therapeutics to tackle pathological cGMP signaling. Stratification of patients in different populations by predictive biomarkers will allow clinicians to target the cause of disease and provide the best possible drug or combination of drugs (Fig. 1).

**Fig. 1:**
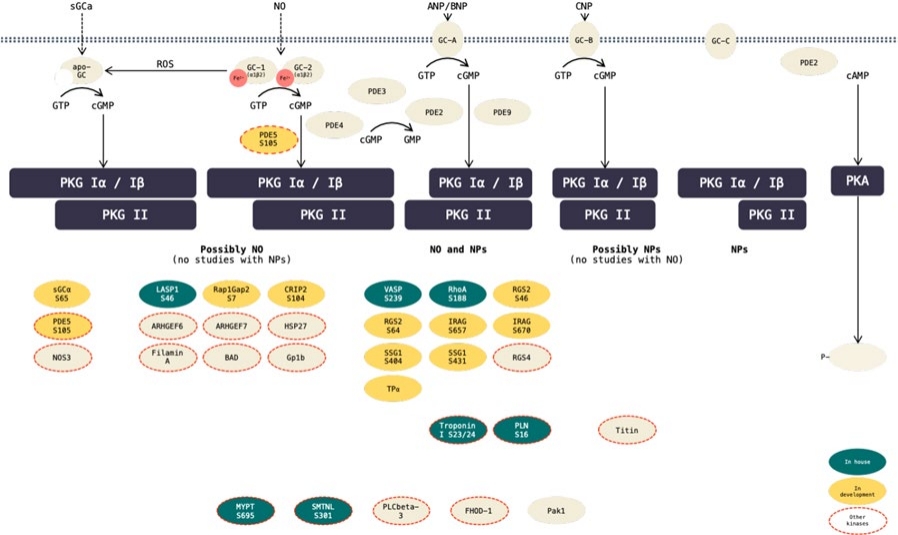
Hypothesis-driven phophoprotein biomarker candidates. Prior unbiased network analysis, potential biomarker candidates were selected through literature screening. Phospho-proteins were clustered in different groups according to evidence suggesting they were phosphorylated by the NO/sGC/cGMP pathway, the NPs/pGC/cGMP pathway, or both

**References**

- Schork NJ. Precision medicine OncoDNA. Nature. 3–5. 10.1038/520609a.
- Langhauser F, et al. A diseasome cluster-based drug repurposing of soluble guanylate cyclase activators from smooth muscle relaxation to direct neuroprotection. NPJ Syst Biol Appl. 2018. 10.1038/s41540-017-0039-7.
- Smith GP. Filamentous fusion phage: novel expression vectors that display cloned antigens on the virion surface. Science (80−). 1985. 10.1126/science.4001944.
- McCafferty J, Griffiths AD, Winter G, Chiswell DJ. Phage antibodies: filamentous phage displaying antibody variable domains. Nature. 1990. 10.1038/348552a0.

## P29 Periprostatic adipose tissue from obese mice reduced the contractile response induced by alpha1-adrenoceptor agonist in mice prostate: is there a role for nitric oxide-cGMP pathway?

### Gabriela R. Passos, Mariana G. de Oliveira, Gabriela M. Bertollotto, Nathália R. Rocha, Edson Antunes, Fabíola Z. Mónica

#### University, University of Campinas-UNICAMP, Campinas, Brazil

##### **Correspondence:** Gabriela R. Passos - gabriela.biomed13@gmail.com

*Journal of Translational Medicine* 2019, **17(2):**P29

**Introduction:** Benign prostatic hyperplasia (BPH) is characterized by an enlarged prostate and greater adrenergic tonus thus causing urinary symptoms. Clinical and experimental data have shown a correlation between BPH and obesity (1, 2). Prostate gland is surrounded by periprostatic adipose tissue (PPAT), which is believed to play a paracrine role and may release pro-inflammatory substances, growth factors, contractile and anti-contractile substances and hormones (3). The aim of this study is evaluate the effect of PPAT on prostate reactivity from lean and obese mice.

**Methods:** Six-week old C57BL/6 male mice were fed with standard chow (lean) or high-fat diet (obese) for 10 weeks. Prostate and PPAT were collect for histological and immunohistochemistry (Ki-67) analysis. PPAT from obese mice was weighted (~ 40 mg), kept in Kreb’s solution (30 min, 37 °C) and the supernatant collected. Prostate segments from lean and obese mice were incubated without and with PPAT supernatant for 30 min. Concentration–response curve to phenylephrine (PE) was carried out. In another set of experiments, the soluble guanylate cyclase inhibitor, ODQ (10 μM), was incubated with PPAT for 30 min and then incubated in prostate segments from both groups. Data represent mean ± SEM. Unpaired t-test or one-way ANOVA were carried out.

**Results:** Histological analysis showed that adipocyte area from PPAT is greater (60%, N = 5, P < 0.05) in obese than in control mice. Histological analysis sections showed a simple columnar epithelium and a thin layer of prostate from lean mice. Obese mice showed prostate epithelial hyperplasia, accompanied by an increase of 93% in Ki67 (25.2 ± 1.6 cells, N = 5, P < 0.05) in relation to control mice (13.0 ± 2.0 cells). Surprisingly, when PPAT supernatant was added in prostate strips from both lean and obese mice a significant reduction by 44% (1.4 ± 0.30, N = 6) and 58% (1.4 ± 0.28, N = 6) (P < 0.05), respectively in PE-induced, contraction was observed in comparison with strips that were not incubated with PPAT (lean: 2.5 ± 0.9 mN vs obese: 3.4 ± 0.53 mN). In the presence of ODQ (PPAT + ODQ) a tendency of increase (2.4 ± 0.4 mN, N = 6, P > 0.05) in PE-induced contraction was observed only in prostate from obese mice when comparing to strips incubated only with PPAT (1.4 ± 0.28 mN, N = 6).

**Conclusions:** In obese mice, an increase on PPAT adipocytes area and prostate hyperplasia were observed. PPAT from obese mice releases anti-contractile substance. More studies are needed in order to assess whether these substances are related to NO-cGMP pathway.

**References**

1. Calmasini FB et al. Obesity-induced mouse benign prostatic hyperplasia (BPH) is improved by treatment with resveratrol: implication of oxidative stress, insulin sensitivity and neuronal growth factor. J Nutr Biochem. 2018;55:53–8.
2. Rohrmann S, Crespo CJ, Weber JR, Smit E, Giovannucci E, Platz EA. Association of cigarette smoking, alcohol consumption and physical activity with lower urinary tract symptoms in older American men: findings from the third National Health And Nutrition Examination Survey. BJU Int. 2005;96(1):77–82.
3. Gutierrez DA, Puglisi MJ, Hasty AH. Impact of increased adipose tissue mass on inflammation, insulin resistance, and dyslipidemia. Curr Diab Rep. 2009;9(1):26–32.

## P30 Abazafil: a first-in-class, highly potent and selective, allosteric inhibitor of phosphodiesterase 5 with penile erection and cognition enhancing properties

### Gary A. Piazza^1^, Sara C. Sigler^1^, Yulia Maxuitenko^1^, Antonio Ward^1^, Maged Alawa^2^, Mohammad Abdel-Halim^2^, Ashraf H. Abadi^2^

#### ^1^University of South Alabama, Mitchell Cancer Institute, Mobile Alabama, US; ^2^German University in Cairo, Department of Pharmaceutical Chemistry, Faculty of Pharmacy and Biotechnology, Cairo, Egypt

##### **Correspondence:** Gary A. Piazza - gpiazza@health.southalabama.edu

*Journal of Translational Medicine* 2019, **17(2):**P30

**Introduction:** PDE5 inhibitors are widely used for the treatment of penile erectile dysfunction, pulmonary arterial hypertension, and benign prostatic hypertrophy, but have undesirable side effects resulting from inhibition of other PDE isozymes.

**Methods:** A novel series of PDE5 inhibitors was discovered from an iterative medicinal chemistry and screening campaign in which a development candidate, abazafil, emerged following extensive chemical optimization.

**Results:** Abazafil inhibited full-length recombinant PDE5 with an IC_50_ value of 1 nM in a non-competitive manner, but did not inhibit cGMP hydrolysis by the PDE5 catalytic fragment at concentrations up to 1000 nM, indicating an allosteric mechanism of enzyme inhibition. In contrast, sildenafil inhibited PDE5 in a competitive manner and exhibited comparable potency to inhibit the full-length and catalytic fragment of PDE5 with IC_50_ values of 3 and 5 nM, respectively. Unlike sildenafil and other PDE5 inhibitors that non-selectively inhibit other PDE isozymes (e.g. PDE6 and PDE11), abazafil did not significantly inhibit other PDE isozyme at concentrations up to 15,000 fold higher than its IC_50_ value to inhibit PDE5. In cell-based experiments, abazafil increased intracellular cGMP levels, as determined by cGMP biosensor and immunoassays, and activated PKG, as measured by phospho-VASP levels in a concentration- and time-dependent manner. PK studies revealed that abazafil can generate plasma levels exceeding its PDE5 IC_50_ value by > 1000-fold with a T_max_ of ~ 2 h. Dosages up to 150 mg/kg twice daily were well tolerated in mice for up to 28 days. Abazafil (70 mg/kg) significantly increased the length of the penis from 2.20 mm in control group to 3.75 mm in the treated group within 1 h of treatment. Abazafil also reduced the deleterious effects of LPS-induced neuro-inflammation in mice by improving memory and spatial recognition.

**Conclusions:** Abazafil non-competitively inhibits PDE5 by an allosteric mechanism with high potency and PDE isozyme selectivity. The unique chemical structure, mechanism of binding, and superior potency and PDE isozyme selectivity of abazafil compared with known PDE5 inhibitors, could provide efficacy advantages and reduced side-effects for erectile dysfunction treatment and other indications. Abazafil’s ability to reduce neuro-inflammation, improve cognitive function, and increase penile length, along with tolerability in mice supports further preclinical development. Abazafil represents a novel PDE5 inhibitor with an allosteric mechanism of action and potential for improved efficacy and reduced side effects (Fig. 1).

**Fig. 1:**
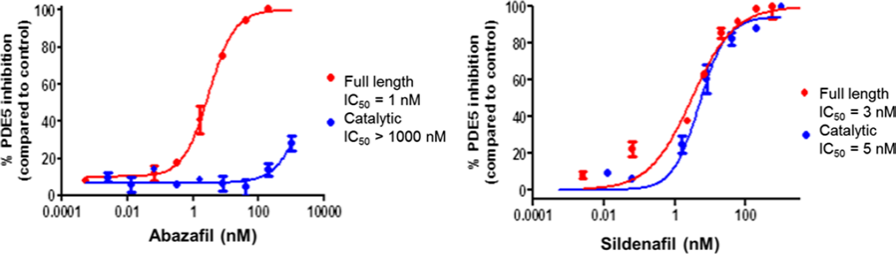
Allosteric inhibition of PDE5 by abazafil. Abazafil potently inhibits the full length PDE5, but not the catalytic fragment, while sildenafil inhibits the full length PDE5 and the catalytic fragment

**Acknowledgement:** We are grateful to Dr. Hengming Ke (University of North Carolina Chapel Hill, NC) for his generous gift of plasmids for the catalytic domain of PDE5.

## P31 Activation of guanylyl cyclase-B increases long bone mass, density and strength

### Jerid Robinson^1^, Nicholas Blixt^2^, Gordon Warren^3^, Zhou Ye^4^, Andrew Benton^3^, Conrado Aparicio^4^, Larry Suva^5^, Kim Mansky^2^, Dana Gaddy^5^, Lincoln Potter^1^

#### ^1^University of Minnesota, Department of Biochemistry, Minneapolis Minnesota, US; ^2^University of Minnesota, Department of Cell Biology, Minneapolis Minnesota, US; ^3^Georgia State University, Department of Physical Therapy, Atlanta Georgia, US; ^4^University of Minnesota, Department of Restorative Sciences, Minneapolis Minnesota, US; ^5^Texas A&M University, Department of Veterinary Physiology and Pharmacology, College Station Texas, US

##### **Correspondence:** Lincoln Potter - potter@umn.edu

*Journal of Translational Medicine* 2019, **17(2):**P31

**Introduction:** C-type natriuretic peptide (CNP) activation of guanylyl cyclase (GC)-B, also known as npr2 or NPR-B, increases intracellular cGMP and stimulates long bone growth. CNP activation requires the phosphorylation of multiple GC-B residues and dephosphorylation inactivates the receptor. Knock-in GC-B^7E/7E^mice expressing a phosphomimetic, glutamate-substituted, form of GC-B that cannot be inactivated by dephosphorylation, have increased CNP-dependent GC activity and longer bones (1). Here, we investigate whether male nine-week old GC-B^7E/7E^mice have increased bone mass and strength.

**Methods:** GC-B^7E/7E^mice (originally described as *Npr2*^*7E/7E*^) maintained on a C57BL/6 background as previously described (2). Right tibia from 9-week-old male GC-B^WT/WT^and GC-B^7E/7E^mice were scanned using the XTH 225 micro-computed tomography (mCT) machine (Nikon Metrology Inc., Brighton, MI, USA) at an isotropic voxel size of 6.7 µm. For tibial experiments, the same bone used for mCT were also analyzed by 3-point bending as previously described (3). Procollagen 1N-terminal propeptide and collagen 1 C-terminal telopeptide were measured from serum obtained WT and GC-B^7E/7E^mice at the ages indicated in the respective figures using mouse P1NP and RatLaps (CTX-1) enzyme immunoassays from Immunodiagnostic Systems (Boldon, UK) according to the manufacturer’s instructions.

**Results:** Male 9-week old GC-B^7E/7E^mice have 43% greater trabecular bone volume, 40% greater trabecular number and 17% less trabecular separation than wild type tibiae. GC-B^7E/7E^tibiae also have 10% greater cortical cross-sectional area, 9% greater cortical thickness, 6% greater periosteal diameter and a 24% greater cortical cross-sectional moment of inertia. L5 vertebra bone volume fraction was also significantly greater in the male mutant mice. Trabecular, but not cortical, bone mineral density was elevated 40% in tibiae from the mutant mice. Three-point bending analysis determined that mutant tibiae and femurs had 35% and 36% greater ultimate load and 35% and 49% greater stiffness, respectively. No difference in microhardness indicated similar bone quality. Serum P1NP and osteocalcin, but not CTX, levels were elevated in the GC-B^7E/7E^mice. Once daily injections of the CNP analog, BMN-111, increased trabecular bone volume fraction and number in adult male and female tibiae. BMN-111 increased osteocalcin in serum from males and females but only increased CTX levels in females.

**Conclusions:** We conclude that GC-B activation enhances bone turnover, which increases bone volume, density and strength and suggest that the GC-B pathway may be a target for novel drugs that treat osteoporosis and/or promote fracture healing.

**References**

- Shuhaibar LC, Robinson JW, Vigone G, Shuhaibar NP, Egbert JR, Baena V, et al. Dephosphorylation of the NPR2 guanylyl cyclase contributes to inhibition of bone growth by fibroblast growth factor. Elife. 2017;6.
- Shuhaibar LC, Egbert JR, Edmund AB, Uliasz TF, Dickey DM, Yee SP, et al. Dephosphorylation of juxtamembrane serines and threonines of the NPR2 guanylyl cyclase is required for rapid resumption of oocyte meiosis in response to luteinizing hormone. Dev Biol. 2016;409(1):194–201.

## P32 Increased C-type natriuretic peptide-dependent guanylyl cyclase-B activity rescues achondroplastic dwarfism

### Jerod Robinson^1^, Yuan-Tsong Chen^2^, Yi-Ching Lee^2^, Lincoln Potter^1^

#### ^1^University of Minnesota, Department of Biochemistry, Minneapolis Minnesota, US; ^2^Institute of Biomedical Sciences, Academia Sinica, Taipei, Taiwan

##### **Correspondence:** Lincoln Potter - potter@umn.edu

*Journal of Translational Medicine* 2019, **17(2):**P32

**Introduction:** Activating mutations in a single allele of the fibroblast growth factor receptor 3 (FGFR3) or inactivating mutations in both alleles of guanylyl cyclase B (GC-B), also known as NPR-B or Npr2, cause short-limbed dwarfism. FGFR3 activation causes dephosphorylation and inactivation of GC-B, suggesting that GC-B is downstream of FGFR3 in the dwarfism pathway (1, 2). Mice expressing GC-B with glutamate-substitutions for known phosphorylation sites (GC-B-7E) have increased C-type natriuretic peptide-dependent guanylyl cyclase activity and skeletal overgrowth (1). We bred mice expressing none, one or two alleles of the human FGFR3-G380R gain-of-function mutation (3) with mice expressing none, one or two alleles of GC-B-7E (4) to determine if GC-B dephosphorylation is required for achondroplasia.

**Methods:** Male and female wild type and GC-B^7E/7E^mice were genotyped and maintained on a mixed C57BL6/129 Sv background as previously described (24). FGFR3^ACH/ACH^ mice were genotyped and maintained on a mixed C57BL6/129Sv line (25). Naso-anal measurements were carried out on conscious male and female mice once weekly from weeks 4 to 16 weeks for the duration of the study using a digital caliper.

**Results:** We bred mice expressing none, one or two alleles of the human FGFR3-G380R gain-of-function mutation (3) with mice expressing none, one or two alleles of GC-B-7E (4) to determine if GC-B dephosphorylation is required for achondroplasia. Expression of one FGFR3-G380R allele decreased length in male but not female mice. Expression of two FGFR3-G380R alleles decreased length in both sexes but the effect was greater in males. Expression of one GC-B-7E allele increased length in females but not males. Expression of two GC-B-7E alleles increased skeletal length in both sexes but the effect was greater in females. Expression of two FGFR3-G380R alleles reduced skeletal length to the same degree in mice expressing either wild type GC-B or GC-B-7E, which is consistent with GC-B dephosphorylation not being required for achondroplasia. However, expression of two GC-B-7E alleles rescued the decreased skeletal length associated with expression of two FGFR3-G380R alleles in both sexes.

**Conclusions:** For the first time, these results describe sex-specific differences in the GC-B and FGFR3 long bone growth pathways. Furthermore, they indicate that GC-B activation increases the growth of both wild type and achondroplastic mice through a pathway that is antagonistic and independent of the FGFR3 pathway (Fig. 1).

**Fig. 1:**
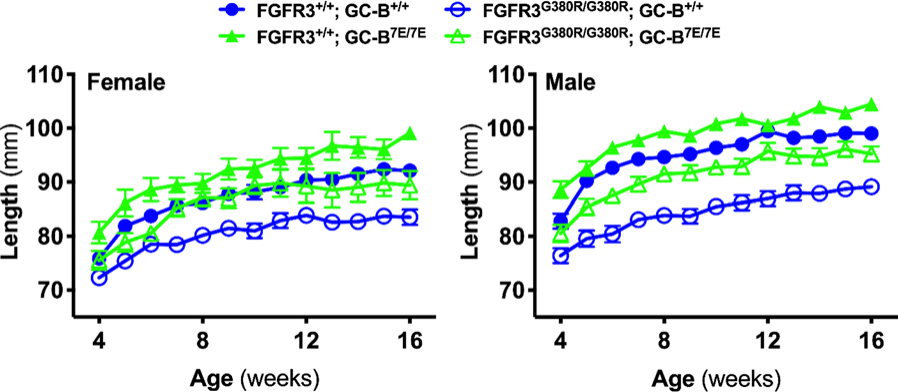
Naso-anal lengths of FGFR3^+/+^ or FGFR3ACH/ACH mice expressing GC-B^+/+^ or GC-B^7E/7E^. Naso-anal lengths of mice were measured with a digital caliper once weekly from 4 to 16 weeks of age. Lengths (mm) of FGFR3^+/+^; GC-B^+/+^, FGFR3^+/+^; GC-B^7E/7E^, FGFR3^ACH/ACH^; GC-B^+/+^, and FGFR3^ACH/ACH^; GC-B^7E/7E^ mice

**Acknowledgement:** This work was supported by National Institutes of Health Grant R01GM098309, a University of Minnesota Foundation Bridge Grant to LRP, a University of Minnesota-Mayo Clinic Partnership grant, a University of Minnesota Academic Health Center Faculty Research and Development Grant to LRP as well as an NIHT32DK007203 Grant to JWR. Grants from the Fund for Science and the Hormone Receptor Fund to LRP.

**References**

- Shuhaibar LC, Robinson JW, Vigone G, Shuhaibar NP, Egbert JR, Baena V, et al. Dephosphorylation of the NPR2 guanylyl cyclase contributes to inhibition of bone growth by fibroblast growth factor. Elife. 2017;6.
- Robinson JW, Egbert JR, Davydova J, Schmidt H, Jaffe LA, Potter LR. Dephosphorylation is the mechanism of fibroblast growth factor inhibition of guanylyl cyclase-B. Cell Signal. 2017;40:222–9.
- Lee YC, Song IW, Pai YJ, Chen SD, Chen YT. Knock-in human FGFR3 achondroplasia mutation as a mouse model for human skeletal dysplasia. Sci Rep. 2017;7:43220.
- Shuhaibar LC, Egbert JR, Edmund AB, Uliasz TF, Dickey DM, Yee SP, et al. Dephosphorylation of juxtamembrane serines and threonines of the NPR2 guanylyl cyclase is required for rapid resumption of oocyte meiosis in response to luteinizing hormone. Dev Biol. 2016;409(1):194–201.

## P33 Soluble guanylate cyclase stimulator vericiguat enhances memory processes through GluA1-AMPA receptor trafficking

### Ellis Nelissen^1^, Elentina Argyrousi^1^, Nick P. van Goethem^1^, Peter Sandner^2^, Jos Prickaerts^1^

#### ^1^Maastricht University, Department of Psychiatry and Neuropsychology, School for Mental Health and Neuroscience, Maastricht, Netherlands; ^2^Bayer AG, Drug Discovery, Wuppertal, Germany

##### **Correspondence:** Jos Prickaerts - jos.prickaerts@maastrichtuniversity.nl

*Journal of Translational Medicine* 2019, **17(2):**P33

**Introduction:** Cognitive impairment is one of the main symptoms of Alzheimer’s disease, which negatively impacts the quality of life of patients. Therefore, a pharmacological intervention that has memory enhancing effects would be beneficial to patient outcomes. Previous studies have implicated the importance of the intracellular cGMP-PKG signaling pathway in memory processes. This pathway is initiated through the activation of soluble guanylate cyclase (sGC) by nitric oxide (NO). sGC stimulators enhance sGC activity by directly stimulating its production while also increasing sGC sensitivity to endogenous NO. In this experiment we hypothesized that sGC stimulator vericiguat could have beneficial effects on memory functioning through enhanced cGMP-PKG signaling and subsequent increased GluA1-AMPA receptor (AMPAR) trafficking.

**Methods:** To evaluate the effects on long-term memory functioning in rats, different oral dosages of vericiguat or vehicle were administered 30 min before T1 of the object location task (OLT) to investigate memory acquisition processes. A 24 h inter-trial interval was used. To evaluate the effects on GluA1-AMPAR trafficking, an acute mouse hippocampal slice model was used to chemically induce late time potentiation (chemLTP). The slices would either be incubated with vericiguat immediately before chemLTP induction to investigate acquisition-like processes, or 10 min after chemLTP induction to investigate early consolidation-like processes. GluA1 subunit dynamics were measured using a special western blot protocol.

**Results:** It was found that 0.3 and 1 mg/kg vericiguat were able to significantly improve long term memory performance in the OLT. Additionally, treatment with 10 nM vericiguat increased chemLTP-induced trafficking to the membrane of a pre-existing pool of GluA1-AMPARs in acquisition-like processes only, which was found to be independent to phosphorylation of the receptor on S845.

**Conclusions:** These data suggests that vericiguat enhances memory function in rats and that the in vivo memory improvement is acquisition driven.

**Acknowledgement:** Funding was provided by Bayer AG and Merck Sharp & Dohme Corp., a subsidiary of Merck & Co., Inc., Kenilworth, NJ, USA.

## P34 New potential soluble guanylyl cyclase (sGC) activators: Design, synthesis and NMR-driven interaction studies with the *Nostoc* sp. HNOX domain

### Aggeliki Roumana, Aikaterini I. Argyriou, Garyfallia I. Makrynitsa, Styliani A. Chasapi, Minos-Timotheos Matsoukas, Stavros Topouzis

#### University of Patras, Dept. of Pharmacy, Rio-Patras, Greece

##### **Correspondence:** Aggeliki Roumana - aggelikiroumana@gmail.com

*Journal of Translational Medicine* 2019, **17(2):**P34

**Introduction:** Soluble guanylyl cyclase (sGC) plays a crucial role in mediating the biological effects of nitric oxide (NO). It is a heterodimer consisting of one alpha (*α*_1_ or *α*_2_) and one beta (*β*_1_ or *β*_2_) subunit, with a prosthetic heme group located in the *β1* H-NOX regulatory domain. sGC catalyzes the conversion of guanosine triphosphate (GTP) to the second messenger cyclic guanosine monophosphate (cGMP), upon binding of nitric oxide (NO) to the heme group [1]. A number of pathological states, in particular cardiovascular and pulmonary diseases [2] have been at least partly attributed to impairment of the NO-sGC-cGMP pathway, due for example to low NO bioavailability and/or heme oxidation and subsequent sGC dysfunction. Besides NO donors, two functional classes of sGC agonists have been identified: the heme-dependent sGC stimulators such as the recently approved drug riociguat (BAY 63-2521) and the clinically evaluated vericiguat (BAY 1021189) [2] as well as the heme-independent sGC activators, such as the amino dicarboxylic acid derivative cinagiguat (BAY 58-2667) [2]. Notably, the enzymatic activity of sGC by sGC activators is enhanced after oxidation or removal of the heme moiety due for example to oxidation of the heme iron and loss of the heme group from sGC, a change elicited by synthetic tricyclic oxadiazolone derivatives such as the closely related analogues ODQ and NS2028 [3, 4].

**Methods:** In the present study, following a rational drug design approach, we designed new potential sGC activators with structural features which may enable both the oxidation of the heme moiety (e.g. a fused oxadiazolidinone ring) and sGC activation (e.g. appropriate carboxylic substituents).

**Results:** Our presentation will describe (a) the progress of our efforts towards the synthesis of the target molecules and (b) the characterization by NMR-based analysis of the binding mode of a sGC activator, cinaciguat (BAY 58-2667), with recombinant *Nostoc* sp. HNOX domain.

**Conclusions:** Our study aims to help elucidate the requisite features of rationally-designed compounds as well as the precise molecular events critical for NO/heme-independent activation of the sGC.

**Acknowledgement:** Τhe research/project “Novel functional activators of the soluble Guanylate Cyclase (sGC)” *ΕΔΒΜ*-*34,* # 80436 is implemented through/has been co-financed by the Operational Program “Human Resources Development, Education and Lifelong Learning” and is co-financed by the European Union (European Social Fund) and Greek national funds.

**References**

- Childers KC, Garcin ED. Nitric oxide. 2018;77:53–64.
- Sandner P, et al. Handbook of experimental pharmacology. Berlin: Springer. 2018.
- Zhao Y, et al. Biochemistry. 2000;39:10848–54.
- Olesen SP, et al. Br J Pharmacol. 1998;123:299–309.

## P35 Tyrosine 135 of the β_1_ subunit as binding site of BR 11257: at arm’s length from thermostabilisation

### Christin Elgert^1^, Peter Sandner^2^, Sönke Behrends^1^, Anne Rühle^1^

#### ^1^TU Braunschweig, Institut für Pharmakologie, Toxikologie und Klinische Pharmazie, Braunschweig Lower Saxony, Germany; ^2^Bayer AG, Cardiovascular Research, Wuppertal North Rhine-Westphalia, Germany

##### **Correspondence:** Anne Rühle - a.soemmer@tu-bs.de

*Journal of Translational Medicine* 2019, **17(2):**P35

**Introduction:** In our recent publication Elgert et al. 2019, we compared two sGC activators, the dicarboxylic acid BAY 60-2770 and the monocarboxylic acid BR 11257, in activity and thermostability measurements [1]. Despite consistent results in activity measurements with similar EC_50_ values, we observed unexpected differences in thermostabilisation of sGC: While BAY 60-2770 had a significant thermostabilising influence on sGC, this effect was absent for BR 11257.

Important binding sites for the activators’ carboxylic acids are the hydrogen bond donating amino acids in the conserved Y-x-S-x-R motif [2]. To compare the importance of this hydrogen bond network for thermostabilisation and activation by the dicarboxylic acid BAY 60-2770 and the monocarboxylic acid BR 11257, we performed measurements with the point mutated sGC variants α_1_β_1_Y135F and α_1_β_1_R139L.

**Methods:** Purification of sGC from Sf9 cells. UV VIS absorbance analyses. Activity measurements with [α-32P]-GTP. Thermostability measurements in protein thermal shift assay. All methods are described in detail in Elgert et al. 2019.

**Results:** Mutants that lack either Y135 or R139 for hydrogen bond formation, are still activated by BAY 60-2770 with unaffected affinity. In contrast, thermostabilisation is not (α_1_β_1_R139L) or only poorly detectable (α_1_β_1_Y135F). Accordingly, the dicarboxylic acid BAY 60-2770 seems to act like a monocarboxylic acid when only one amino acid is available for hydrogen bond formation.

Activation by BR 11257 is unaffected in α_1_β_1_R139L, while affinity is decreased in α_1_β_1_Y135F.

**Conclusions:** Y135 and R139 are crucially involved in thermostabilisation. Binding to both amino acids is necessary, explaining the lack of thermostabilisation with the monocarboxylic acid BR 11257. In contrast, one hydrogen bond either to Y135 or R139 is sufficient for activation by activator drugs. This is in line with the similar activation profiles of BR 11257 and BAY 60-2770. However, the mutant α_1_β_1_Y135F allows for a discrimination between mono- and dicarboxylic acids in activity measurements: a tenfold higher EC_50_ value indicates a lower affinity towards BR 11257. The only carboxylic acid of BR 11257 seems to interact with Y135 and not with R139. Accordingly, mutation of the main interaction partner Y135 lowers the affinity towards BR 11257, while mutation of R139 has no influence on activation by BR 11257 (Fig. 1).

**Fig. 1:**
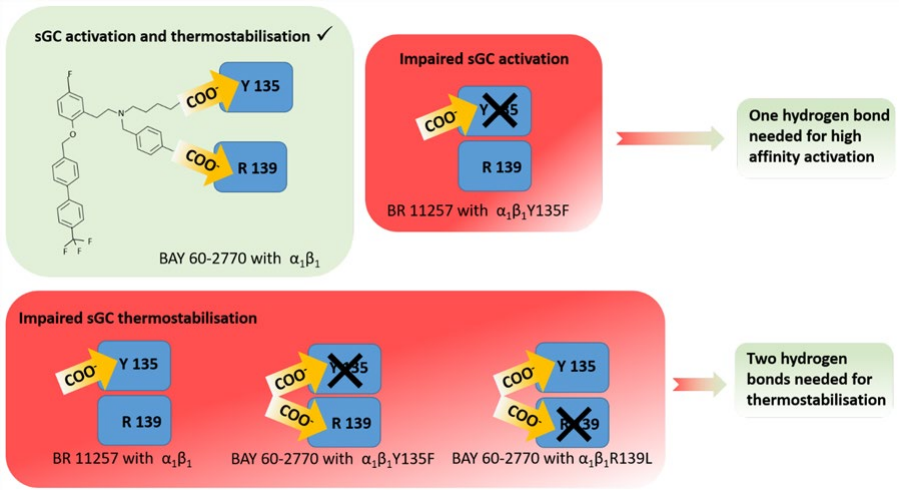
Importance of amino acids Y135 and R139 for sGC activation and thermostabilisation

**Acknowledgement:** The expert technical assistance of Ines Glöckner, Ines Thomsen and Gerlind Henze-Wittenberg is gratefully acknowledged.

**References**

1. Elgert C, Rühle A, Sandner P, Behrends S. A novel soluble guanylyl cyclase activator, BR 11257, acts as a non-stabilising partial agonist of sGC. Biochem Pharmacol. 2019;163:142–53. 10.1016/j.bcp.2019.02.007.
2. Kumar V, Martin F, Hahn MG, Schaefer M, Stamler JS, Stasch J-P, van den Akker F. Insights into BAY 60-2770 activation and *S*-nitrosylation-dependent desensitization of soluble guanylyl cyclase via crystal structures of homologous nostoc H-NOX domain complexes. Biochemistry. 2013;52(20):3601–8. 10.1021/bi301657w.

## P36 Activation of PKG/PKA inhibit procoagulant platelet formation but not caspase-dependent platelet apoptosis

### Natalia Rukoyatkina^1^, Valentina Shpakova^1^, Ulrich Walter^2^, Kerstin Jurk^2^, Stepan Gambaryan^1,2^

#### ^1^Sechenov Institute of Evolutionary Physiology and Biochemistry, Russian Academy of Sciences, St. Petersburg, Russia; ^2^Center for Thrombosis and Hemostasis (CTH), Johannes Gutenberg University, Mainz, Germany

##### **Correspondence:** Natalia Rukoyatkina - natalia.rukoyatkina@gmail.com

*Journal of Translational Medicine* 2019, **17(2):**P36

**Introduction:** Negatively charged phosphatidylserine (PS) exposure on platelet surface is a common hallmark of procoagulant and apoptotic platelets. PKG and PKA activity mediates inhibition of almost any agonist-induced platelet activating pathways including calcium signalling, integrin activation, degranulation, shape change, adhesion, aggregation, etc. However, the question whether activation of PKG/PKA will inhibit platelet apoptosis or formation of procoagulant platelets still not fully defined.

**Methods:** Human, wild type, and platelet specific sGC KO mouse platelets were used to evaluate effects of PKG/PKA activation on procoagulant and apoptotic platelets. Procoagulant platelet formation was induced by combination of strong platelet activators (thrombin/convulxin, Thr/Cvx), platelet apoptosis was induced by administration of anticancer drug ABT-737 or selective BCL-XL inhibitor WEHI-539.

**Results:** Stimulation of platelets by Thr/Cvx (0.01 U/ml/5 ng/ml) leads to intracellular calcium mobilization, integrin αIIbβ3 activation and strong (five- to sixfold, compared to the control) increase of PS surface exposure without caspase 3 activation. PKG activation by NO donors and PKA activation by forskolin strongly inhibited all these events including PS exposure (procoagulant platelet formation). In human platelets preincubated with sGC inhibitor (ODQ, 5 µM) and in sGC KO mouse platelets inhibitory effects of PKG was blunted, indicating that inhibitory effects of PKG is mediated by sGC/cGMP/PKG pathway.

Incubation of platelets with ABT-737 or WEHI-539 did not increase intracellular calcium concentration but induced caspase-3-dependent apoptosis with a strong increase of PS surface exposure. In contrast to procoagulant platelets, PKG activation by NO donors and PKA activation by forskolin did not inhibit PS surface exposure mediated by caspase 3 activation. Additionally, caspase 3 activation results in strong VASP phosphorylation which directly correlates with significantly reduced agonists-induced platelet activation.

**Conclusions:** In summary, we show here that PKG/PKA activation strongly inhibited procoagulant platelet formation whereas caspase-dependent platelet apoptosis is not inhibited by activity of these kinases.

**Acknowledgement:** The work was supported by the grant from RFBR № 17-00-00141 (17-00-00139).

## P37 Hyperosmotic stress induces cGMP release in podocytes

### Nelli Rutkowski^1^, Julia Binz^1^, Thomas Benzing^1,2^, Matthias Hackl^1^

#### ^1^Department II of Internal Medicine and Center for Molecular Medicine Cologne, University of Cologne, Faculty of Medicine and University Hospital Cologne, Cologne North Rhine-Westphalia, Germany; ^2^CECAD, University of Cologne, Faculty of Medicine and University Hospital Cologne, Cologne North Rhine-Westphalia, Germany

##### **Correspondence:** Nelli Rutkowski - nell4ik90@web.de

*Journal of Translational Medicine* 2019, **17(2):**P37

**Introduction:** Chronic kidney disease is associated with a microvascular dysfunction of the glomerular filtration apparatus, increased glomerular hydrostatic pressure and hyperfiltration. Podocytes wrap around the capillaries of the glomerulus maintaining the filtration barrier through their interdigitated foot processes and are exposed to the hydrostatic pressure. To adapt to the environmental forces the foot processes form a dynamic cytoskeleton consisting of an actin-based contractile apparatus regulated by mechanical stress and vasoactive hormones. Podocyte foot process effacement is considered to be a culprit of glomerular disease and is a pathologic event in hyperosmolar hyperglycemic state. Podocytes express receptors for natriuretic peptides and nitric oxide and thereby synthetize the cyclic guanosine 3′,5′-monophosphate (cGMP). cGMP is instrumental in regulating the function of glomeruli by influencing vascular cell types and regulating vasomotor tone. However, the knowledge about a cGMP pathway in podocyte mechanobiology is limited to a few in vitro studies. In order to gain a better understanding of spatial cGMP dynamics, we aimed at showing if and to what extent podocytes cGMP levels respond to osmolality changes induced by naturally occurring osmolytes.

**Methods:** We isolated glomeruli from mice expressing a cytosolic cGMP sensor (R26-cGi500) ubiquitously in all cells of the glomerulus. cGi500 is a FRET-based ratiometric cGMP biosensor which undergoes a conformational change upon cGMP binding leading to a measurable decrease in FRET efficiency. The isolated glomeruli were stimulated for 3 min with different substances (CNP, DEA/NONOate, SNAP). Confocal microscopy with a superfusion system was used to visualize the changes in cGMP levels. In addition, we tested different osmolytes in various concentrations (d-Glucose, Urea) and osmotic control agents (d-Mannitol + d-Glucose, 3-Methyl-*O*-Glucose).

**Results:** A reproducible elevation of cGMP levels was observed after the stimulation of the NO/cGMP and CNP/cGMP signaling pathway in R26-cGi500 mice. d-Glucose concentration (30 mM) which are postulated to initiate cGMP release in vitro did not stimulate a signal in isolated glomeruli of cGi500 mice. Only higher concentrations of Glucose (250 mM; 500 mM), 500 mM d-Mannitol and Urea (200 mM; 600 mM) caused an increased cGMP release compared to baseline. Furthermore, a marked shrinkage of the whole glomerulus was evident.

**Conclusions:** Taken together, our findings suggest that hyperosmotic solutions can induce cGMP release in cells of the isolated glomeruli. Furthermore glucose and hyperosmotic insult may act in an additive manner.

## P38 Elucidating the role of the effector protein ExoY in *Pseudomonas aeruginosa* infections

### Bastian Schirmer^1^, Christina Kloth^1,2^, Christian Kaiser^1^, Antje Munder^3^, Solveig Kälble^1^, Roland Seifert^1^

#### ^1^Hannover Medical School, Institute of Pharmacology, Hannover Lower Saxony, Germany; ^2^Hannover Medical School, Institute of Experimental Hematology, Hannover Lower Saxony, Germany; ^3^Hannover Medical School, Clinic for Paediatric Pneumology and Neonatology, Hannover Lower Saxony, Germany

##### **Correspondence:** Bastian Schirmer - schirmer.bastian@mh-hannover.de

*Journal of Translational Medicine* 2019, **17(2):**P38

**Introduction:** Exotoxin Y (ExoY) of *Pseudomonas aeruginosa* is delivered via the bacterial type III secretion system into host cells, where nucleotidyl cyclase activity of ExoY is activated by F-actin [1]. In addition to cyclic GMP (cGMP) and cyclic AMP (cAMP), ExoY synthesizes cyclic 3′,5′-uridylylmonophosphate (cUMP) and the corresponding cytidylyl analogue cCMP [2]. We hypothesize that cGMP and cUMP exert distinct functions as first or second messengers during the bacterial infection.

**Methods:** In order to elucidate the function of cGMP and cUMP during the course of the infection, we investigated the concentrations and effects of ExoY-generated cNMP in an acute mouse lung infection model [3], and studied a potential suppressive function of the cNMPs in murine and human immune cells. Furthermore, we have begun to explore a second messenger role of cUMP in T cells. Based on the recently published structure [4] and complemented by molecular dynamic simulations, we also generated ExoY mutants in order to analyze the structure dependence of the substrate spectrum.

**Results:** In the lungs of mice infected with ExoY-expressing *P. aeruginosa*, high concentrations of cUMP were found. This increase in cUMP correlated with an altered immune response as compared to control mice infected with a cyclase-deficient bacterial mutant. Surprisingly, cGMP concentrations were not elevated by the toxin in infected lungs. A first hint for a specific function of cUMP in the infected host itself was found in αCD3/αCD28-stimulated human peripheral blood mononuclear cells (PBMC): cGMP and the cyclic pyrimidine nucleotides cCMP and cUMP inhibited IL-2 production in these cells (see Fig. 1). These effects could be, in part, confirmed in naïve mouse CD4 + T-cells, too. In order to further elucidate the roles of individual cNMPs during *P. aeruginosa* infection, several mutants were generated based on structural analyses and are currently being tested for altered substrate specifities.

**Fig. 1:**
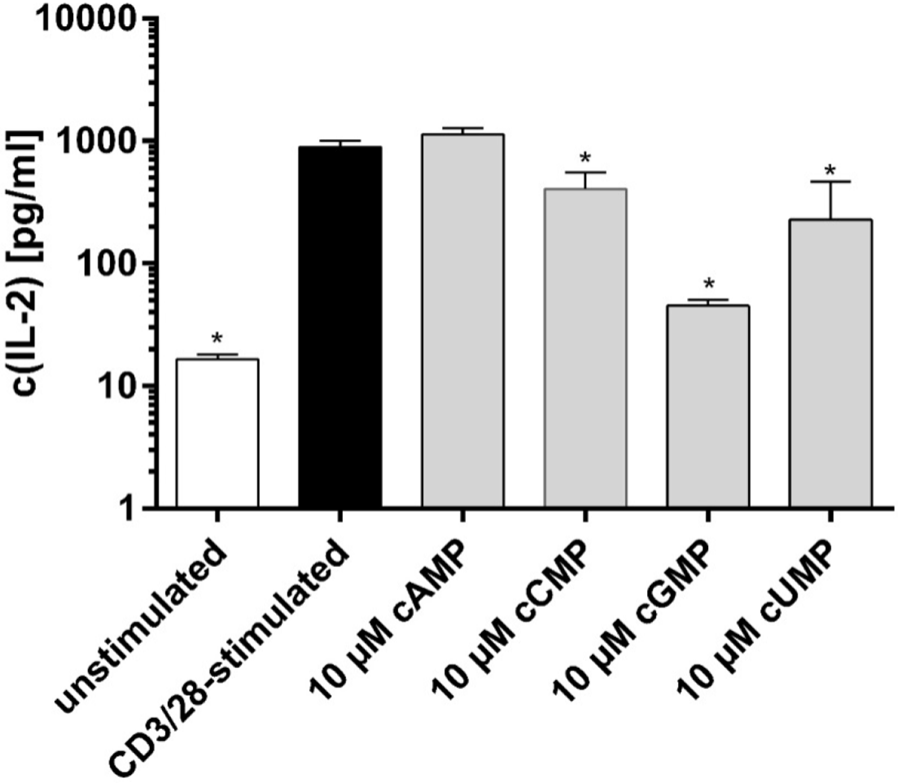
3′,5′-cyclic GMP/CMP/UMP inhibit IL-2 production in PBMC. IL-2 concentrations were measured with ELISA in supernatants of human peripheral blood mononuclear cells (PBMC) that had been stimulated with ImmunoCult™ human CD3/CD28 T cell activator in presence or absence of 10 µM cNMP and that had been incubated for 48 h at 37 °C/5% (v/v) CO_2_. Unstimulated PBMC served as control. Shown are the means ± SD of n = 3 individual experiments. Differences with p < 0.05 (one-way ANOVA with Dunnett’s post hoc correction) were considered statistically significant (*)

**Conclusions:** The observed effects of cNMPs in our lung infection model may be due to an immune-modulatory role of cUMP as a first or second messenger. This modulation may be exerted by T cells, especially when focusing on chronic diseases, because both cUMP and cGMP influenced the IL-2 production in PBMC and purified T cells. Further research is needed to uncover the underlying molecular mechanisms, but T cells have proven to be a promising model system for further investigations of the immunologic effects of cUMP and cGMP. ExoY mutants with altered substrate specificity will be a valuable tool in these investigations and might contribute to the elucidation of the different behaviour of ExoY in vivo (cUMP > cGMP) and in vitro (cGMP > cUMP).

**References**

1. Belyy A, Raoux-Barbot D, Saveanu C, Namane A, Ogryzko V, Worpenberg L, et al. Actin activates *Pseudomonas aeruginosa* ExoY nucleotidyl cyclase toxin and ExoY-like effector domains from MARTX toxins. Nat Commun. 2016;7:13582.
2. Beckert U, Wolter S, Hartwig C, Bähre H, Kaever V, Ladant D, et al. ExoY from *Pseudomonas aeruginosa* is a nucleotidyl cyclase with preference for cGMP and cUMP formation. Biochem Biophys Res Commun. 2014;450(1):870–4.
3. Kloth C, Schirmer B, Munder A, Stelzer T, Rothschuh J, Seifert R. The role of *Pseudomonas aeruginosa* ExoY in an acute mouse lung infection model. Toxins. 2018;10(5), pii:E185.
4. Khanppnavar B, Datta S. Crystal structure and substrate specificity of ExoY, a unique T3SS mediated secreted nucleotidyl cyclase toxin from *Pseudomonas aeruginosa*. Biochim Biophys Acta. 2018;1862(9):2090–103.

## P39 The absence of cGMP-mediated sensory axon bifurcation affects nociception and termination fields of afferents in the spinal cord

### Philip Tröster^1^, Julia Haseleu^1^, Jonas Petersen^2,3^, Oliver Drees^3^, Achim Schmidtko^2,3^, Frederick Schwaller^1^, Gary R. Lewin^1^, Gohar Ter-Avetisyan^1^, York Winter^4^, Stefanie Peters^5^, Susanne Feil^5^, Robert Feil^5^, Fritz G. Rathjen^1^, Hannes Schmidt^1,5^

#### ^1^Max Delbrück Center for Molecular Medicine in the Helmholtz Association, Berlin, Germany; ^2^Goethe University, Institute of Pharmacology, Frankfurt A.M., Germany; ^3^Witten/Herdecke University, Institute of Pharmacology and Toxicology, Witten, Germany; ^4^Charité, Cognitive Neurobiology, Berlin, Germany; ^5^University of Tübingen, Interfaculty Institute of Biochemistry, Tübingen, Germany

##### **Correspondence:** Hannes Schmidt - hannes.schmidt@uni-tuebingen.de

*Journal of Translational Medicine* 2019, **17(2):**P39

**Introduction:** Axonal branching is a key principle for the establishment of neuronal circuitry, exemplified, e.g., by the intricate innervation patterns of dorsal root ganglion (DRG) neuron projections into the spinal cord which provides the basis for primary sensory representation of the body within the central nervous system. Previously, our search for molecular determinants of axonal branching identified a cGMP signaling cascade composed of the ligand C-type natriuretic peptide (CNP; gene name *Nppc*), the receptor guanylyl cyclase B (GC-B; gene name *Npr2*), and cGMP-dependent protein kinase I (cGKI; gene name *Prkg1*) that is essential for the bifurcation of DRG neurons upon entering the spinal cord during embryonic development. In mice deficient for any of these components the axons of DRG neurons no longer bifurcate and instead turn either in only an ascending or descending direction. However, the impact of axon bifurcation on sensory processing in adulthood remains poorly understood.

**Methods:** Since the analysis of global GC-B as well as cGKI mouse knockout mutants is hampered by their decreased survival rates, we generated conditional mouse mutants of GC-B and cGKI (*GC*-*B*^*fl/fl*^;*Wnt1*^*Cre*^ and *cGKI*^*KO/fl*^;*Wnt1*^*Cre*^) that lack sensory axon bifurcation in the absence of additional phenotypes observed in the global knockout mice, to investigate the functional consequences of impaired axon bifurcation during adult stages.

**Results:** Cholera toxin labeling in digits of the hind paw demonstrated an altered shape of sensory neuron termination fields in the spinal cord of conditional GC-B mouse mutants. Behavioral testing indicated that noxious heat sensation and nociception induced by chemical irritants are impaired in the mutants, whereas responses to cold sensation, mechanical stimulation and motor coordination are not affected (1). Recordings from C-fiber nociceptors in the hind limb skin showed that GC-B function is not required to maintain normal heat sensitivity of peripheral nociceptors. Thus the altered behavioral response to noxious heat found in *GC*-*B*^*fl/fl*^;*Wnt1*^*Cre*^ mice does not result from an impaired C-fiber function.

**Conclusions:** Overall, these data point to a critical role of axonal bifurcation for the processing of pain induced by heat or chemical stimuli (Fig. 1).

**Fig. 1:**
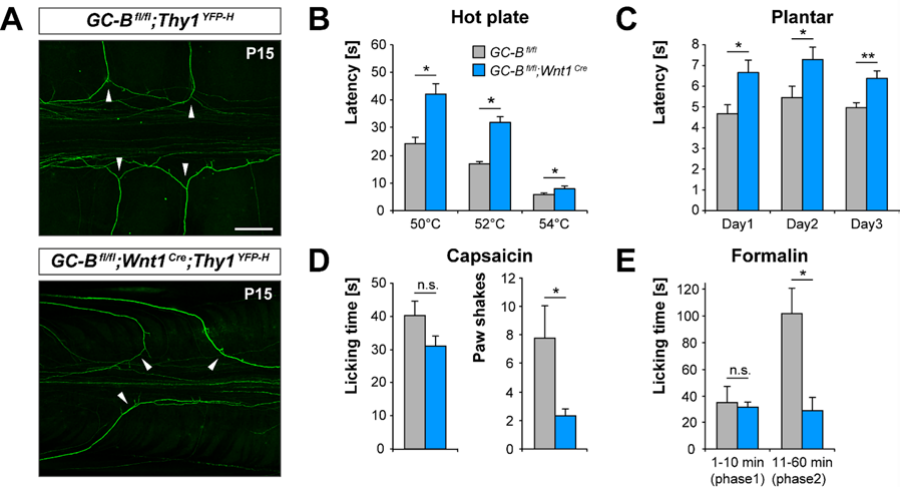
Reduced nocifensive behavior of a conditional GC-B mouse mutant lacking sensory axon bifurcation. **a** Dorsal views of spinal cords from *GC*-*B*^*fl/fl*^;*Thy1*^*YFP*−*H*^ and *GC*-*B*^*fl/fl*^*;Wnt1*^*Cre*^*;Thy1*^*YFP*−*H*^ show a loss of axon bifurcation in the conditional mutant that persisted at mature stages (P15). Scale bar, 250 μm. *GC*-*B*^*fl/fl*^*;Wnt1*^*Cre*^ conditional mouse mutants show a decrease in (**b**) thermal heat pain assessed on a 50 °C, 52 °C, and 54 °C hot plate (n = 15–16; *p < 0.05), **c** thermosensation measured by the Hargreaves’ method (Plantar test) at 3 consecutive days (n = 8; *p < 0.05 and **p < 0.01) and chemical pain response to hindpaw injection of **e** 5 μg capsaicin (n = 9) or **f** 0.5% formalin (n = 5) *p < 0.05

**Acknowledgement:** This work was supported by the DFG (SFB 665 grants B2 to FR and B6 to GL, and FOR 2060 grants FE 438/5-1 and FE 438/6-1 to RF, SCHM 2371/1 to HS, and SCHM 2629/3-1 to AS).

**Reference**

1. Troster P, Haseleu J, Petersen J, Drees O, Schmidtko A, Schwaller F, Lewin GR, Ter-Avetisyan G, Winter Y, Peters S, et al. The absence of sensory axon bifurcation affects nociception and termination fields of afferents in the spinal cord. Front Mol Neurosci. 2018;11:19.

## P40 Specific functions of guanylyl cyclase-B in pain processing

### Lea Kennel^1^, Jörg Isensee^2^, Jonas Petersen^1^, Tilman Groß^1^, Cathrin Flauaus^1^, Ruirui Lu^1^, Julia Adler^3^, Peter Ruth^3^, Tim Hucho^2^, Robert Lukowski^3^, Hannes Schmidt^4^, Achim Schmidtko^1^

#### ^1^Goethe-Universität Frankfurt am Main, Institut für Pharmakologie und Klinische Pharmazie, Frankfurt am Main Hesse, Germany; ^2^Universität Köln, Experimentelle Anästhesiologie und Schmerzforschung, Köln North Rhine-Westphalia, Germany; ^3^Universität Tübingen, Institut für Pharmazie, Tübingen Baden-Württemberg, Germany; ^4^Universität Tübingen, Interfakultäres Institut für Biochemie, Tübingen Baden-Württemberg, Germany

##### **Correspondence:** Achim Schmidtko - schmidtko@em.uni-frankfurt.de

*Journal of Translational Medicine* 2019, **17(2):**P40

**Introduction:** A large body of evidence indicates that cGMP contributes to the processing of pain. While many previous studies focused on NO-dependent cGMP signaling in the nociceptive system, the role of natriuretic peptide-induced cGMP production in this context is poorly understood. Therefore, we here analyzed the distribution of natriuretic peptide receptors in the nociceptive system and assessed a possible function in pain processing using tissue-dependent conditional knockout mice.

**Methods:** The expression pattern of guanylyl cyclase-A (GC-A), guanylyl cyclase-B (GC-B) and potential upstream/downstream effectors in dorsal root ganglia (DRG) and the spinal cord of mice was investigated by in situ hybridization combined with immunhistochemistry using established markers and LacZ reporter mice. Knockout mice with tissue-specific deletion of GC-B were analyzed in animal models of acute nociceptive, inflammatory and neuropathic pain.

**Results:** We found that GC-B, but not GC-A is expressed in subpopulations of DRG neurons and co-expressed with cGMP-dependent protein kinase Iα (cGKIα). Our behavioral data revealed that GC-B deficiency is associated with altered response to noxious stimuli.

**Conclusions:** These data suggest a contribution of CNP/GC-B/cGMP/cGKIα signaling to the processing of pain. Further investigations are necessary to clarify the downstream mechanisms of this pathway.

**Acknowledgement:** This work was supported by the Deutsche Forschungsgemeinschaft (FOR2060 project SCHM2629/3-1).

## P41 Sustained PKG1-signalling causes pathological alterations of the heart

### Gerburg Schwaerzer^1,3^, Darren Casteel^1^, Nancy Dalton^1^, Yusu Gu^1^, Alice Zemljic-Harpf^2^, Dianna Milewicz^4^, Kirk Peterson^1^, Hemal Patel^2^, Renate Pilz^1^

#### ^1^University of California San Diego, Department of Medicine, La Jolla California, US; ^2^University of California San Diego, Department of Anesthesiology, San Diego California, US; ^3^Christian-Albrechts-University Kiel, Department of Medicine, Kiel Schleswig–Holstein, Germany; ^4^University of Texas Health Science Center at Houston, Department of Internal Medicine at McGovern Medical School, Houston Texas, US

##### **Correspondence:** Gerburg Schwaerzer - gschwaerzer@gmx.de

*Journal of Translational Medicine* 2019, **17(2):**P41

**Introduction:** The mutation *PRKG1*-*R177Q* is found in families suffering from thoracic aortic aneurysms and dissections. The exchange of arginine 177 to glutamine causes constitutive activation of PKG1 in the absence of cGMP [1, 2]. Homo- and heterozygous knock-in mice carrying the *PRKG1*-*R177Q* mutation are born with normal Mendelian frequencies, but about 50% of the homozygous mice (*Prkg1*^RQ/RQ^) die within the first 6 weeks with signs of gastrointestinal dysfunction. Here we studied the cardiac phenotype of heterozygous mice (*Prkg1*^RQ/+^) to determine the effects of sustained PKG1 signalling in the heart.

**Methods:** *Prkg1*^RQ/+^ mice and their wild type littermates were studied before and after pressure overload. Cardiac function and structure were assessed by echocardiography, telemetry and histology.

**Results:** Although heart rate and blood pressure were similar between *Prkg1*^RQ/+^ mice and their wild type littermates, the *Prkg1*^RQ/+^mice developed pathological alterations of the heart at the age of 12 months, with prominent mitophagy and hypertrophy of cardiac myocytes. Young *Prkg1*^RQ/+^mice exposed to pressure overload—either from angiotensin II-induced hypertension or from transverse aortic constriction—developed more severe contractile dysfunction and cardiac chamber dilatation compared to their wild type littermates. These changes were partly rescued by antioxidant treatment.

**Conclusions:** Here we show that sustained PKG1-signaling predisposes to stress-induced cardiac dysfunction.

**Acknowledgement:** We thank S. Lee and S. Zhuang for technical assistance. This work was supported by Deutsche Forschungsgemeinschaft (GS) and the National Institutes of Health grants R01-HL132141 (RP) and P30-NS047101 (UCSD Microscopy Shared Facility).

**References**

1. Guo DC, et al. Recurrent gain-of-function mutation in PRKG1 causes thoracic aortic aneurysms and acute aortic dissections. Am J Hum Genet. 2013;93:398–404.
2. Gago-Diaz M, et al. PRKG1 and genetic diagnosis of early-onset thoracic aortic disease. Eur J Clin Invest. 2016;46:787–94.

## P42 A novel model system for abnormal ejaculation shows that the alpha1-adrenoceptor antagonist tamsulosin but not the PDE5 inhibitor tadalafil leads to dysfunction of prostate, seminal vesicles and epididymis

### Mathias Seidensticker^1^, Sabine Tasch^1^, Andrea Mietens^1^, Betty Exintaris^2^, Ralf Middendorff^1^

#### ^1^Justus-Liebig University, Institute of Anatomy and Cell Biology, Giessen Hesse, Germany; ^2^Monash University, Monash Institute of Phamacy and Phamaceutical Sciences, Melbourne, Australia

##### **Correspondence:** Mathias Seidensticker - mathias.seidensticker@anatomie.med.uni-giessen.de

*Journal of Translational Medicine* 2019, **17(2):**P42

**Introduction:** Structures responsible for the emission phase of ejaculation are the seminal vesicles, the most distal part of the cauda epididymidis and the newly characterized prostate excretory ducts (Kügler, Mietens, Seidensticker et al., FASEB J 2018). Pharmacological improvement of symptoms associated with benign prostatic hyperplasia (BPH) include reagents known to reduce the tone of smooth muscle tissue such as alpha1-adrenergic antagonists (e.g. tamsulosin) or the newly approved inhibitor of the cGMP-dependent phosphodiesterase type 5 (PDE5) (tadalafil). Treatment with tamsulosin often leads to severe side effects like abnormal ejaculation. Underlying mechanisms of this disturbance with alpha1-blockers remain unclear and are unknown for the PDE5 inhibitor tadalafil. A differentiated analysis will help clinicians directly to guide and optimize their treatment choices for BPH patients.

The objective is to visualize and compare effects of tamsulosin and tadalafil pre-treatment on noradrenaline-induced contractility, thus imitating the therapeutic situation in the relevant muscular structures of the male reproductive tract responsible for the emission phase of ejaculation and the sympathetic contribution to the emission phase.

**Methods:** Imitating the therapeutic situation, prostate ducts, seminal vesicles and the distal cauda epididymal duct were pre-incubated with the BPH drugs tadalafil, an inhibitor of the phosphodiesterase PDE5, and tamsulosin in a novel ex vivo-time-lapse imaging approach, Afterwards, noradrenergic responses in the relevant structures were investigated.

**Results:** Noradrenaline induced strong contractions ultimately leading to secretion in structures without pre-treatment but were abolished with tamsulosin pre-treatment in prostate ducts and seminal vesicles and significantly decreased in the epididymal duct. These adverse effects were not observed with tadalafil.

**Conclusions:** Data of our model system reveals a serious dysfunction of each organ involved in emission by affecting alpha1-adrenergic receptors, but not by (sympathetic nervous system-independent) targeting of smooth muscle cell-localized PDE5. This new knowledge translates directly to clinical medicine. PDE5 inhibitors like tadalafil are a good alternative therapy in BPH for patients experiencing abnormal ejaculation due to treatment with tamsulosin.

**Reference**

- Kügler R, Mietens A, Seidensticker M, Tasch S, Wagenlehner FM, Kaschtanow A, Tjahjono Y, Tomczyk CU, Beyer D, Risbridger GP, Exintaris B, Ellem SJ, Middendorff R. Novel imaging of the prostate reveals spontaneous gland contraction and excretory duct quiescence together with different drug effects. FASEB J. 2018;32(3):1130–8. 10.1096/fj.201700430r. **(Epub 2018 Jan 3)**.

## P43 sGC stimulation improves memory function and reduces plaque load in a mouse model of Alzheimer’s disease

### Burcu Seker^1^, Damian Brockschnieder^2^, Lisa Dietz^2^, Peter Sandner^2^, Nikolaus Plesnila^1,3^

#### ^1^Institute for Stroke and Dementia Research (ISD), University of Munich Medical Center, Experimental Stroke Research, Munich Bavaria, Germany; ^2^Bayer AG, Research & Development, Pharmaceuticals, Wuppertal North Rhine-Westphalia, Germany; ^3^Munich Cluster of Systems Neurology (Synergy), Munich Bavaria, Germany

##### **Correspondence:** Burcu Seker - burcu.seker@med.uni-muenchen.de

*Journal of Translational Medicine* (2019), 17(2)

**Introduction:** Alzheimer’s disease (AD) is a neurodegenerative disorder characterized by loss of memory and cognitive function. Nitric oxide/soluble guanylate cyclase/cyclic guanosine monophosphate (NO-sGC-cGMP) signaling, which is important for synaptic transmission and vascular tone, was found to be reduced in AD patients and to be correlated with memory decline [1]. Since NO-sGC-cGMP signaling can be increased by application of specific sGC stimulators by more than 20-fold [2], we hypothesized that sGC stimulation may have a therapeutic potential in AD. Therefore, the aim of the current study was to investigate the effect of sGC modulation on memory function and amyloid beta (Aβ) plaque load in a murine AD model.

**Methods:** 5XFAD transgenic mice or their wild-type littermates were randomly and blindly allocated to the following groups: wild type (wt), 5xFAD + vehicle and 5xFAD + drug (n = 11/each group). BAY 41-2272, a sGC stimulator, was administered p.o. (45 mg/kg). Feeding was initiated when the mice were 2-month old and was continued for 10 months. Learning and memory were analyzed monthly using a Barnes maze paradigm. After sacrifice, Aβ plaque load was quantified by Thioflavine-S and Congo Red staining and by ELISA measurements.

**Results:** Application of the sGC stimulator showed no effect on body weight or other physiological parameters. As expected, 5xFAD mice receiving vehicle developed memory and learning deficits (p < 0.001). Pharmacological activation of sGC significantly improved memory function and learning at 6 (p < 0.05), 7 (p < 0.01), and 8 (p < 0.05) months of age. In parallel, the Aβ plaque area fraction was significantly reduced in the drug treated group as compared to vehicle treated controls (p < 0.05).

**Conclusions:** Our results show that pharmacological sGC stimulation has beneficial effects on memory function and plaque load in a murine AD model. Thus, NO-sGC-cGMP signaling seems to play an important, so far little recognized, role in the pathophysiology of AD. Further experiments need to clarify the precise connection between NO-sGC-cGMP signaling and Aβ plaque formation and the molecular interactions between the sGC-cGMP pathway and memory function.

**References**

- Hesse R, Lausser L, Gummert P, Schmid F, Wahler A, Schnack C, Kroker KS, Otto M, Tumani H, Kestler HA, Rosenbrock H, von Arnim CA. Reduced cGMP levels in CSF of AD patients correlate with severity of dementia and current depression. Alzheimers Res Ther. 2017;9(1):17
- Stasch JP, Pacher P, Evgenov OV. Soluble guanylate cyclase as an emerging therapeutic target in cardiopulmonary disease. Circulation. 2011;123(20):2263–73.

## P44 Effect of treatment with a new nitric oxide donor, a hybrid derived from resveratrol and furoxan (E)-4-(4-(4-methoxystyryl)phenoxy)-3-methyl-1,2,5-oxadiazole 2-oxide, on priapism in sickle cell mouse and nitric oxide-deficient mouse

### Fabio H. Silva^1^, Beatriz P. B. Godoy^1^, Eduardo C. Alexandre^2^, Fabiano B. Calmasini^2^, Jean L. dos Santos^3^, Fernando F. Costa^1^

#### ^1^University of Campinas, Hematology and Hemotherapy Center, Campinas, Brazil; ^2^University of Campinas, Department of Pharmacology, Campinas, Brazil; ^3^UNESP, Laboratório de Pesquisa e Desenvolvimento de Fármacos, Araraquara, Brazil

##### **Correspondence:** Fabio H. Silva - fabiohsilva87@gmail.com

*Journal of Translational Medicine* 2019, **17(2):**P44

**Introduction:** Patients with sickle cell disease (SCD) display priapism. The low nitric oxide (NO)-cGMP bioavailability and increased oxidative stress has been reported to play an important role in the pathophysiology of priapism in SCD. Therefore, we aimed to evaluate the effects of new NO donor, a hybrid derived from resveratrol and furoxanic, (E)-4-(4-(4-methoxystyryl) phenoxy)-3-methyl-1,2,5-oxadiazole 2-oxide compound RVT-FxMe, on functional alterations of erectile function in murine models that display low NO-cGMP bioavailability, SCD transgenic mice and endothelial NO synthase gene-deficient (eNOS^−/−^) mice. We have focused on the dysregulated NO-cGMP in erectile tissue of SCD and eNOS^−/−^ mice.

**Methods:** Wild type (WT, C57BL/6), Townes SCD transgenic and eNOS^−/−^ male mice (3–5 month old) were treated with compound RVT-FxMe (25 mg/kg/day) or its vehicle (20% Cremophor) daily for 2 weeks via I.P.. Corpus cavernosum (CC) strips were mounted in isolated organ baths, and the relaxation responses to acetylcholine (ACh; 0.001–3 µM; endothelium-dependent response) and sodium nitroprusside (SNP; 0.001–30 µM; endothelium-independent response), as well as electrical-field stimulation (EFS, 2–32 Hz; nitrergic relaxation) were obtained in CC strips precontracted with the α1-adrenoceptor agonist phenylephrine (3–10 µM).

**Results:** Hematological parameters (red blood cells [× 10^−6^/μL] and hemoglobin [g/dL]) show that SCD (5.7 ± 0.6 and 6.3 ± 0.3) have severe anemia compared to WT (8.8 ± 0.1 and 12.4 ± 0.1) and eNOS^−/−^ mice. Plasma hemoglobin was higher (P < 0.05) in SCD compared to control and eNOS^−/−^ mice. Mouse CC relaxations to SNP and EFS (16 Hz) were increased (P < 0.05) in eNOS^−/−^ (107 ± 5% and 96 ± 5%) compared to WT group (87 ± 5% and 71 ± 4%, respectively), which were normalized by compound RVT-FxMe treatment (n = 5). In SCD group, ACh and SNP produced CC relaxations in WT and SCD groups, but the ACh and SNP maximal response (Emax) values were higher (P < 0.05) in CC of SCD compared to WT mice. Similarly, the nitrergic relaxation induced by EFS was also higher (P < 0.05) in SCD mice compared to WT mice (4 Hz: 41 ± 6% and 63 ± 4%, respectively; n = 5). Compound RVT-FxMe treatment did not change relaxation response to ACh, SNP and EFS in CC from SCD and WT mice (n = 5).

**Conclusions:** Compound RVT-FxMe treatment reversed enhanced NO-cGMP-mediated CC relaxations in eNOS^−/−^ mice, without changing in SCD mice. It is likely that excess of plasma hemoglobin in CC of SCD mice act to inactivate NO before it reaches soluble guanylyl cyclase. One may speculate that the amount of nitric oxide donated by this compound was not sufficient to restore the NO-cGMP bioavailability in CC from SCD mice.

**Acknowledgement:** São Paulo Research Foundation (FAPESP; 2017/08122-9 and 2018/06243-6).

## P45 Paracrine effects of cardiac natriuretic peptides on pericytes: role in adaptative angiogenesis

### Marie Sezen Rüsing^1,2^, Lisa Krebes^1,2^, Marco Abeßer^1^, Hideo A. Baba^3^, Michaela Kuhn^1,2^, Katarina Špiranec Spes^1,2^

#### ^1^University of Würzburg, Institute of Physiology, Würzburg Bavaria, Germany; ^2^University Hospital Würzburg, Comprehensive Heart Failure Center, Würzburg Bavaria, Germany; ^3^University Hospital Essen, Institute of Pathology, Essen North Rhine-Westphalia, Germany

##### **Correspondence:** Katarina Špiranec Spes - katarina.spiranec@uni-wuerzburg.de

*Journal of Translational Medicine* 2019, **17(2):**P45

**Introduction:** Hypertensive cardiac hypertrophy develops as adaptative response to chronically increased afterload. Cardiac hypertrophy is initially associated with an increased myocardial capillary density, whereas at later stages capillary rarefaction may occur (1). In cardiac pressure/volume overload, natriuretic peptide expression and secretion rapidly increase. NPs are known for vascular and renal actions, essential for the physiological maintenance of intravascular volume and arterial blood pressure. Moreover, they exert direct local, cardiac antihypertrophic and antifibrotic actions (2). In addition, endothelial effects of NPs were shown to enhance angiogenesis and neovascularization (3). Herein we studied whether NP/GC-A/cyclic GMP signaling modulates adaptative myocardial angiogenesis during early stages of experimental cardiac hypertrophy.

**Methods:** To study GC-A-mediated microcirculatory actions of ANP in vivo, we selectively inactivated the GC-A gene in pericytes by tamoxifen-induced *loxP/PDGFRβ*-*Cre*^*ERT2*^-mediated recombination. In pericyte GC-A KO mice, cardiac structure and function were explored in an experimental model of transverse aortic constriction (TAC) induced hypertensive heart disease. To evaluate cardiac contractile functions, aortic and LV pressure–volume relationships were recorded. Cardiac cellular remodelling was studied by immunocytochemistry and morphometrical analyses.

**Results:** LV contractile and relaxation functions of TAC-operated mice were mildly impaired, as evidenced by genotype-independent changes in LV and aortic pressures and contraction/relaxation rates. Morphometry of cardiac sections co-stained with anti-NG2, anti-PDGF-Rβ and anti-CD31 antibodies indicated mild decreases of cardiac pericyte densities (by 5%) and significant decreases of myocardial capillaries (by 15%) in the pericyte GC-A KO mice already under baseline conditions. A significant increase in pericyte and capillary densities was observed in both genotypes 7 days after TAC. Of note, the density of cardiac pericytes (by 5%) and myocardial capillaries (by 15%) remained diminished in pericyte GC-A KO mice after TAC, indicating that loss of GC-A/cGMP signaling alters pericyte proliferation and/or viability and thereby the communication from pericytes to endothelial cells. Indeed, our studies of the pericyte proliferation in real time demonstrated that ANP simulates pericyte proliferation in a concentration-dependent manner.

**Conclusions:** Our observations demonstrate that inhibition of pericyte NP/GC-A/cGMP signaling diminishes pericyte proliferation, promotes microvascular rarefaction, and thereby possibly the transition from compensated hypertensive cardiac hypertrophy to heart failure.

**Acknowledgement:** This study was supported by the Deutsche Forschungsgemeinschaft (DFG KU 1037/7-1 and KU 1037/8-1 to MK) and the Bundesministerium für Forschung und Technik (BMBF 01EO1004 to MK and BMBF 01EO1504 to KSS).

**References**

- Hein S, et al. Progression from compensated hypertrophy to failure in the pressure-overloaded human heart: structural deterioration and compensatory mechanisms. Circulation. 2003;107:984–99.
- Kuhn M. Molecular physiology of membrane guanylyl cyclase receptors. Physiol Rev. 2016;96:751–803.
- Kuhn M, et al. The natriuretic peptide/guanylyl cyclase-a system functions as a stress-responsive regulator of angiogenesis in mice. J Clin Invest. 2009;119:2019–30.

## P46 Mechanisms that insure creation of an NO-responsive sGC heterodimer

### Dennis Stuehr^1^, Yue Dai^1^, Arnab Ghosh^1^, Saurav Misra^2^

#### ^1^Cleveland Clinic, Lerner Research Institute, Cleveland Ohio, US; ^2^Kansas State University, Dept. Biochemistry & Molecular Biophysics, Manhattan Kansas, US

##### **Correspondence:** Dennis Stuehr - stuehrd@ccf.org

*Journal of Translational Medicine* 2019, **17(2):**P46

**Introduction:** How heme insertion occurs during sGC maturation and leads to formation of an NO-responsive sGCα/β heterodimer is unclear. Early studies established that sGC associates with heat shock protein 90 (hsp90) in cells and tissues and this usually correlated with an increase in sGC activity. More recently we determined that (i) in cells and tissues, hsp90 associates with only the heme-free apo-form of sGCβ, and not with holo-sGCβ, sGCα, or the sGCα/β heterodimer, and (ii) purified apo-sGCβ can interact directly with hsp90 to form a complex. We utilized interaction mapping based on our HxD MS and biophysical measures, existing structural data, and computer-assisted docking, to build an evidence-based model of an apo-sGCβ-hsp90 interaction complex. In this model, apo-sGCβ1(1-358) binds through its PAS domain to the middle domain of one protomer of an hsp90 homodimer. In the present study, we sought to determine if a complex corresponding to our model actually forms in mammalian cells, and if so, to define its biological relevance in regulating sGCβ heme insertion, sGCα/β heterodimer formation, and catalytic activity.

**Methods:** We generated sGCβ variants that contain model-based point and deletion mutations predicted to disrupt the apo-sGCβ-hsp90 interaction in three specific regions in sGCβ. We used fluorescence polarization and antibody immuno-precipitation (co-IP) to measure each sGCβ variant’s interaction with hsp90 both in vitro and in cultured mammalian cells. We utilized a FIash-based fluorophore tag method to monitor heme insertion into each sGCβ variant in live cells, and used co-IP to determine their heterodimer formation with sGCα, and a kit to measure cyclase activity.

**Results:** Most of the model-guided mutations either partly or completely inhibited sGCβ-hsp90 complex formation, both in purified form and when the proteins were expressed in mammalian cells. The degree of their hsp90 complex formation closely correlated with the extent of their heme insertion in mammalian cells, and with the development of an NO-activatable cyclase activity. Several apo-sGCβ variants that could no longer bind hsp90 could now bind an sGCα partner in cells to form a heme-free sGCα/β heterodimer.

**Conclusions:** Heme-free sGCβ and hsp90 do form a direct complex in mammalian cells that mimicks the molecular modeled structure.

Complex formation is needed to drive heme insertion into the β-subunit, and to ultimately create an NO-responsive sGCα/β heterodimer.

Hsp90 binding to apo-sGCβ also prevents a premature association with sGCα, and thus prevents formation of a heme-free sGCα/β heterodimer in cells.

Hsp90 directs sGC maturation by two mechanisms (Fig. 1).

**Fig. 1:**
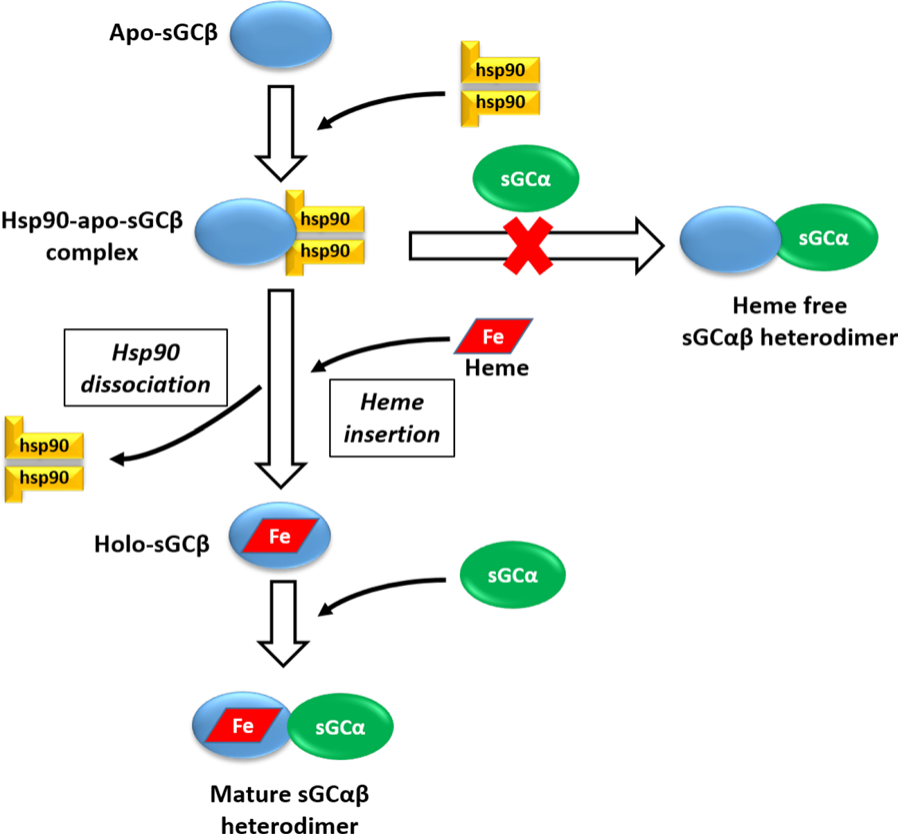
Dual regulation of sGC maturation by hsp90 in mammalian cells. When hsp90 binds to apo-sGCβ it drives heme insertion while also preventing a premature interaction with sGCα to form a heme-free, non-functional sGCα/β heterodimer. Once heme insertion is complete, the hsp90 dissociates from sGCβ, and this allows sGCβ to partner with sGCα to form a mature and NO-responsive sGCα/β heterodimer

## P47 Differences in the response to BAY 60-2770 among various isolated monkey arteries

### Masashi Tawa^1^, Tomio Okamura^2^, Takayoshi Masuoka^1^, Matomo Nishio^1^, Takaharu Ishibashi^1^

#### ^1^Kanazawa Medical University, Department of Pharmacology, Kahoku, Japan; ^2^Shiga University of Medical Science, Department of Pharmacology, Otsu, Japan

##### **Correspondence:** Masashi Tawa - tawa@kanazawa-med.ac.jp

*Journal of Translational Medicine* 2019, **17(2):**P47

**Introduction:** Little information is available concerning the heterogeneity in the vasorelaxing effects of sGC activators. This study was, therefore, undertaken to compare the effects of the sGC activator, BAY 60-2770, on different arteries.

**Methods:** Middle cerebral, left circumflex coronary, intrarenal, and mesenteric branch arteries were isolated from 8 female crab-eating monkeys (*Macaca irus*) weighing 3–6 kg. The arteries (0.7–1.0 mm outside diameter) were cut helically into strips and used in organ chamber experiments as described previously [1]. Briefly, the strips, in which the resting tension was adjusted to 1.0 g, were partially contracted using prostaglandin F_2α_, and concentration–response curves for BAY 60-2770 were obtained. Relaxation (%) of the arteries was presented relative to the relaxation induced by 10^−4^ M papaverine. Concentration–response curves were analyzed using the two-way repeated measures analysis of variance (ANOVA) and Bonferroni’s post hoc test. The maximal response (E_max_) and the negative logarithm of the dilator concentration that produces half E_max_ (pEC_50_) were compared using Bonferroni’s multiple comparisons test after one-way ANOVA.

**Results:** BAY 60-2770 induced concentration-dependent relaxation of the cerebral, coronary, renal, and mesenteric arteries; however, the responsiveness differed for each artery (Fig. 1). In addition, E_max_ values for cerebral (95.5 ± 1.5%) and coronary arteries (90.7 ± 3.0%) were significantly higher than the values for renal (73.1 ± 5.2%, p < 0.01 vs. cerebral; p < 0.05 vs. coronary) and mesenteric arteries (75.4 ± 4.0%, p < 0.01 vs. cerebral; p < 0.05 vs. coronary). In contrast, there was no significant difference in pEC_50_ values (cerebral, 9.26 ± 0.12; coronary, 9.12 ± 0.17; renal, 8.73 ± 0.14; mesenteric, 9.04 ± 0.12).

**Fig. 1:**
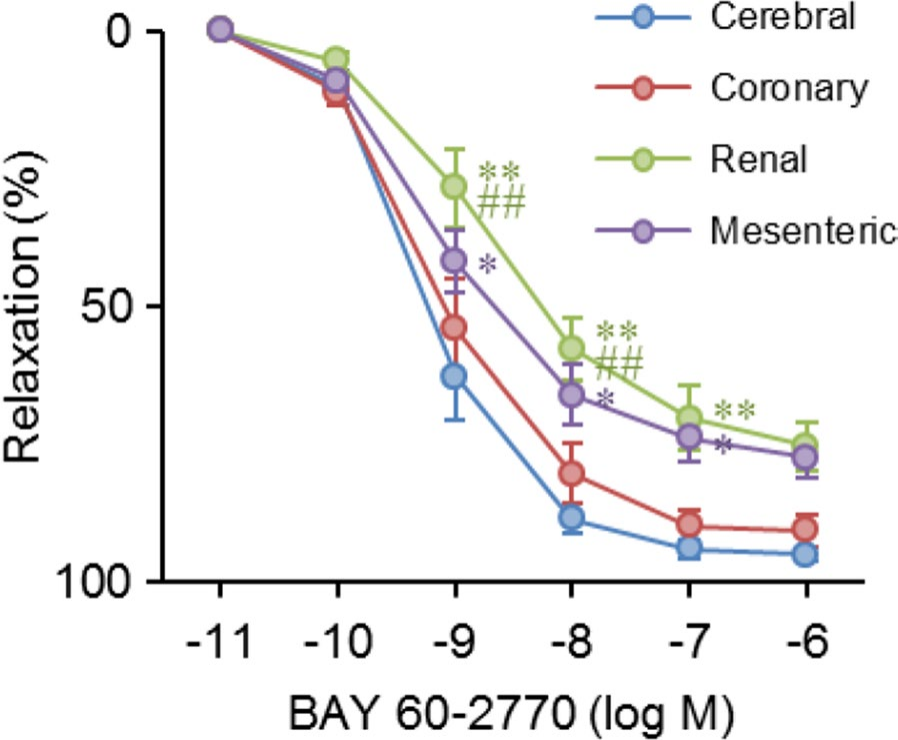
Vasorelaxant responses of monkey arteries to BAY 60-2770. Each point and bar represents the mean ± SEM of 8 experiments. *p < 0.05 and **p < 0.01, compared with the cerebral artery; ^##^p < 0.01, compared with the coronary artery

**Conclusions:** The relaxant response to BAY 60-2770 was greater for the cerebral and coronary arteries than that for the renal and mesenteric arteries. Interestingly, the arteries that supply blood to organs with high oxygen consumption seem to be more sensitive to sGC activators. This may be due to such arteries being exposed to relatively high oxidative stress conditions [2].

**References**

1. Tawa M, Okamura T: Soluble guanylate cyclase redox state under oxidative stress conditions in isolated monkey coronary arteries. Pharmacol Res Perspect. 2016;4(5):e00261.
2. Miller AA, Drummond GR, Schmidt HH, Sobey CG. NADPH oxidase activity and function are profoundly greater in cerebral versus systemic arteries. Circ Res. 2005;97(10):1055–62.

## P48 S-palmitoylation of GC1α mediates its caveolae-localization in cardiac myocytes

### Christos Tzimas^1^, Sevde Yilmaz^2^, Sudarsan Ranjan^2^, Emily J. Tsai^1^

#### ^1^Columbia University Vagelos College of Physicians & Surgeons, Division of Cardiology, New York New York, US; ^2^Lewis Katz School of Medicine at Temple University, Center for Translational Medicine, Philadelphia Pennsylvania, US

##### **Correspondence:** Emily J. Tsai - et2509@cumc.columbia.edu

*Journal of Translational Medicine* 2019, **17(2):**P48

**Introduction:** In cardiac myocytes, caveolae-localization of GC-1 confers relatively enhanced NO-responsiveness and protection against oxidation [1]. Strikingly, caveolae-localization of GC-1 is dynamic. In cardiac pressure- or volume-overloaded animals, GC-1 re-localized away from caveolae, with subsequent oxidation and blunted NO-responsiveness in the hypertrophied heart [1–3]. How GC-1 dynamically localizes to caveolae is unknown. S-palmitoylation is a reversible, enzyme-catalyzed, post-translational modification that enhances membrane affinity of protein substrates. We hypothesized that s-palmitoylation mediates GC-1 localization to caveolae and thereby modulates redox regulation of GC-1 in cardiac myocytes.

**Methods:** To determine whether GC-1 is differentially s-palmitoylated in the hypertrophied heart, C57BL/6 mice were subjected to transverse aortic constriction (TAC) vs thoracotomy (Sham). S-palmitoylation was assessed by modified acyl-biotin exchange (ABE). Using computational prediction algorithms, we identified 5 conserved cysteine residues on GC1α as possible S-palmitoylation sites. We generated human GC1α mutants with serine substitution of each and all of the 5 cysteines (C3S, C15S, C176S, C497S, C595S, C-S*) and expressed the mutants and WT GC1α in neonatal rat cardiomyocytes (NRCMs) using adenoviral mediated gene transfer. We assessed s-palmitolyation of these constructs by ABE. Caveolae-localization of C-S* vs WT GC1α was assessed by confocal microscopy and immunoselection of plasmalemmal caveolae.

**Results:** GC1α was S-palmitoylated in Sham but far less so in TAC hearts. GC1β was not S-palmitoylated in either Sham or TAC hearts. In transfected NRCMs, s-palmitolyation of the GC1α C-S* mutant was profoundly diminished (6.9 ± 2.0%), while that of the C15S, C176S, and C497S mutants were modestly diminished (respectively 23.8 ± 8.4%, 23.2 ± 6.6%, and 22.8 ± 6.0%) compared to GC1αWT (54.7 ± 8.7%, normalized to S-palmitoylation of eNOS). C3S and C595S mutants were s-palmitoylated at levels similar to WT. Confocal microscopy of transfected NRVMs revealed more diffuse cytosolic distribution of C-S* mutant compared to GC1αWT. Immunoblot of immunoselected plasmalemmal caveolae from C-S* transfected NRVMs demonstrated less GC1 co-localization compared to WT.

**Conclusions:** Our data suggest that S-palmitoylation of GC1α at C15, C176, and C497 mediates GC1 caveolae-localization. S-palmitoylation of GC1α at these 3 cysteines may be important in reciprocal cysteine post-translational regulation of GC1 localization and activity. We are in the process of determining the redox state and inducible cyclase activity of the S-palmitoylation-resistant GC1α mutant.

**References**

1. Tsai EJ, et al. Circ Res. 2012;110(2):295–303.
2. Liu Y, et al. J Mol Cell Cardiol. 2013;60:72–83.
3. Trappanese D, et al. Basic Res Cardiol 2015;110(1):456.

## P49 Inhibition of cyclic GMP export by multidrug resistance protein 4: a new strategy to treat erectile dysfunction?

### Johan Van de Voorde, Charlotte Boydens, Bart Pauwels, Laura Vanden Daele

#### Ghent University, Department of Basic and Applied Medical Sciences, Vascular Research Unit, Ghent, Belgium

##### **Correspondence:** Johan Van de Voorde - johan.vandevoorde@ugent.be

*Journal of Translational Medicine* 2019, **17(2):**P49

**Introduction:** Elevation of cyclic guanosine monophosphate (cGMP) levels is a key event for normal penile erection. Intracellular cGMP concentrations are regulated by degradation enzymes (phosphodiesterases, PDEs) as well as by active transport across the plasma membrane by multidrug resistance protein (MRP) 4 and 5. This study evaluated the functional effect of MRP 4 inhibition and the role of MRP 4-mediated cGMP export in mouse corpora cavernosa.

**Methods:** Isometric tension of mouse corpora cavernosa was measured after cumulative addition of MK-571, an inhibitor of MRP 4, or sildenafil, a PDE-5 inhibitor. In addition the effect of MRP 4 inhibition on cGMP-(in)dependent relaxations was studied. In vivo intracavernosal pressure (ICP) and mean arterial pressure (MAP) measurements were performed after intracavernosal injection of MK-571.

**Results:** MK-571 and sildenafil both relaxed the corpora cavernosa concentration dependently with sildenafil being the most potent relaxing compound. Furthermore, MK-571 enhanced relaxing responses to cGMP-dependent substances even under in vitro diabetic conditions. In contrast cGMP-independent relaxations were not altered by MRP 4 inhibition. Intracavernosal administration of MK-571 significantly increased ICP, with minimal effect on MAP. cGMP analysis revealed that MRP 4 inhibition was accompanied with increased cGMP levels.

**Conclusions:** This study demonstrates that inhibition of MRP 4 increases basal and stimulated levels of cGMP leading to corpora cavernosa relaxation and penile erection. Therefore it is suggested that, in addition to degradation of cGMP, export of cGMP by MRP 4 substantially contributes to regulating the cGMP levels in mouse corpora cavernosa. As a consequence, MRP 4 might be a valuable alternative target for the treatment of (diabetic) erectile dysfunction.

**Acknowledgement:** Supported by a grant of the Special Investigation Fund of Ghent University (GOA 01G02410).

A version of this abstract was previously published in the Journal of Vascular Research [1].

**Reference**

1. J Vasc Res. 2017;54(S1):PoB-66.

## P50 Raising cyclic GMP activates 26S proteasome function and protein degradation and has therapeutic effects in mouse and zebrafish models of neurodegenerative diseases

### Jordan VerPlank^1^, Sylwia Tyrkalska^3^, Angeleen Fleming^3^, Maria L. Feltri^2^, Lawrence Wrabetz^2^, David Rubinsztein^3^, Alfred Goldberg^1^

#### ^1^Harvard Medical School, Boston Massachusetts, US; ^2^SUNY Buffalo, Buffalo New York, US; ^3^University of Cambridge, Cambridge, UK

##### **Correspondence:** Jordan VerPlank - jordan_verplank@hms.harvard.edu

*Journal of Translational Medicine* 2019, **17(2):**P50

**Introduction:** The Ubiquitin Proteasome System (UPS) degrades most intracellular proteins, including the misfolded proteins that cause neurodegenerative diseases. In this pathway, proteins are tagged with ubiquitin chains and then degraded by the 26S proteasome. Although it is generally believed that degradation rates by the UPS are regulated solely at the level of ubiquitination, our recent studies established that proteasomal activities and degradation of misfolded and regulatory proteins are activated by cAMP and Protein Kinase A (PKA) through phosphorylation of proteasome subunit Rpn6.

We show here that cGMP and Protein Kinase G (PKG) also activate 26S proteasomal activities, but via phosphorylation of a subunit other than Rpn6, and unlike cAMP and PKA, stimulates degradation of the bulk of cell proteins in addition to misfolded and regulatory proteins. 26S proteasomes purified from cultured human neuroblastoma cells, zebrafish, or tissues from mice treated with the phosphodiesterase 5 (PDE5) inhibitor sildenafil exhibit enhanced degradation of polyubiquitinated proteins, ATP, and small peptides. Dephosphorylating the proteasomes from sildenafil-treated cells eliminated the greater activity and incubating control proteasomes with Protein Kinase G (PKG) stimulated their activity. Raising cGMP in several cell lines with PDE5 inhibitors or soluble guanylyl cyclase stimulators also activated proteasomes and the degradation of the bulk of cell proteins by the UPS, without altering the autophagy-lysosome system. Inhibition of PKG, or siRNA-mediated knock down, blocked the cGMP-mediated increase in bulk protein degradation and transiently-overexpressing PKG stimulated it.

Because neurodegenerative diseases are caused by an accumulation of mutant proteins, we tested whether raising cGMP could enhance proteasome function and protein degradation in mouse and zebrafish models of proteotoxic diseases of the nervous system. Sildenafil treatment of zebrafish larvae overexpressing mutant tau (A152T) reduced the accumulation of the mutant proteins and the associated neuronal death and anatomical defects. A 2-week sildenafil treatment of a mouse model of Charcot Marie Tooth 1B, a demyelinating neuropathy caused by expression of mutant *Myelin Protein* Zero which causes an unfolded protein response and proteasome impairment, stimulated proteasomal activity in the affected sciatic nerves, reduced the accumulation of polyubiquitinated proteins, and increased myelin thickness and nerve conduction. Thus, cGMP and PKG are important regulators of proteasome activity and intracellular protein breakdown and agents that raise cGMP have the potential to combat neurodegenerative diseases.

## P51 Anti-proliferative effects of cCMP and cGMP in breast cancer cell lines

### Sabine Wolter, Malte Glüsen, Roland Seifert

#### Medical School Hannover, Institute of Pharmacology, Hannover Lower Saxony, Germany

##### **Correspondence:** Sabine Wolter - wolter.sabine@mh-hannover.de

*Journal of Translational Medicine* 2019, **17(2):**P51

**Introduction:** Breast cancer leads to over 450,000 annual deaths in women world-wide. The classification of breast cancer is based on the overexpression of certain receptors on the cell nucleus such as estrogen (ER) and progesterone (PR) receptors, and amplification of the human epidermal growth factor receptor 2 (HER2) genes. Triple-negative breast cancer (TNBC) lacks ER, PR and HER2 gene amplification (1). The classification is important for the treatment: For ER+ and PR+ breast cancer, receptor-specific therapy is applicable; for TNBC breast cancer, chemotherapy is applied.

The canonical second messenger cAMP and cGMP and also the non-canonical second messenger cCMP and cUMP show diverse effects. cCMP and cUMP induce apoptosis in several cell lines (2, 3).

**Methods:** For our investigations we used the human ER+ MCF-7 and the ER- and PR-MDA-MB-231 (MDA) breast cancer cell line. The second messenger functions of cNMPs were imitated by the membrane-permeable acetoxymethyl ester analogues (cNMP-AMs), which release cNMPs after intracellular hydrolysis. The cell viability was analysed by the alamarBlue assay. Impedance was measured continuously for 120 h by the xCelligence system. Expression of genes, e.g. cGMP-dependent kinase isoforms (PKGIα and PKGII), ER, and PR was analysed by qRT-PCR analysis.

**Results:** Cells showed a cell type-specific expression pattern for ER and PR. MDA cells showed a much lower expression than the MCF-7 cells, whereas PKG isoforms were higher expressed in MDA than in MCF-7 cells.

All cNMP-AMs showed a time- and concentration-dependent anti-proliferative effect in both cells. cCMP-AM was the most potent substance. The results are summarized in Table 1.

**Table 1:** Anti-proliferative effect of cNMP-AMs (100 µM)

MCF-7 (24 h)
cCMP-AM > cGMP-AM > cUMP-AM > cAMP-AM
MCF-7 (48 h)
cCMP-AM > cGMP-AM ~ cAMP-AM > cUMP-AM
MDA (24 h)
cCMP-AM > cGMP-AM > cAMP-AM ~ cUMP-AM
MDA (48 h)
cCMP-AM > cGMP-AM > cAMP-AM > cUMP-AM

Measurement of cell impedance with the xCelligence system showed a cell type- and cNMP-AM-specific pattern (Fig. 1).

**Fig. 1:**
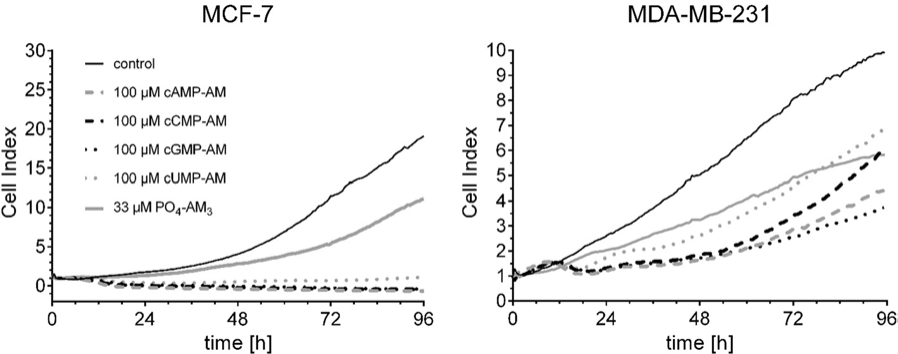
Cell index of MCF-7 and MDA-MB-231 cells. In MCF-7 cells, a decrease of the cell index over up to 120 h was observed, whereas in the MDA-MB-231 cells, the cell index had decreased over the course of 24 h before it finally increased. The control substance PO_4_-AM_3_ showed no effect on the cell index

**Conclusions:** Our data show an anti-proliferative activity of cCMP-AM and cGMP-AM in MCF-7 and MDA cells. Particularly important are the results for the MDA cells, because this cell line has been used as a model for the currently difficult-to-treat form of breast cancer. We thus plan to perform further studies with cCMP- and cGMP-AMs, other membrane-permeable analogues as well as a mixture of cNMP-AMs with chemotherapeutic drugs to analyse the mechanism by which cell proliferation in breast cancer cell lines is reduced. The decrease of the cell index in MDA cells was restricted to 24 h; therefore, repeated replication of cNMP-AMs has to be investigated.

**References**

1. Núñez C, Capelo JL, Igrejas G, Alfonso A, Botana LM, Lodeiro C. An overview of the effective combination therapies for the treatment of breast cancer. Biomaterials. 2016;97:34–50.
2. Wolter S, Kloth C, Golombek M, Dittmar F, Försterling L, Seifert R. cCMP causes caspase-dependent apoptosis in mouse lymphoma cell lines. Biochem Pharmacol. 2015;98:119–31.
3. Dittmar F, Wolter S, Seifert R. Regulation of apoptosis by cyclic nucleotides in human erythroleukemia (HEL) cells and human myelogenous leukemia (K-562) cells. Biochem Pharmacol. 2016;112:13–23.

