# Correction to: Crystal structure of PKG Iβ holoenzyme reveals a trans‑inhibiting dimer assembly

**DOI:** 10.1186/s12967-019-02198-7

**Published:** 2020-01-16

**Authors:** Choel Kim, Rajesh Sharma, Darren E. Casteel

**Affiliations:** 1 Baylor College of Medicine, Pharmacology and Chemical Biology, Houston, TX USA; 2 Baylor College of Medicine, Biochemistry and Molecular Biology, Houston, TX USA; 3 University of California, San Diego, Medicine, La Jolla, CA USA

## Correction to: J Transl Med (2019) 17(Suppl 2):254 10.1186/s12967-019-1994-0

After publication of abstract S 2‑06 in supplement [1], it was brought to our attention that the second author’s name is spelled incorrectly. Originally the author name has been published as Rajesh Sarma. The correct author name is Rajesh Sharma. The full text of abstract S 2-06 with the corrected author list can be found below.

**S 2‑06 Crystal structure of PKG Iβ holoenzyme reveals a trans‑inhibiting dimer assembly**

Choel Kim^1,2^, Rajesh Sharma^1^, Darren E. Casteel^3^

^1^Baylor College of Medicine, Pharmacology and Chemical Biology, Houston, TX, USA; ^2^Baylor College of Medicine, Biochemistry and Molecular Biology, Houston, TX, USA; ^3^University of California, San Diego, Medicine, La Jolla, CA, USA

**Correspondence:** Choel Kim - ckim@bcm.edu

*Journal of Translational Medicine* 2019, **17(2):** S2-06

**Introduction:** As the major molecular switch for regulation of smooth muscle/vascular tone and nociception, mammalian cGMP dependent protein kinase I is a promising therapeutic target for hypertensive diseases and chronic pain. The lack of structural information on the PKG holoenzyme has hindered a detailed understanding of its regulation, though the holoenzyme structure for cAMP dependent protein kinase (PKA) suggests plausible models for PKG regulatory (R) and catalytic (C) domain interactions in the inhibited state.

**Methods:** We determined a crystal structure of PKG Iβ holoenzyme complex at 2.3 Å that enables us to visualize the R–C interface of PKG Iβ of the inhibited state for the first time. Results: We crystallized a monomeric PKG Iβ that lacks the dimerization domain, but the asymmetric unit of the crystal contains a twofold symmetric dimer. The interfaces formed between PKG R and C domains are similar to those seen in the PKA Iα holoenzyme with the inhibitor sequence docked to the active site cleft. However, the overall topology unexpectedly reveals that the R domain of each PKG monomer binds the C domain of the other monomer, giving rise to inhibition in trans (Fig. 1).

**Fig. 1:**
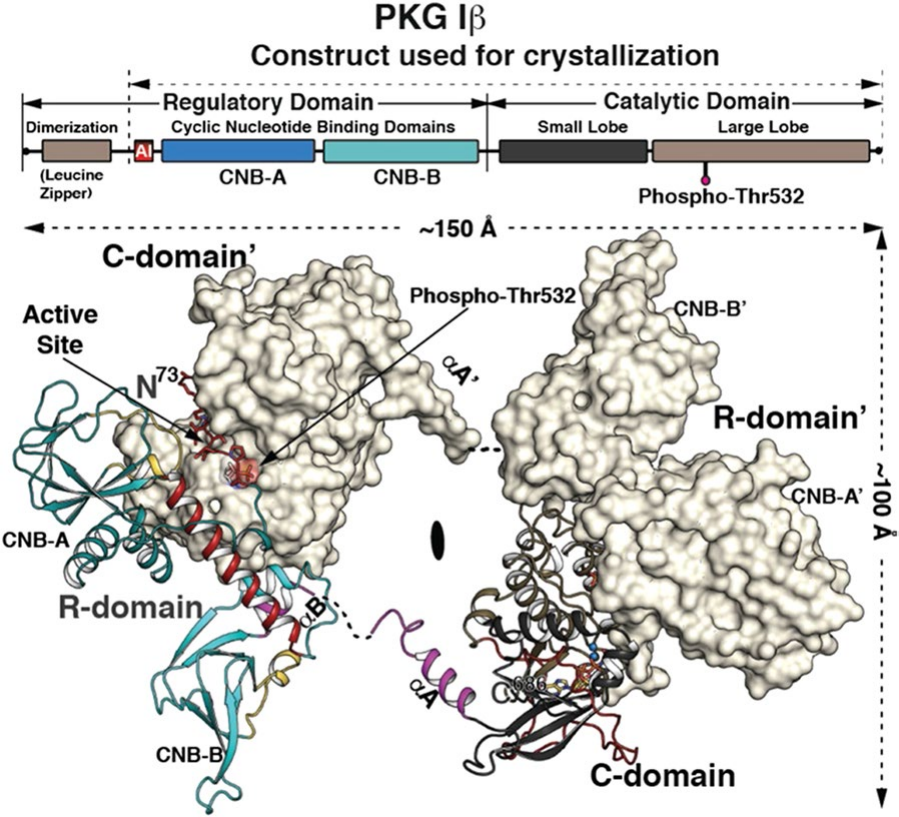
Crystal structure of the PKG Ib holoenzyme complex. The domain organization is shown at the top and the structure of the PKG Ib holoenzyme complex below. The trans-inhibiting dimer is shown with one monomer with surface and the other in cartoon representation. The autoinhibitor (AI) sequence and the interlinking helix between CNB-A and B are colored in red. CNB-A is colored in teal, CNB-B in cyan and PBCs in yellow. The small and large lobes are colored in black and tan respectively. The C-terminal loop is shown in red. The disordered regions between the R and C-domain are shown in dotted lines

**Conclusions:** In the light of previous PKG structural biology, this structure suggests that the PKG inhibited and activated states are stabilized by mutually exclusive domain–domain contacts that either occlude or expose the active site. Our previous structure of the activated state of the isolated PKG Iβ regulatory domain [2] identified a dimer formed by R–R domain interactions (mediated in part by interfacial cGMP). Results from other studies [3–5] suggest how these domain–domain contacts might be differentially stabilized by cyclic nucleotide binding, and how conformational changes associated with nucleotide binding might bias the topology of each fulllength monomer towards or away from the trans-inhibited dimer state. Because of sequence differences, PKG lacks some key local contacts that stabilize the PKA holoenzyme R–C interface, perhaps because PKA must overcome mass action to inactivate its catalytic domain, whereas native PKG is already pre-assembled as a dimer. These differences provide starting points for rationally modulating the PKG activation constant by mutagenesis to dissect the details of the mechanism of activation. The quaternary assembly seen in the trans-inhibiting dimer of PKG Iβ differs significantly from other kinases, suggesting a unique regulation mechanism for PKG I with implications for the kinetics, cooperativity, CNB domain nucleotide selectivity, and isotype-specificity of activation.
